# An encyclopedia of human enhancer–gene regulatory interactions

**DOI:** 10.1038/s41586-026-10781-4

**Published:** 2026-07-15

**Authors:** Andreas R. Gschwind, Kristy S. Mualim, Alireza Karbalayghareh, Maya U. Sheth, Kushal K. Dey, Evelyn Jagoda, Ramil N. Nurtdinov, Wang Xi, Anthony S. Tan, James Galante, Hank Jones, X. Rosa Ma, David Yao, Dulguun Amgalan, Judhajeet Ray, Chad J. Munger, Joseph Nasser, Žiga Avsec, Benjamin T. James, Muhammad S. Shamim, Neva C. Durand, Suhas S. P. Rao, Ragini Mahajan, Benjamin R. Doughty, Kalina Andreeva, Jacob C. Ulirsch, Kaili Fan, Elizabeth M. Perez, Tri C. Nguyen, David R. Kelley, Hilary K. Finucane, Jill E. Moore, Zhiping Weng, Manolis Kellis, Michael C. Bassik, Berk Ustun, Alkes L. Price, Michael A. Beer, Roderic Guigó, John A. Stamatoyannopoulos, Erez Lieberman Aiden, William J. Greenleaf, Christina S. Leslie, Lars M. Steinmetz, Anshul Kundaje, Jesse M. Engreitz

**Affiliations:** 1 Department of Genetics, Stanford University School of Medicine, Stanford, CA USA; 2 Basic Sciences and Engineering Initiative, Betty Irene Moore Children’s Heart Center, Lucile Packard Children’s Hospital, Stanford, CA USA; 3 Department of Plant Biology, Carnegie Institute of Science, Stanford, CA USA; 4 Computational and Systems Biology Program, Memorial Sloan Kettering Cancer Center, New York, NY USA; 5 Department of Bioengineering, Stanford University, Stanford, CA USA; 6 Department of Epidemiology, Harvard T. H. Chan School of Public Health, Boston, MA USA; 7 The Novo Nordisk Foundation Center for Genomic Mechanisms of Disease, Broad Institute of MIT and Harvard, Cambridge, MA USA; 8 Gene Regulation Observatory, Broad Institute of MIT and Harvard, Cambridge, MA USA; 9 Centre for Genomic Regulation (CRG), The Barcelona Institute of Science and Technology, Barcelona, Spain; 10 Department of Biomedical Engineering, Johns Hopkins University School of Medicine, Baltimore, MD USA; 11 McKusick-Nathans Department of Genetic Medicine, Johns Hopkins University School of Medicine, Baltimore, MD USA; 12 Broad Institute of MIT and Harvard, Cambridge, MA USA; 13 Google DeepMind, London, UK; 14 Computer Science and Artificial Intelligence Laboratory, Massachusetts Institute of Technology, Cambridge, MA USA; 15 Department of Pathology, Mass General Brigham, Boston, MA USA; 16 Department of Medicine, University of California San Francisco, San Francisco, CA USA; 17 Department of Structural Biology, Stanford University, Stanford, CA USA; 18 Department of Biochemistry and Molecular Biology, University of Texas Medical Branch, Galveston, TX USA; 19 Center for Theoretical Biological Physics, Rice University, Houston, TX USA; 20 Department of Biosciences, Rice University, Houston, TX USA; 21 Program in Bioinformatics and Integrative Biology, University of Massachusetts Chan Medical School, Worcester, MA USA; 22 Calico Life Sciences LLC, South San Francisco, CA USA; 23 Department of Medicine, Massachusetts General Hospital, Boston, MA USA; 24 Analytic and Translational Genetics Unit, Massachusetts General Hospital, Boston, MA USA; 25 Stanley Center for Psychiatric Research, Broad Institute of MIT and Harvard, Cambridge, MA USA; 26 Halıcıoğlu Data Science Institute, University of California San Diego, San Diego, CA USA; 27 Department of Computer Science and Engineering, University of California San Diego, San Diego, CA USA; 28 Altius Institute for Biomedical Sciences, Seattle, WA USA; 29 Clinical Research Division, Fred Hutch Cancer Center, Seattle, WA USA; 30 Department of Applied Physics, Stanford University, Stanford, CA USA; 31 Memorial Sloan Kettering Cancer Center, New York, NY USA; 32 Stanford Genome Technology Center, Palo Alto, CA USA; 33 European Molecular Biology Laboratory (EMBL), Genome Biology Unit, Heidelberg, Germany; 34 Department of Computer Science, Stanford University, Stanford, CA USA; 35 Stanford Cardiovascular Institute, Stanford University, Stanford, CA USA; 36 Present Address: Department of Systems Biology, Harvard Medical School, Boston, MA USA; 37 Present Address: Artificial Intelligence Laboratory, Illumina Inc., San Diego, CA USA; 38 Present Address: Department of Stem Cell and Regenerative Biology, Harvard University, Cambridge, MA USA

**Keywords:** Computational biology and bioinformatics, Gene regulation, Functional genomics, Chromatin structure, Epigenomics

## Abstract

Identifying transcriptional enhancers and their target genes is essential for understanding gene regulation and the effect of human genetic variation on disease^1–6^. Here we create and evaluate a resource of more than 92 million enhancer–gene regulatory interactions across 1,458 biosamples covering 369 cell types and tissues, by integrating predictive models, chromatin states, three-dimensional contacts and large-scale genetic perturbations generated by the ENCODE Consortium^7^. We first create a systematic benchmarking pipeline to compare predictive models, assembling a dataset of 10,356 element–gene pairs measured in CRISPR perturbation experiments, more than 30,000 fine-mapped expression quantitative trait loci and 569 fine-mapped genome-wide association study (GWAS) variants linked to a probable causal gene. Using this framework, we develop ENCODE-rE2G, a predictive model achieving state-of-the-art performance across several prediction tasks, demonstrating that iterative perturbations and supervised machine learning can build increasingly accurate predictive models of enhancer regulation. Using ENCODE-rE2G, we build an encyclopedia of enhancer–gene regulatory interactions in the human genome, revealing global properties of enhancer networks, identifying differences in regulatory complexity across genes and improving analyses linking noncoding variants to target genes and cell types for common complex diseases. By interpreting the model, we find that beyond enhancer activity and three-dimensional enhancer–promoter contacts, additional features that guide enhancer–promoter communication include promoter class and enhancer–enhancer synergy. These genome-wide maps of enhancer–gene regulatory interactions, benchmarking software, predictive models and insights about enhancer function provide a valuable resource for future studies of gene regulation and human genetics.

## Abstract (web summary)

An encyclopedia of more than 92 million enhancer–gene regulatory interactions created as part of the ENCODE4 project provides a valuable resource for future studies of gene regulation and human genetics.

## Main

Noncoding DNA elements called enhancers have essential functions in controlling cell-type-specific gene regulation and cellular programs, and probably contain most causal variants for common complex diseases^4–6^. Yet, it remains an open challenge to identify accurately which elements act as enhancers in a given cell type and link them to the nearby genes that they regulate (hereafter, ‘enhancer–gene regulatory interactions’ or ‘E–G regulatory interactions’)^1–3^.

Towards this goal, the ENCODE Project has conducted thousands of experiments to identify candidate *cis*-regulatory elements and annotate their chromatin state and three-dimensional (3D) physical interactions across hundreds of cell types and tissues^7^. Using these and other data, various predictive models have been developed and applied to identify enhancer–gene regulatory interactions^8–16^. Despite recent progress, key challenges remain.

Many previous efforts are missing information on the accuracy of predictions, have not been compared systematically using common benchmarks, and/or have known limitations in prediction accuracy^6,8–12,16,17^. We need larger sets of genetic perturbation data and a community framework to evaluate, compare and develop improved predictive models of enhancer–gene regulatory interactions.

Previous predictive models have identified certain features important for prediction accuracy, including the strength of activating chromatin marks at an element (‘enhancer activity’) and the frequency at which an element physically contacts a nearby promoter (‘3D contact’)^9^. However, further analysis is needed to evaluate the utility of different experimental assays in estimating these features, to compare the relative importance of these or other molecular features and to explore how these features might inform molecular mechanisms of enhancer–promoter communication.

Some predictive models are difficult to apply to new cell types because they require analysis of many cell types simultaneously^8,11,12^ or a large number of experimental inputs in any given cell type^10^. As such, we are missing a resource of enhancer–gene regulatory interactions across the hundreds of cell types and tissues profiled by the ENCODE Project that can inform fundamental properties of gene regulation, link noncoding variants to genes, integrate with other ENCODE analysis products and expand to new cell types in the future.

Data collected in this final phase of the ENCODE Project include CRISPR perturbations of candidate enhancers^9,18–20^, high-resolution Hi-C data (manuscript in preparation) and maps of DNase I hypersensitivity across new cell types^21^—providing an opportunity to build a common benchmarking framework, build improved predictive models and apply these models to construct maps of enhancer–gene regulatory interactions in the human genome.

## Encyclopedia of regulatory interactions

**Fig. 1:**
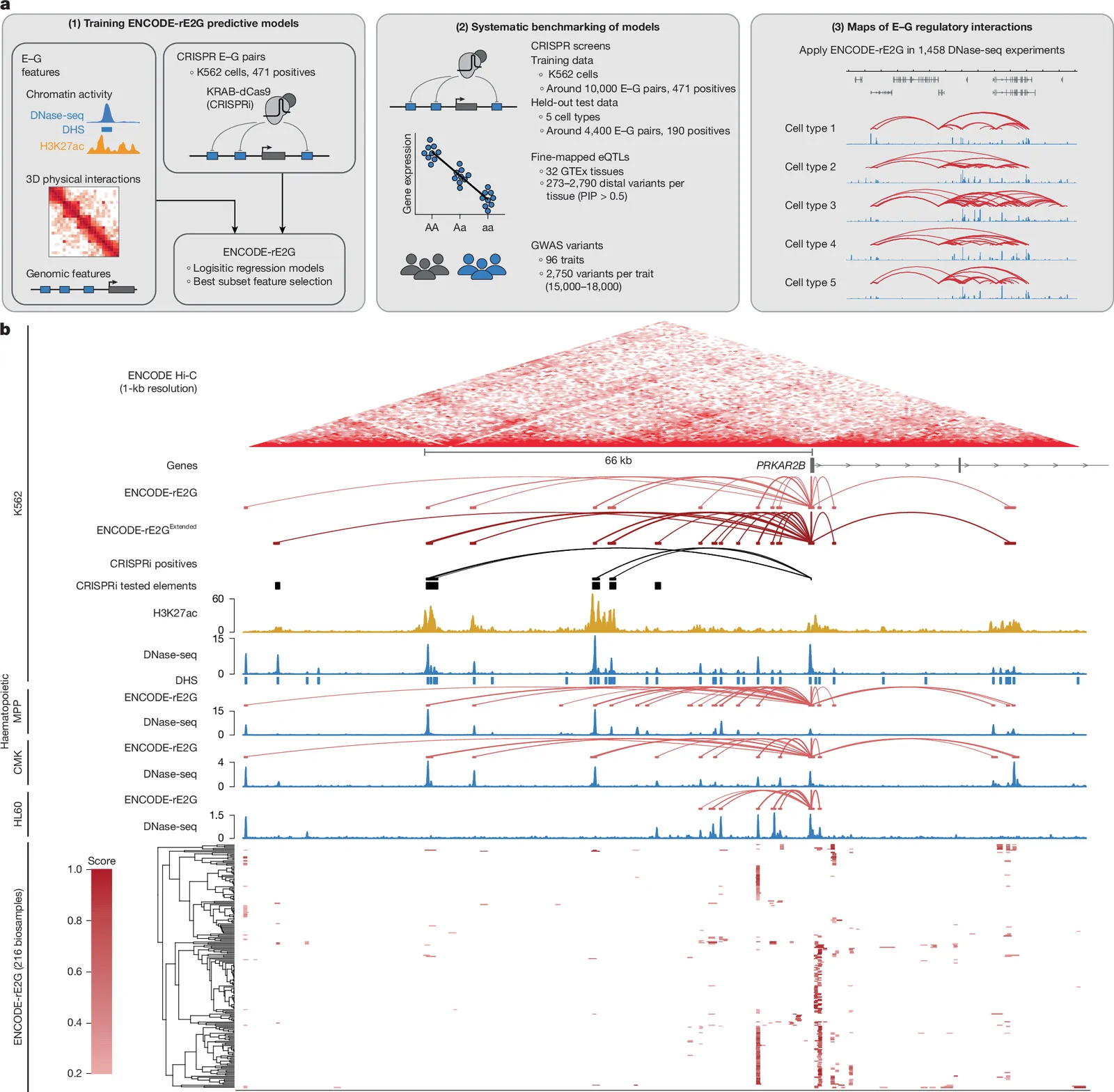
The ENCODE resource of E–G regulatory interactions. **a**, Project overview: using CRISPR data in K562 in combination with molecular features for enhancer activity, 3D physical interaction and genomic landscape, we trained new supervised predictive models on re-analysed and harmonized CRISPR data, called ENCODE-rE2G, to predict E–G regulatory connections from molecular data. We benchmarked their performance using systematic benchmarking against ground truth datasets from CRISPR, eQTL and GWAS data and compared the performance of ENCODE-rE2G against other published models. Using the ENCODE-rE2G model, we built genome-wide maps of E–G regulatory interactions across 1,458 ENCODE biosamples covering 369 unique cell types. **b**, E–G regulatory interactions for the *PRKAR2B* locus, showing detailed data and predictions in K562, DNase-seq accessibility and ENCODE-rE2G predicted E–G connections in three related cell types. ENCODE-rE2G scores are shown as a heatmap for predicted *PRKAR2B* enhancers in 216 unique cell types with at least one enhancer in the region shown. Widths of arcs are proportional to the ENCODE-rE2G scores of predicted E–G connections or *P *values of CRISPR-validated E–G pairs. DHS, DNase hypersensitivity site; MPP, multipotent progenitor.

Here we present the ENCODE resource of enhancer–gene regulatory interactions (Fig. 1), which includes three components:

1. A new set of predictive models, collectively termed ‘ENCODE-rE2G’: we developed new supervised classifiers that integrate molecular features of chromatin state and 3D physical interactions to predict enhancer–gene regulatory interactions in a given cell type. ENCODE-rE2G involves supervised training directly on CRISPR perturbation data, and enables predicting which enhancers regulate which genes in a given cell type based on various combinations of cell-type-specific experimental data (minimally, DNase-seq combined with a reference ENCODE Hi-C map averaged across many tissues).
2. A new benchmarking framework to evaluate predictive models: we collected and harmonized genetic perturbation data relevant to enhancer–gene regulatory interactions, including CRISPR perturbation experiments, expression quantitative trait loci (eQTLs) and variants from GWAS. We developed a pipeline to quantify and annotate the accuracy of predictive models and applied this to benchmark ENCODE-rE2G and hundreds of alternative models at key prediction tasks. An important difference between this and some previous benchmarking analyses is that we use as gold standards only data describing ‘regulatory interactions’, derived from genetic perturbations, as opposed to ‘physical interactions’, such as those measured by Hi-C or chromatin interaction analysis by paired-end tag (ChIA-PET) sequencing. These perturbations datasets, analysis pipelines and benchmarking results will facilitate studies to develop improved models of enhancer–gene regulatory interactions.
3. An encyclopedia of enhancer–gene regulatory interactions in the human genome: we applied the most general of the ENCODE-rE2G models to 1,458 ENCODE DNase I hypersensitive sites sequencing (DNase-seq) experiments across different cell types, tissues and contexts (collectively, ‘biosamples’) and identified 92,176,227 regulatory interactions between distal elements and target genes. This atlas includes an average of 40,210 unique regulatory elements and 63,221 element–gene regulatory interactions per biosample and, on average, annotates 0.89% of the approximately 3 billion base pairs in the human genome per biosample. This resource enables looking up a gene to find predicted enhancers, looking up an element to find predicted target genes and identifying predicted cell types and target genes for noncoding variants.

We now describe the creation, validation and application of this resource to chart global properties of enhancer–gene regulatory interactions, annotate variants associated with complex traits and identify new molecular features that tune enhancer–promoter communication.

## Model overview

Previous predictive models of enhancer–gene regulatory interactions have largely involved unsupervised approaches based on enhancer–promoter correlations^12–14^, molecular rules^9,16^ or supervised learning on proxies such as 3D loops or gene expression^8,10,11,15^. We reasoned that, by combining epigenomic data with recent large-scale enhancer-targeting CRISPR perturbation datasets, we could build an improved model by training a supervised classifier directly on CRISPR data in a single cell type, and then apply the trained model across the genome and to new cell types. We previously pioneered a similar approach^22^, but using a much smaller CRISPR perturbation dataset.

**Extended Data Fig. 1:**
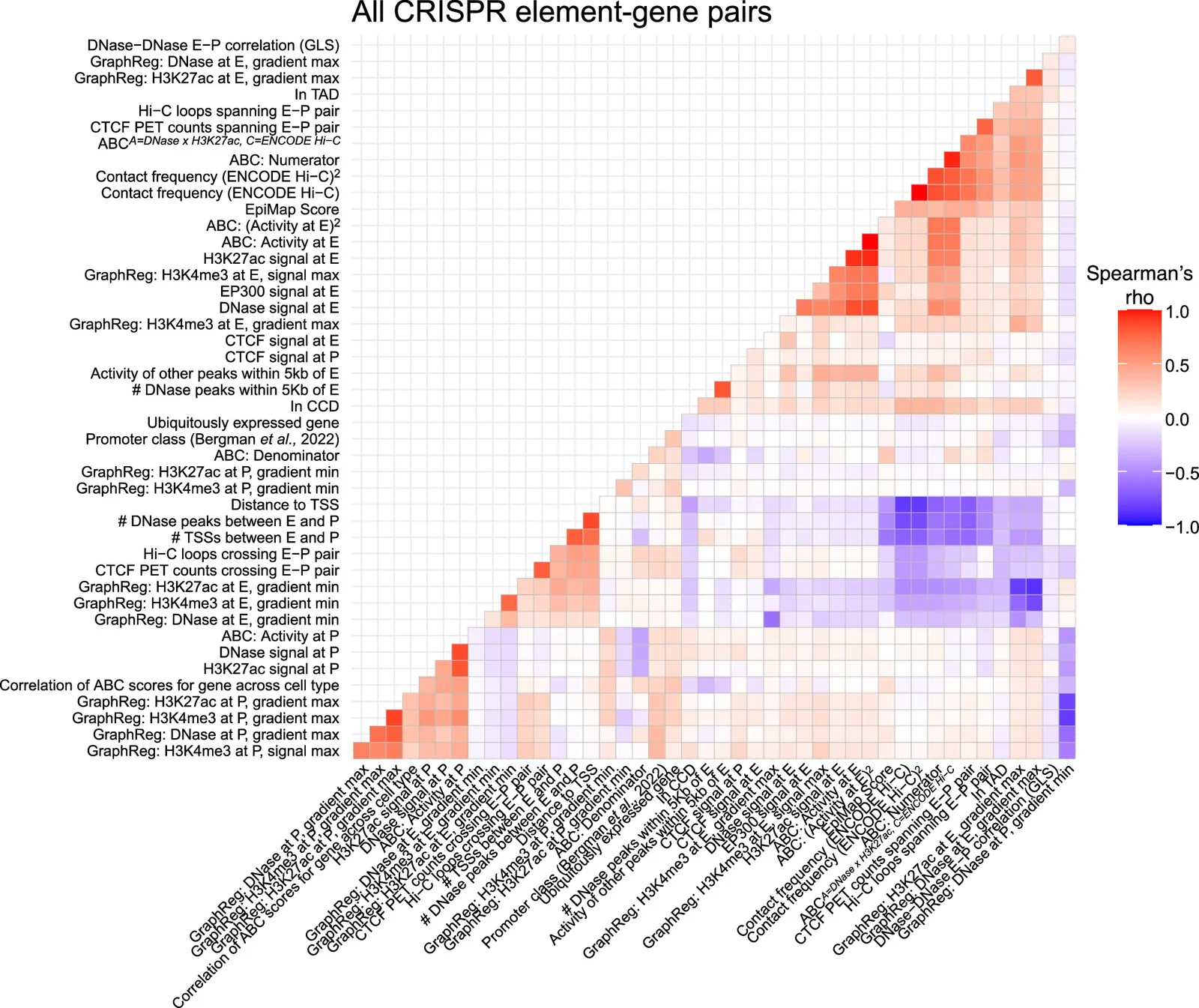
Correlation between features used as input for ENCODE-rE2G^Extended^. Correlation of collected and generated molecular features of the ENCODE-rE2G^Extended^ model for element-gene pairs in combined CRISPR dataset.

We constructed two logistic regression classifiers to predict CRISPR-validated element–gene pairs using different combinations of molecular features (Supplementary Tables 1 and 2). In the ‘ENCODE-rE2G’ model, we used features computable from only cell-type-specific DNase-seq data, thereby facilitating analyses across hundreds of ENCODE biosamples. Specifically, we included features related to (1) chromatin state (quantitative DNase-seq signals at the element and promoter), (2) 3D contact frequency (averaged across 65 ENCODE Hi-C datasets to capture cell-type-invariant genome organization); (3) the activity-by-contact (ABC) model^9^, which multiplies enhancer activity and 3D contact frequency; (4) genomic position (for example, distance from element to promoter); (5) promoter class (whether the gene is expressed uniformly and ubiquitously across cell types (that is, a ‘housekeeping’ gene)) and (6) information about nearby enhancers (for example, the activity of all other elements within 5 kb of the perturbed element) (Supplementary Table 2). Using ‘best subset’ feature analyses, we selected 8 of the 12 possible features for the final ENCODE-rE2G model (‘Molecular features guiding enhancer–gene regulation’). This approach ensures regularization while maintaining that the model outputs calibrated probability estimates^23,24^. In the ENCODE-rE2G^Extended^ model, we evaluated the utility of additional assays available in Tier 1 ENCODE cell types, and expanded to include a total of 45 features derived from DNase-seq, histone chromatin immunoprecipitation followed by sequencing (ChIP–seq), cell-type-specific ENCODE Hi-C and ChIA-PET experiments, as well as features from additional predictive models such as EpiMap^8^ and GraphReg^10^ that jointly analyse many datasets (Supplementary Table 2 and Extended Data Fig. 1).

To train and evaluate models, we aggregated a gold-standard dataset of 10,356 element–gene pairs tested with CRISPR in K562 cells. We re-analysed and harmonized data from previous studies that used genetic perturbations (mostly CRISPR interference, CRISPRi) to inhibit candidate enhancers and measure effects on gene expression^9,19,22,25,26^ (Supplementary Note 1). We developed approaches to compute statistical power for every tested element–gene pair, identifying 471 ‘positive’ unique element–gene pairs where CRISPR perturbation of the element (462 CRISPRi, 9 CRISPR deletions) led to a significant decrease in gene expression (−1 to −93% effects; Supplementary Fig. N1.1f) and 9,885 ‘negative’ element–gene pairs where no significant reduction in expression was observed despite the experiment having good power to detect effects on gene expression of more than 15–25% (Supplementary Note 1). We trained logistic regression classifiers to distinguish positives from negatives using hold-one-chromosome-out cross-validation. Then, we applied the trained model to all element–gene pairs across the genome and to new cell types.

Each element–gene pair is annotated with a score, corresponding to the probability of a regulatory effect from the logistic regression classifier. To binarize the predictions, we selected a score threshold achieving 70% recall in the CRISPR training dataset, as in previous studies^9^. At the chosen threshold, ENCODE-rE2G identified an average of 63,221 E–G regulatory interactions per biosample (108,535 for ENCODE-rE2G^Extended^), 40,210 predicted ‘enhancers’ (distal elements predicted to regulate at least one gene; 38,923 for ENCODE-rE2G^Extended^), and included well-studied enhancers such at the *HBE1*, *GATA1* and *MYC* loci (Supplementary Fig. 1).

## Systematic benchmarking of ENCODE-rE2G

**Fig. 2:**
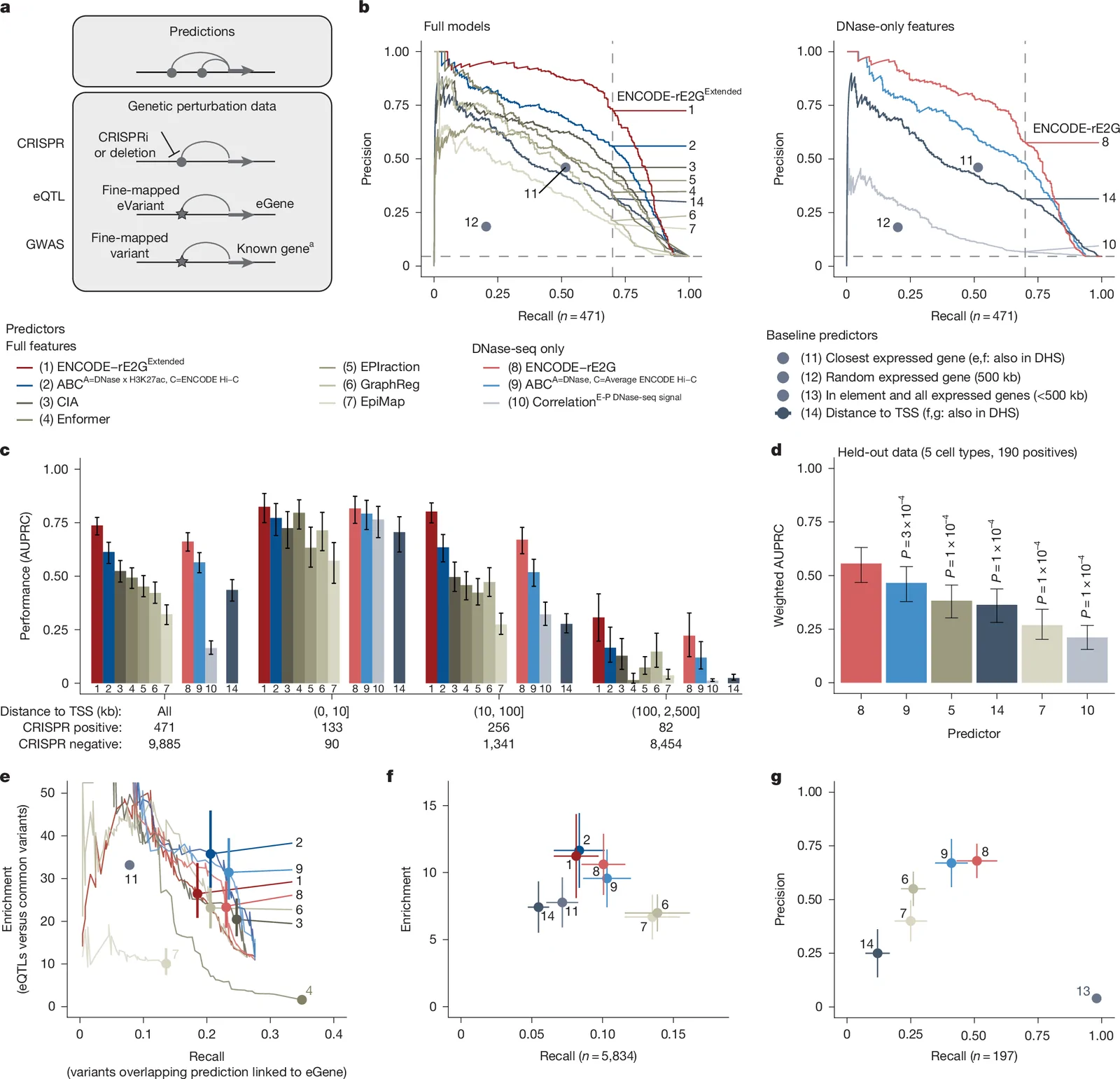
Benchmarking predictions of E–G regulatory interactions. **a**, We benchmarked predictive models using CRISPR, fine-mapped eQTLs and fine-mapped GWAS ground truth datasets. **b**, Precision-recall curves for combined K562 CRISPR data (10,356 tested element–gene pairs, 471 positives)^6,18,22^. Curves, continuous predictors; dots, binary predictors; dashed lines, 70% recall (vertical), baseline precision (horizontal). **c**, AUPRC performance of quantitative predictors for combined K562 CRISPR data versus distance to TSS. Error bars, 95% bootstrap AUPRC range (10,000 iterations). **d**, Weighted AUPRC performance of ENCODE-rE2G and other predictors for held-out CRISPR data: 4,378 total E–G pairs and 190 (157.39 weighted; Methods) positives in K562, GM12878, HCT116, WTC11 and Jurkat cells^6,31–35^. Weighted AUPRC calculated from predicted probability of direct *cis*-effects (Supplementary Note 1; Methods). Error bars, 95% bootstrap AUPRC range (10,000 iterations). *P*, significant differences (*P*_bootstrap_ < 0.05) between predictors and ENCODE-rE2G inferred using bootstraps (two-sided, 10,000 iterations; Supplementary Table 4). **e**, Enrichment-recall curves for eQTLs in lymphoblastoid cell lines. Enrichment, ratio of fine-mapped distal noncoding eQTLs (PIP > 0.5; *n* = 239) to distal noncoding common variants overlapping enhancers predicted in GM12878. Recall, fraction of eQTL variants overlapping enhancers linked to correct gene (*n* = 243). Curves, performance across score thresholds; dots, enrichment-recall at chosen thresholds; error bars, 95% confidence intervals from normal approximation on log-transformed enrichment ratios. **f**, Enrichment-recall of GWAS fine-mapped single nucleotide polymorphisms (PIP > 0.1) in predicted enhancers for matched trait-biosample pairs (*n* = 10), erythroid-related (red blood cell count, mean corpuscular volume, mean corpuscular haemoglobin and haemoglobin A1c) traits for K562; immune-related (lymphocyte count, white blood cell count, inflammatory bowel disease, monocyte count, eosinophil count, autoimmune disease-combined) for GM12878. Dots, average enrichment and recall across biosample-trait pairs; error bars, 95% confidence intervals across trait-biosample pairs. **g**, Precision-recall in blood biosamples at linking lead variants of 197 noncoding credible sets across 11 blood-related traits to putative causal genes (inferred from independent signals of coding variants within 2 Mb; Methods). Dots, average precision and recall across credible sets; error bars, 95% bootstrap confidence intervals (1,000 iterations). ^a^Independent coding variant.

To quantify and compare the performance of ENCODE-rE2G with other models, and assess how well it generalizes beyond K562, we developed a systematic benchmarking pipeline using three complementary datasets: (1) CRISPR-validated enhancer–gene pairs, (2) fine-mapped eQTLs and (3) fine-mapped GWAS variants (Fig. 2a). We used it to evaluate ENCODE-rE2G and 616 other models (Supplementary Table 1), including (1) the ABC model, re-computed using various combinations of ENCODE assays; (2) published machine-learning models, including EpiMap^8^, GraphReg^10^, constrained interaction activity (CIA)^16^, Enformer^11^ and EPIraction^27^ and (3) simple baselines such as linking elements to the closest expressed transcription start site (TSS) or gene, linking elements to genes through 3D contact, or correlating element and promoter accessibility across cell types.

**Extended Data Fig. 2:**
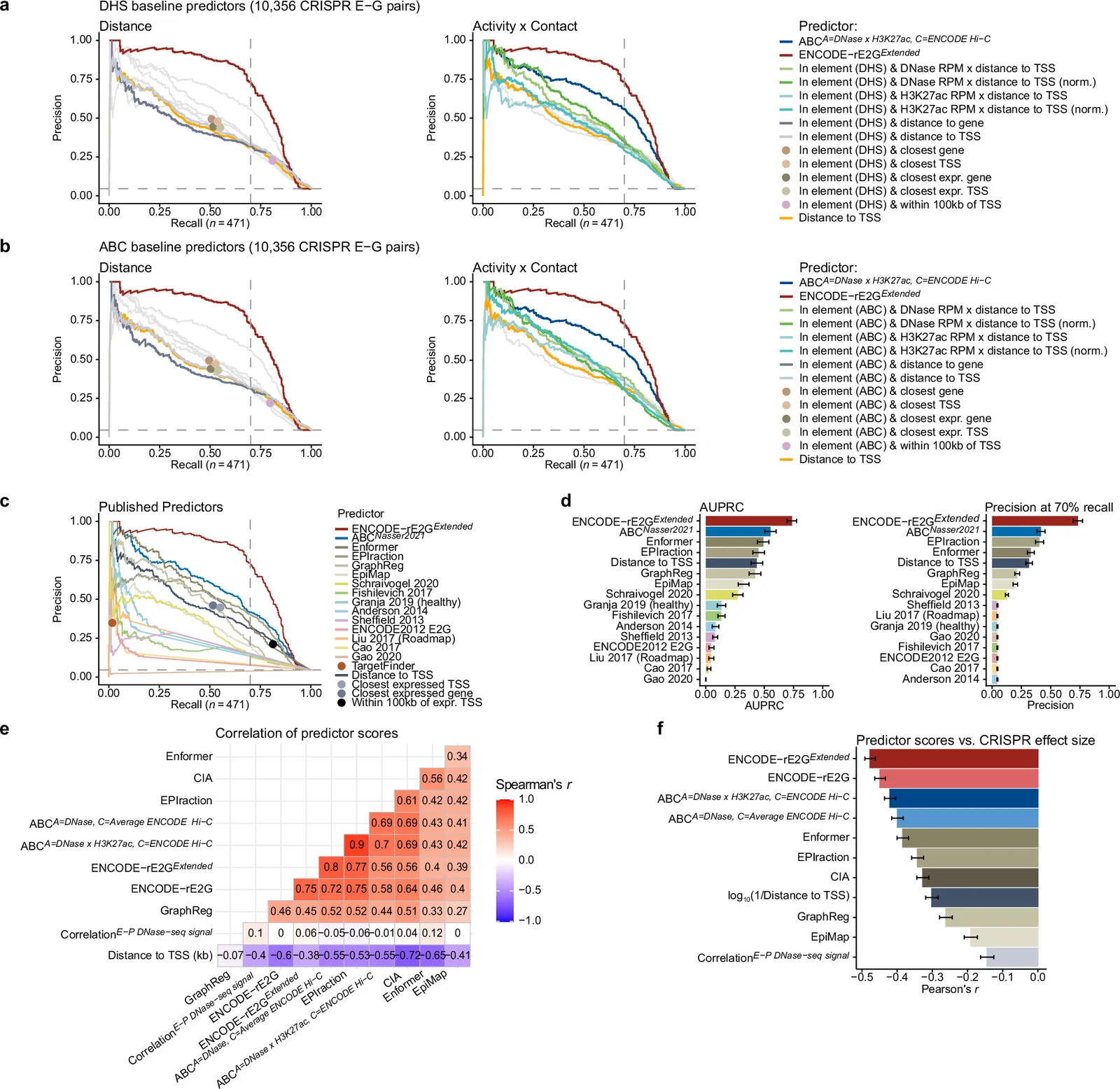
CRISPR benchmarking. **a**, Precision-Recall curves for simple baseline predictors based on ENCODE4 DNase hypersensitivity sites (DHS) grouped by genomic distance or activity (DNase-seq or H3K37ac ChIP-seq at DHS) x contact (1/distance to TSS) benchmarked on the combined K562 CRISPR data. The ABC and ENCODE-rE2G models were added as reference. **b**, Precision-Recall curves for simple baseline predictors based on ABC candidate elements grouped by genomic distance or activity (DNase-seq or H3K37ac ChIP-seq at element) x contact (1/distance to TSS) benchmarked on the combined K562 CRISPR data. The ABC and ENCODE-rE2G models were added as reference. **c**, Precision-Recall curves for previously published semi-supervised predictors benchmarked on the combined K562 CRISPR data. **d**, Area under Precision-Recall curve (AUPRC) and precision at 70% recall for previously published semi-supervised predictors benchmarked on the combined K562 CRISPR data. Error bars: 95% bootstrap AUPRC range (10,000 iterations, Methods). **e**, Correlation between predictor scores for combined K562 CRISPR E-G pairs (*n* = 10,356). *r:* Spearman correlation coefficient. **f**, Correlation between predictor scores and observed CRISPRi effect sizes for combined K562 CRISPR E-G pairs *(n* = 10,356). *r:* Spearman correlation coefficient; error bars: 95% confidence intervals.

We found that ENCODE-rE2G and ENCODE-rE2G^Extended^ achieved state-of-the-art performance across a variety of prediction tasks (Fig. 2b–g). We first benchmarked the models against the combined K562 CRISPR training dataset (Supplementary Note 1) to evaluate performance at predicting cell-type-specific causal enhancer–gene regulatory interactions by computing precision-recall curves (Fig. 2b), area under the precision-recall curve (AUPRC) (Fig. 2c and Supplementary Table 3), precision at 70% recall (Supplementary Fig. 2a) using hold-one-chromosome-out cross-validation for supervised models and correlating predictive scores with measured effects on expression (Extended Data Fig. 2f and Supplementary Fig. 3) and target gene expression levels (Supplementary Fig. 4). Between the two models only using cell-type-specific DNase-seq, ENCODE-rE2G outperformed ABC^A = DNase, C = Average ENCODE Hi-C^ by all measures (AUPRC = 0.66 versus 0.56, *P*_bootstrap_ = 0.0001; precision at 70% recall = 58% versus 48%, *P*_bootstrap_ = 0.0001; and Pearson’s *R* = − 0.45 versus −0.40, *P* = 1.44 × 10^−5^; Fig. 2b,c, Extended Data Fig. 2f and Supplementary Fig. 2a). Among models using an expanded feature set, ENCODE-rE2G^Extended^ outperformed all others, including ABC^A = DNase ^^× H3K27ac, C = ENCODE Hi-C^ (AUPRC = 0.74 versus 0.61, *P*_bootstrap_ = 0.0001; precision at 70% recall = 73% versus 56%, *P*_bootstrap_ = 0.0001 and Pearson’s *R* = 0.48 versus −0.42, *P* = 3.29 × 10^−7^; Fig. 2b,c, Supplementary Fig. 2a and Extended Data Fig. 2f). Commonly used baseline methods and other predictive models performed worse (Fig. 2b,c; detailed evaluations in Extended Data Fig. 2 and Supplementary Notes 2, 3 and 4).

**Extended Data Fig. 3:**
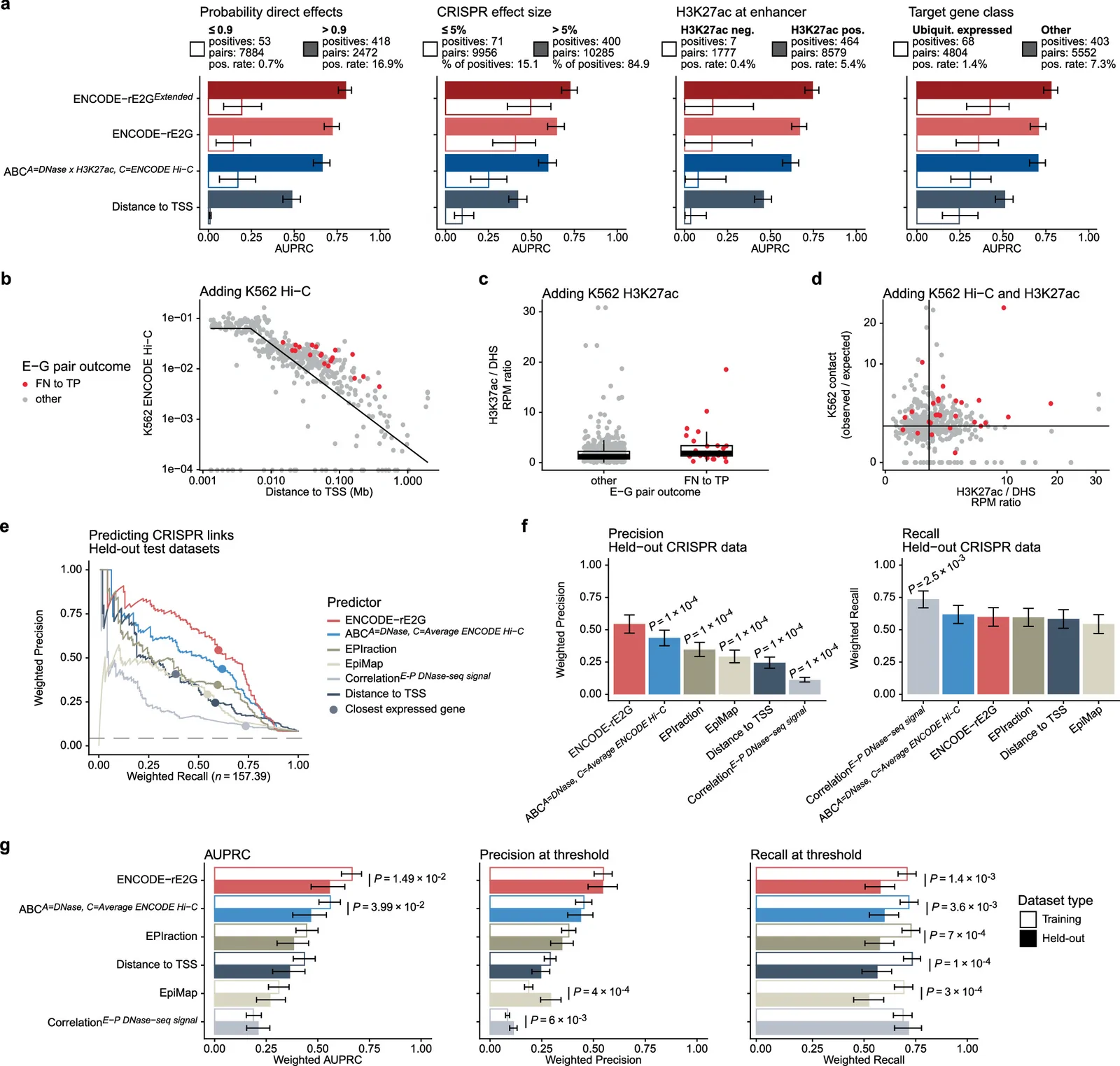
Analyzing performance of ENCODE-rE2G. **a**, AUPRC performance of ENCODE-rE2G for combined K562 CRISPR E-G pairs grouped by probability of direct effects, CRISPR effect size, element H3K27ac signal, or target gene expression uniformity. Error bars: 95% bootstrap AUPRC range (10,000 iterations). **b-d**, Properties of K562 CRISPR enhancer-gene regulatory interactions not predicted by the DNase-only ENCODE-rE2G model, but correctly predicted when adding additional assays (false negative to true positive, FN to TP). Red dots: FN to TP pairs; gray dots: other CRISPR positives. **b**, Adding cell-type-specific Hi-C correctly predicted 20 interactions with higher 3D contact than expected from a powerlaw function. Scatter plot shows K562 ENCODE Hi-C 3D contact frequency versus distance to TSS. Black line: expected powerlaw 3D contact frequency (Methods). **c**, Adding H3K27ac correctly predicted 26 interactions with higher H3K27ac relative to DNase-seq accessibility. Box plots show H3K27ac to DHS reads-per-million (RPM) ratio. Box plot elements: center line: median; box bounds: 25th and 75th percentile (interquartile range); whiskers extend to most extreme value within 1.5 x interquartile range from the box. **d**, Adding both Hi-C and H3K27ac correctly predicted 27 interactions with increased cell-type-specific 3D contact or H3K27ac relative to DNase-seq accessibility. Scatter plot shows K562 ENCODE Hi-C observed over expected powerlaw contact frequencies versus H3K27ac to DHS RPM ratio. **e**, Weighted precision-recall curves for held-out CRISPR data (4,405 E-G pairs; 190 (157.39 weighted) positives Methods). Weighted precision and recall were calculated using the predicted probability of pairs representing a direct *cis-*effect (Methods). **f**, Weighted precision and recall at chosen thresholds for held-out CRISPR data. Error bars: 95% bootstrap AUPRC range (10,000 iterations). *P:* significant differences (*P* < 0.05) between predictors and ENCODE-rE2G inferred via bootstraps (two-sided, 10,000 iterations, Supplementary Table 4). **g**, Weighted performance for training versus held-out CRISPR datasets, with effect size and chromatin state filters used to define the held-out dataset applied to both. Error bars: 95% bootstrap AUPRC range (10,000 iterations); *P:* significant differences (*P* < 0.05) between training versus held-out data, inferred via bootstraps (two-sided, 10,000 iterations, Supplementary Table 4).

Notably, all models including ENCODE-rE2G performed worse at predicting (1) interactions more likely to be from indirect effects (*P*_direct_ < 0.9), here defined as *trans*-acting or non-enhancer-mediated effects, which tend to be further away from the TSS (Supplementary Note 1 and Supplementary Fig. N1.2b); (2) interactions with small CRISPR effect sizes indicating weak enhancers not well represented in the training data; (3) elements lacking H3K27ac signals, indicating non-canonical enhancer and (4) interactions with ubiquitously expressed genes, which are less enhancer-responsive^28–30^ and exhibited a lower positive rate in the training dataset (1.5% versus 7.6%; Extended Data Fig. 3a). For example, ENCODE-rE2G and other models failed to predict three well-characterized distal *MYC* enhancers^9^ almost 2 Mb from the TSS (Supplementary Fig. 1b).

We tested generalization of ENCODE-rE2G using additional CRISPR datasets from K562, WTC11, HCT116, GM12878 and Jurkat cells (4,405 total tested E–G pairs)^6,31–35^. These used CRISPRi-based perturbation assays similar to those used in the training data, but differing in element and gene selection criteria and statistical power (Supplementary Note 1). We removed element–gene pairs shared with the training data and adjusted for the differences in experimental design (Supplementary Note 1 and Supplementary Fig. N1.2). ENCODE-rE2G achieved significantly higher AUPRC (*P*_bootstrap_ = 0.0003) and precision (*P*_bootstrap_ = 0.0001) than ABC^A = DNase, C = Average ENCODE Hi-C^ and other models (all other AUPRC and precision *P*_bootstrap_ = 0.0001; Fig. 2d and Extended Data Fig. 3e,f) and maintained similar precision (53.2%) as on the training data (54.8%) (Extended Data Fig. 3g). Thus, ENCODE-rE2G generalizes to element–gene pairs and cell types beyond the training dataset.

**Extended Data Fig. 4:**
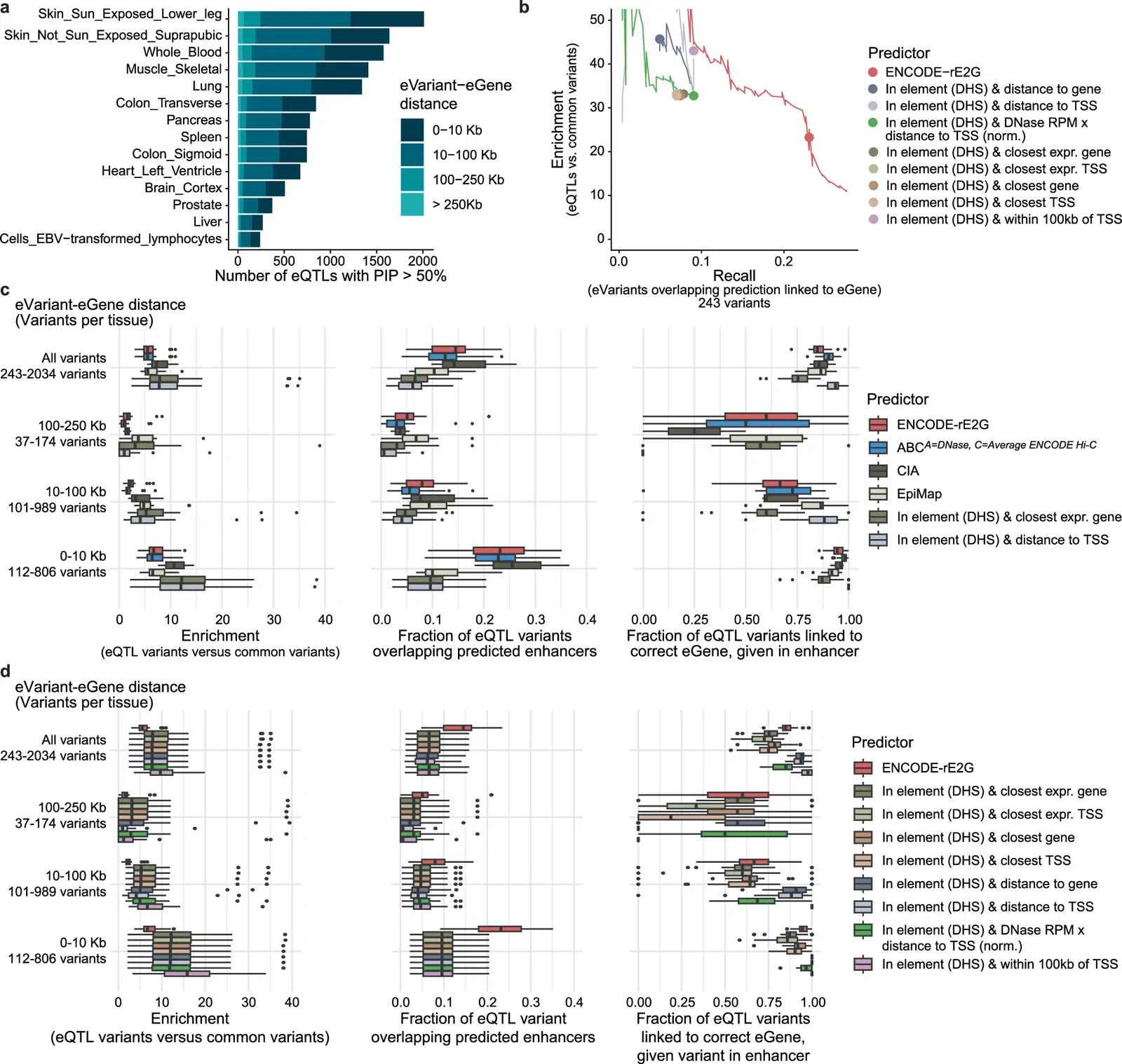
eQTL Benchmarking. **a**, Number of unique fine-mapped GTEx variants used in eQTL benchmarking analysis for each tissue, stratified be eGene-eVariant distance. Variants were filtered to those in distal noncoding regions, PIP > 0.5, in a credible set, and linked to a gene expressed in the corresponding tissue. **b**, Enrichment – recall curves showing the enrichment of fine-mapped distal noncoding eQTLs with a PIP > 0.5 in EBV-transformed lymphocytes in predicted enhancers compared to distal noncoding common variants versus fraction of variants overlapping enhancers in GM12878 cells across different score thresholds for enhancers predicted by baseline models computed on DHS elements. Numerical results are reported in Supplementary Table 5. **c**, Box plots showing enrichment (fraction of variants overlapping any enhancer/fraction of common variants overlapping any enhancer), recall (fraction of variants overlapping any enhancer), and fraction of variants overlapping an enhancer linked to the correct gene given that they overlap an enhancer for predictive models for variants from 13 tissues. Results are stratified by distance between eVariant and eGene. Box plot elements: center line: median; box bounds: 25th and 75th percentile (interquartile range); whiskers extend to most extreme value within 1.5 x interquartile range from the box. **d**, Same as (**c**), except applied to baseline predictors computed on DHS elements.

We next assessed the ability of ENCODE-rE2G to identify eQTL variants and their target genes, providing a complementary evaluation against naturally occurring perturbations across cell types and tissues. We first compared predictions in GM12878 to fine-mapped distal noncoding eQTLs from lymphoblastoid cell lines in the Genotype-Tissue Expression (GTEx) atlas^36^ (*n* = 273), computing recall at identifying eQTL variant-gene links and enrichment of eQTL variants in predicted enhancers (Methods). ENCODE-rE2G^Extended^ and ENCODE-rE2G achieved 31-fold and 25-fold enrichment at 15% recall, comparable with other well-performing models (Fig. 2e and Supplementary Table 5). Across 11 additional GTEx tissues matching ENCODE biosamples (Supplementary Table 5), ENCODE-rE2G achieved an average enrichment of 6.5 (range, 3.5–11.4), similar to other models, and achieved higher mean recall of 16% (range, 5–24%) at the chosen threshold (Extended Data Fig. 4c,d).

**Extended Data Fig. 5:**
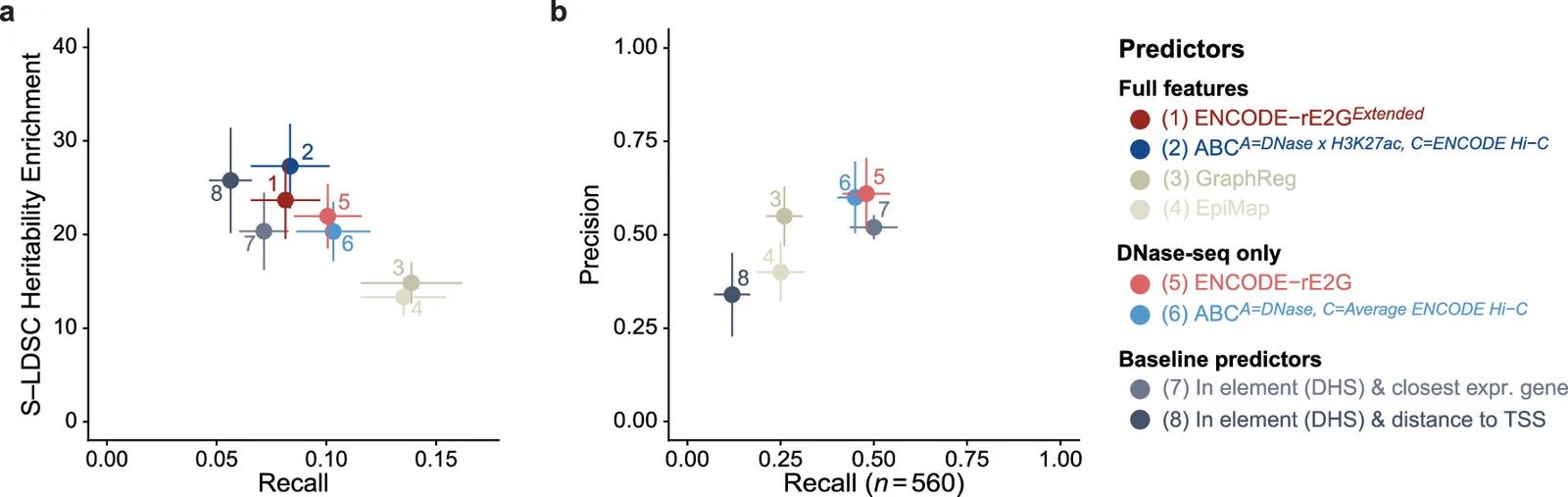
GWAS Benchmarking. **a**, S-LDSC Heritability enrichment (Y-axis) and recall of fine-mapped SNPs (X-axis) in predicted enhancers for matched trait-biosample pairs (*n* = 10): erythroid-related (RBC count, Mean corpuscular volume, Mean corpuscular hemoglobin, HbA1c) traits for K562; immune-related (Lymphocyte count, WBC count, IBD, Monocyte count, Eosinophil count, Autoimmune disease-combined) for GM12878. Dots: average heritability enrichment and recall across biosample-trait pairs; error bars: 95% confidence intervals across trait-biosample pairs. Numerical results are reported in Supplementary Table 7. **b**, A re-analysis of the benchmarking test in Fig. 2g, where we consider all noncoding credible sets for 31 traits that are linked to exactly one “putatively causal” gene with a coding fine-mapped variant within 2 Mb on either side of the lead variant (*n* = 560, Methods). Plot shows precision and recall for linking lead variants of credible sets to this gene for enhancer-gene predictions across blood biosamples. Dots: average precision and recall across credible sets; error bars: 95% confidence intervals. Numerical results are reported in Supplementary Table 7.

Finally, we tested ENCODE-rE2G and the other models at identifying GWAS variants and linking them to target genes, which, compared with eQTL genes, tend to have higher phenotypic impact and tend to be more distant^37^. Using 29,245 fine-mapped distal noncoding variants for 94 traits from the UK Biobank^38^ (Supplementary Table 6), we examined whether predicted enhancers from K562 and GM12878 were enriched for overlapping variants linked to erythroid-related or immune-related biomarker traits, respectively. ENCODE-rE2G showed strong enrichment (10.6-fold), significantly higher compared with ABC^A = DNase, C = Average ENCODE Hi-C^ (9.6-fold, *P* = 0.0001, *t-*test, *n* = 10; Fig. 2f and Supplementary Table 7). We next examined noncoding credible sets across 11 blood-related traits where a nearby putatively causal gene was identified by an independent coding variant associated with the same trait^38^ (Methods). Across 473 blood biosamples ENCODE-rE2G achieved the highest precision at identifying target genes (68% versus 67% for ABC^A = DNase, C = Average ENCODE Hi-C^), with improved recall (51% versus 41%; Fig. 2g and Supplementary Table 7). Results of additional supporting benchmarking analyses are shown in Extended Data Fig. 5.

Altogether, these results suggest that the ENCODE-rE2G models generalize well to new cell types and achieve state-of-the-art performance for a variety of tasks designed to evaluate enhancer–gene regulatory interactions. Because each benchmarking dataset represents a different type of genomic perturbation with distinct advantages and limitations (Methods), it is notable that ENCODE-rE2G performs well on all three. We share the full benchmarking pipeline to enable further refinement and comparison of predictive models (for example, scE2G^39^, AlphaGenome^40^ and EPCOT^41^; ‘Code availability’).

## Genome-wide maps of enhancer regulation

We next applied ENCODE-rE2G across 1,458 biosamples generated by the ENCODE Project, and identified a total of 92,176,227 enhancer–gene regulatory interactions using the threshold at 70% recall in the CRISPR benchmark (Supplementary Table 3). Properties of these predicted regulatory interactions inform the architecture of enhancer–gene regulation in the human genome.

**Extended Data Fig. 6:**
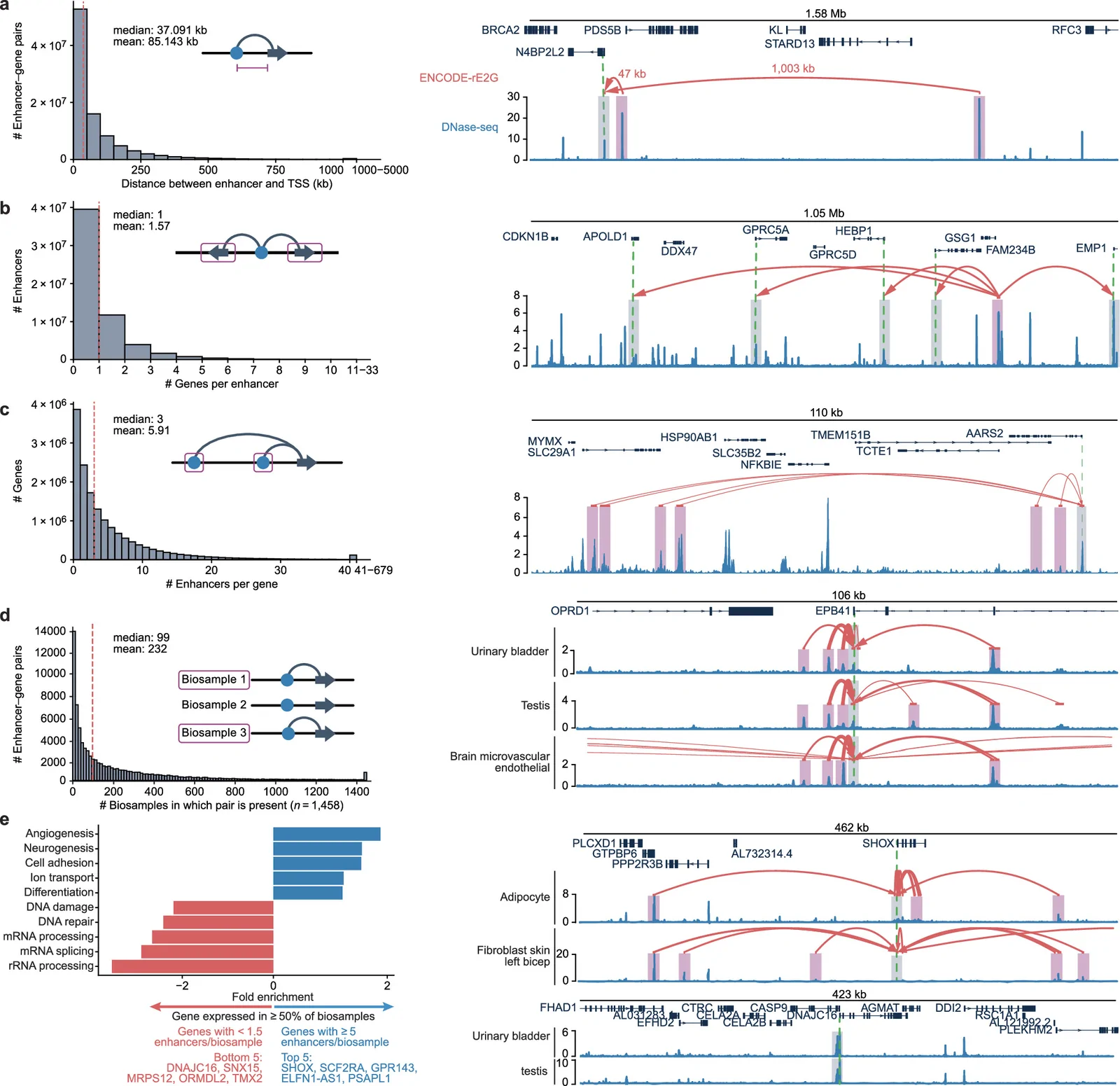
Properties of ENCODE-rE2G predictions. **a**, Distribution of distance between enhancers and target genes across 1,458 biosamples. Each data point is a predicted enhancer-gene pair in one biosample (*n* = 92,176,227 pairs). Locus plot (chr13:32,340,000–33,914,307) shows two enhancers at different distances for *N4BP2L2* in CD4-positive, alpha-beta T cells. Red dashed line, median. **b**, Distribution of number of genes regulated per enhancer across 1,458 biosamples (summary statistics in Supplementary Table 8). Each data point is an enhancer with ≥1 predicted target gene in one biosample (*n* = 58,625,467 enhancers). Locus plot (chr11:12,700,000–13,200,000) shows an enhancer regulating multiple genes in pulmonary artery endothelial cells. Red dashed line, median. **c**, Distribution of number of enhancers regulating a given gene across 1,458 biosamples (summary statistics in Supplementary Table 9). Each data point is a gene with ≥1 predicted enhancer in one biosample (*n* = 15,585,465 genes). Locus plot (chr6:44,210,000–44,320,000) shows six enhancers for *AARS2* in right ventricle myocardium inferior cells. Red dashed line, median. **d**, Distribution of number of biosamples in which a given enhancer-gene pair is predicted. Locus plot (chr21:44,300,000–44,556,000) shows enhancers for *EPB41* in urinary bladder cells (top), testis (middle), and brain microvascular endothelial cells (bottom). Arc line thickness reflects sharing across cell types: thickest = all three, middle = two, thinnest = one. Red dashed line, median. **e**, Top functional enrichments of genes expressed in >50% of biosamples, stratified by average number of enhancers per biosample. Blue: top five processes enriched in genes with ≥5 enhancers per biosample; red: top five processes enriched in genes with ≤1.5 enhancers per biosample. All shown enrichments have a Benjamini-Hochberg adjusted *P* < 0.05 (one-tailed Fisher’s exact test); fold enrichment is relative to a whole-genome background. Listed genes are the most or fewest enhancers per biosample in each group. Locus plots show *SHOX* (77.57 enhancers per biosample; chrX:277,280–739,464) and *DNAJC16* (0.005 enhancers per biosample; chr1:15,337,428–15,760,806).

**Extended Data Fig. 7:**
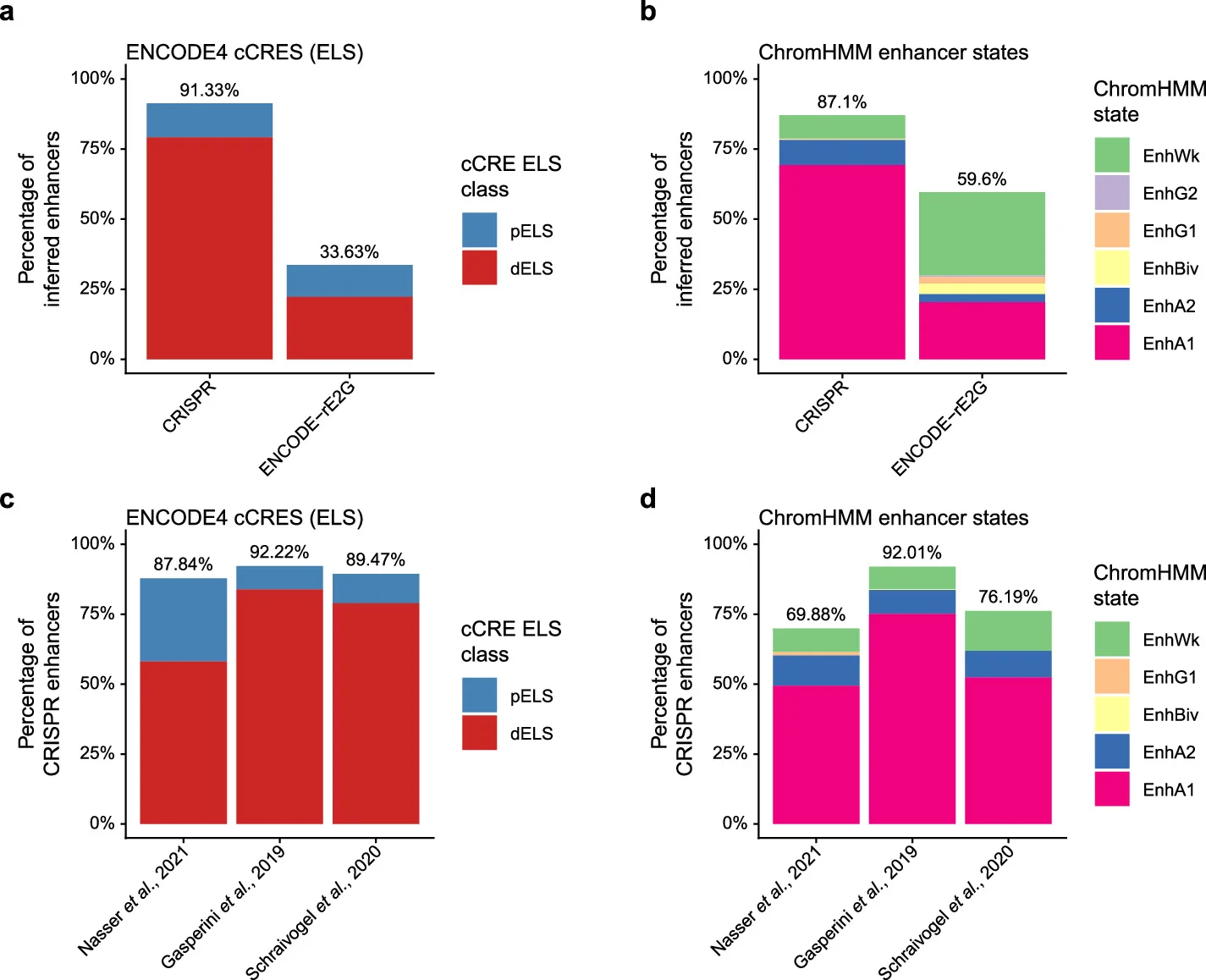
Comparison of ENCODE-rE2G to other definitions of enhancer regions. **a**, Percentage of K562 enhancers predicted by ENCODE-rE2G or inferred from CRISPR data that overlap K562 ENCODE4 proximal or distal enhancer-like signatures (pELS, dELS). **b**, Like (**a**), but for K562 ENCODE ChromHMM enhancer states. **c**, Percentage of enhancers identified in different CRISPR experiments that overlap K562 ENCODE4 proximal or distal enhancer-like signatures (pELS, dELS). **d**, Like (**c**), but for K562 ENCODE ChromHMM enhancer states.

Enhancers can be located up to millions of base pairs from their target genes^26^, but the vast majority of predicted regulatory interactions occur within shorter distances (24.1% at less than 10 kb, 74.6% at less than 100 kb; Extended Data Fig. 6a), consistent with the distance distributions of CRISPR, eQTL and GWAS results (Supplementary Fig. N2.1a). Although ENCODE-rE2G enhancers show elevated DNase-seq signal versus the set of all DNase peaks, 18.5% fall below the global median, probably because weak elements can have regulatory effects when sufficiently close to target promoters (Supplementary Fig. 5a). As a result, one-dimensional chromatin state annotations alone miss 40% (chromHMM) or 66% (cCRE Catalogue) of ENCODE-rE2G enhancers, and 30% or 12%, respectively, of validated enhancer–gene pairs from the 2021 CRISPR dataset of Nasser and colleagues^6^ (Extended Data Fig. 7). Across biosamples, each gene had, on average, 5.91 predicted enhancers (median = 3; Extended Data Fig. 6c), and each enhancer regulated 1.57 genes (median = 1; Extended Data Fig. 6b). Many predicted regulatory interactions were highly cell-type-specific (Extended Data Fig. 6d), more so than promoter or enhancer activity alone (Supplementary Fig. 5b,c). Yet, many enhancers also regulated different genes in different biosamples (Extended Data Fig. 6d). Genes with many or few enhancers across cell types showed distinct functional enrichments: among genes expressed in 50% of biosamples, those with at least five enhancers on average per cell type were enriched for cell-type-specific processes such as angiogenesis or neurogenesis, whereas genes with fewer or equal to 1.5 enhancers per cell type were enriched for housekeeping processes, such as rRNA processing and mRNA splicing (Extended Data Fig. 6e).

## Linking variants to genes and cell types

ENCODE-rE2G regulatory links can be integrated with other ENCODE resources and external data to interpret the functions of noncoding variants associated with common disease. To illustrate this, we examined fine-mapped GWAS variants from 94 UK Biobank traits.

**Fig. 3:**
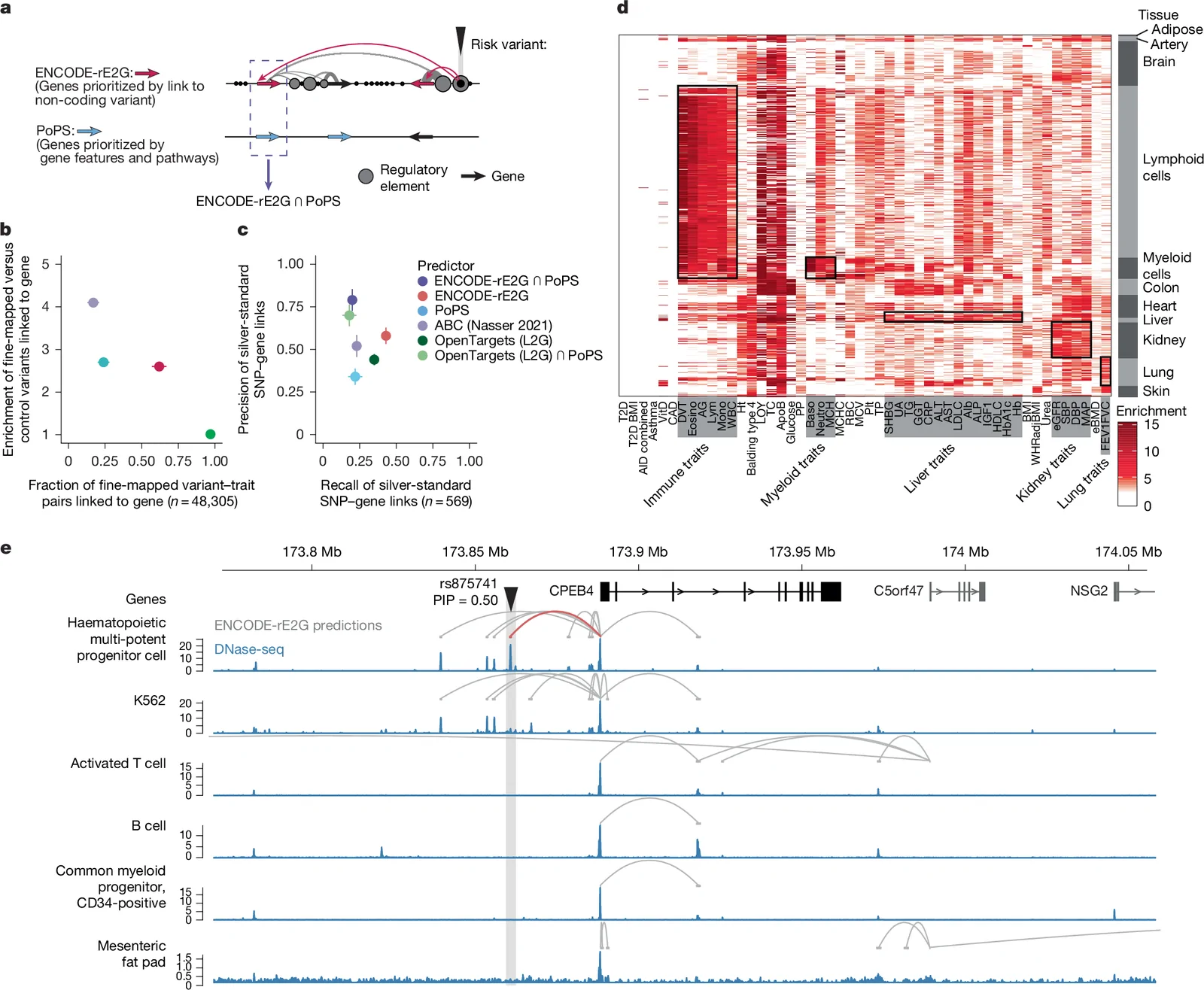
Linking noncoding GWAS variants to target genes and cell types. **a**, Schematic showing how a disease risk variant is linked to a target gene by combining ENCODE-rE2G element–gene links with PoPS gene-level disease prioritization scores (ENCODE-rE2G ∩ PoPS). **b**, Fraction of fine-mapped GWAS variants (PIP > 0.1) across 94 traits (*n* = 48,305 fine-mapped variant–trait pairs) and enrichment versus distal noncoding control variants (1000 Genomes; *n* = 9.2 million). Dots, average fraction and enrichment across traits; error bars, 95% confidence intervals based on mean and s.e. across the 94 traits. **c**, Precision-recall at identifying known causal genes for 560 noncoding credible sets, as in Fig. 2g. ENCODE-rE2G ∩ PoPS, top two ENCODE-rE2G genes for predicted enhancers overlapping PIP > 0.1 variants in the credible set intersected with top two PoPS prioritized gene in a 1-Mb window around the credible set; OpenTargets (L2G), L2G > 0.5 maps corresponding to credible sets linked through 1,512 study IDs with matching traits (Supplementary Table 10); OpenTargets (L2G) ∩ PoPS, L2G > 0.5 linked gene for GWAS credible set locus intersected with top two PoPS prioritized genes in a 1-Mb window around the credible set; ENCODE-E2G and PoPS alone, top one gene for each method. Dots, average precision and recall across credible sets; error bars, 95% bootstrap confidence intervals (1,000 iterations). **d**, Heatmap showing enrichments of GWAS fine-mapped variants (PIP > 0.1) linked to a gene by ENCODE-rE2G ∩ PoPS versus control variants for each of 50 GWAS traits (columns) and 914 biosamples mapped to distinct tissues of origin (rows). Only significant enrichments (false discovery rate (FDR) < 0.10) are shown. See Supplementary Table 6 for descriptions of GWAS traits. **e**, Example fine-mapped causal variant for mean corpuscular haemoglobin, rs875741, linked to *CPEB4* (top PoPS gene in the locus) by ENCODE-rE2G only in haematopoietic progenitors and not in other closely related blood cell types such as B cells, T cells and K562. SNP, single nucleotide polymorphism.

GWAS variants often overlapped enhancers predicted to regulate several genes (average, 2.9, maximum, 34), including 59.8% regulating different genes in different cell types. This fundamental property of enhancer–promoter regulation^6,9,18^ complicates linking noncoding variants to target genes and cell types with enhancer maps alone, and motivates combining locus-specific variant-to-gene information with orthogonal information about gene function^38,42^ (Supplementary Note 5 and Supplementary Fig. N5.1). Accordingly, we intersected genes linked to noncoding variants by ENCODE-rE2G with trait-relevant genes identified by the polygenic priority score^38^ (PoPS; Fig. 3a).

Intersecting the top two genes identified by ENCODE-rE2G and PoPS in a given GWAS locus predicted a target gene for 2,173 out of 13,012 noncoding credible sets (posterior inclusion probability (PIP) > 0.1). This included 8,234 out of 48,305 fine-mapped GWAS variants, representing a 3.71-fold enrichment in overlap compared with control variants (Fig. 3b and Supplementary Table 7). ENCODE-rE2G ∩ PoPS identified known links between credible sets and target genes with a precision of 79%, 1.4-fold and 2.3-fold higher than the top ENCODE-rE2G or PoPS gene alone (Fig. 3c and Supplementary Table 7), and attained higher precision than and comparable recall to the ABC-Max atlas^6^, including new predictions for 1,044 credible sets^6^. ENCODE-rE2G alone also achieved higher precision and recall than OpenTargets L2G^43^, and ENCODE-rE2G ∩ PoPS showed higher precision at similar recall than a combination of OpenTargets L2G ∩ PoPS (Fig. 3c and Supplementary Table 7). When investigating enrichments across biosamples, we observed that variants were enriched in ENCODE-rE2G enhancers from expected tissues (Fig. 3d and Supplementary Table 7), generalizing the results of Fig. 2f. For example, variants associated with mean corpuscular haemoglobin (MCH) were enriched 16.3-fold in haematopoietic multi-potent progenitor enhancers, and ENCODE-rE2G linked MCH variant rs875741 (PIP = 0.50) to *CPEB4* in haematopoietic progenitors but not in other cell types (Fig. 3e). This cell type specificity illustrates a key strength of ENCODE-rE2G by predicting both target genes and causal cell types of GWAS variant. *CPEB4* is expressed broadly across tissues^44,45^ and *CPEB4* knockdown was, for example, shown to inhibit both terminal erythroid differentiation^46^ and lipid accumulation in adipocytes^47^. However, the MHC-linked variant was predicted to overlap an enhancer specific to haematopoietic progenitors, pinpointing a blood-specific regulatory mechanism for this trait. Additional examples of ENCODE-rE2G linking variants to the genes *TFRC, KIT* and *BCL11A* are reported in Supplementary Note 5.

Altogether, we demonstrate how ENCODE-rE2G can be combined with information on gene function to provide accurate predictions of causal genes and cell types. We provide further analyses and guidelines for using ENCODE-rE2G to analyse GWAS loci in Supplementary Note 5.

## Molecular features guiding regulation

**Fig. 4:**
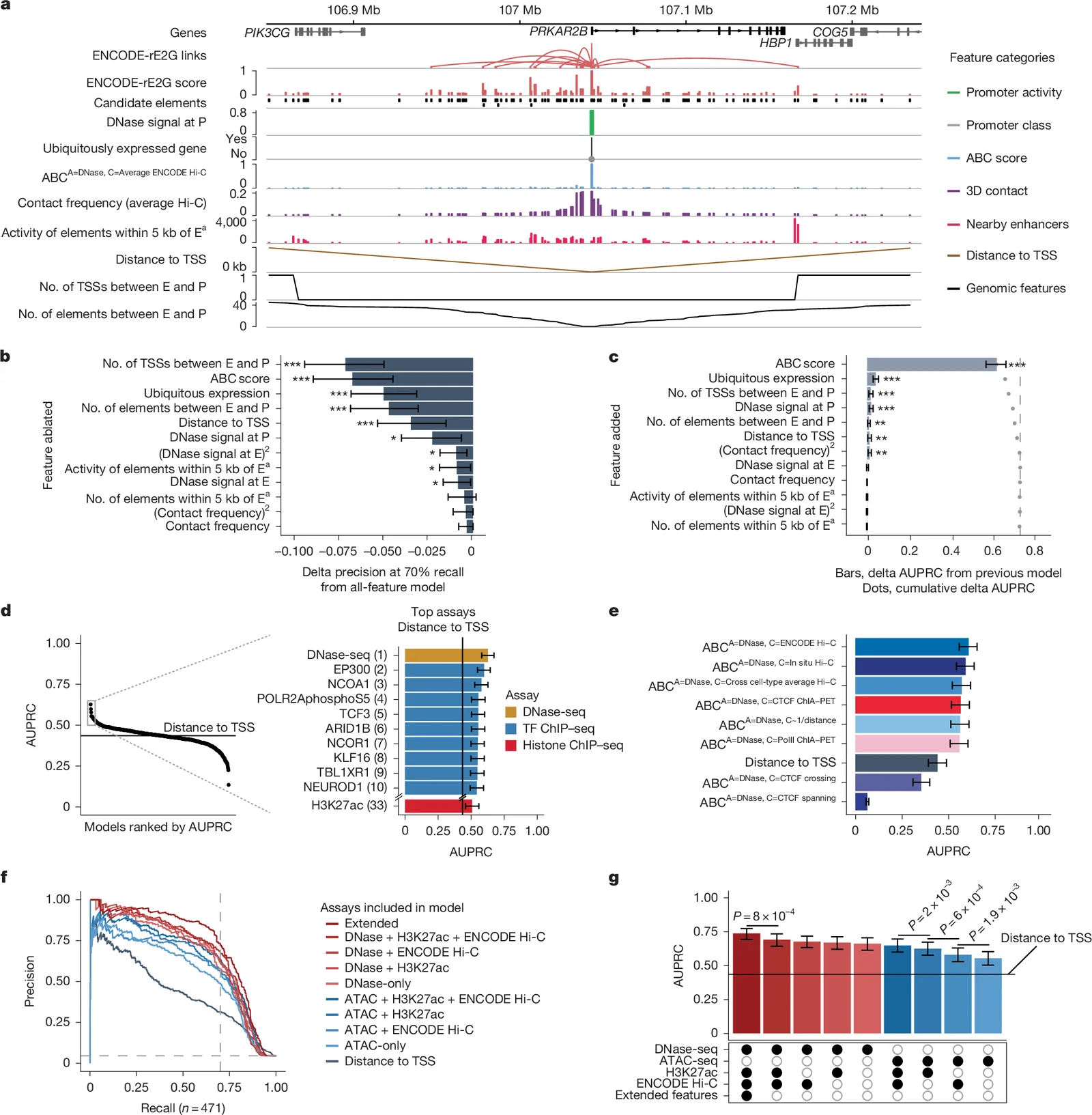
Molecular features guiding E–G regulation. **a**, Final ENCODE-rE2G features for the *PRKAR2B* locus. Only features for *PRKAR2B* E–G pairs are displayed. ^a^Features computed with the same elements counted for each candidate target gene. **b**, Leave-one-feature-out performance of ENCODE-rE2G for combined K562 CRISPR data. Each of 12 initial features is ablated by training with 11 remaining features, and precision at 70% recall (threshold from 12-feature model) is calculated. Error bars, 95% precision range inferred by bootstrap (1,000 iterations). **c**, Sequential feature selection for 12 initial ENCODE-rE2G features, selecting one feature at a time based on greatest AUPRC increase for combined K562 CRISPR data. Bars, AUPRC change from previous model; error bars, 95% precision range inferred by bootstrap (1,000 iterations); dots, total AUPRC for model including all selected features; dashed vertical lines, maximum performance reached. **d**, AUPRC performance of ABC models using 523 different ENCODE chromatin assays for the combined K562 CRISPR data. Solid lines, AUPRC of distance to TSS baseline; bar plot, AUPRC of top ten assays plus H3K27ac ChIP–seq; error bars, 95% AUPRC range inferred by bootstrap (10,000 iterations). **e**, AUPRC performance of 3D contact features for combined K562 CRISPR data. Each feature, except ENCODE-rE2G^Extended^, represents different 3D contact measurements incorporated into ABC as contact component. Error bars, 95% AUPRC range inferred by bootstrap (10,000 iterations). **f**, Precision-recall curves of ENCODE-rE2G models using different assays for combined K562 CRISPR data. Dashed lines, 70% recall (vertical) and baseline precision (horizontal). **g**, AUPRC performance of ENCODE-rE2G models using different assays for the combined K562 CRISPR data. Error bars, 95% AUPRC range inferred by bootstrap (10,000 iterations); bars, significant differences (*P* < 0.05) between closest-performing models inferred by bootstrap (two-sided, 1,000 iterations; Supplementary Table 4). *P < 0.05, **P < 0.01 or ***P < 0.001, inferred by bootstrap (two-sided; 1,000 iterations). TF, transcription factor.

While building ENCODE-rE2G, we analysed the features driving predictions to explore molecular mechanisms of enhancer–promoter communication, perform feature selection and recommend which assays are most useful for future data collection and modelling efforts. We performed a ‘best subset’ feature selection and other importance analyses (Supplementary Note 6 and Supplementary Fig. N6.1–4). Based on these analyses, the final ENCODE-rE2G model presented above uses eight features out of a larger set of 12 that we initially evaluated (Fig. 4a).

In all analyses, the most informative feature groups were 3D contact and distance, and enhancer activity (Supplementary Fig. N6.2) and the most informative individual feature was the ABC score itself (Fig. 4b,c). These observations are consistent with the importance of enhancer activity and 3D contact in enhancer regulation and highlight the utility of combining them as in the ABC model (Fig. 2b,c and Supplementary Fig. N6.3).

Beyond enhancer activity and 3D contact, features related to promoter class and enhancer–enhancer interactions also contributed to the performance of ENCODE-rE2G (Fig. 4b,c and Supplementary Figs. N6.2a and N6.3a) and ENCODE-rE2G^Extended^ (Supplementary Figs. N6.2b and N6.3b). Recent studies of enhancer–promoter sequence compatibility have shown that different classes of promoters are indeed differentially sensitive to distal enhancers^28–30^, consistent with the importance and directionality of this feature in the model (Supplementary Note 4). We explored enhancer–enhancer interactions in more detail below.

Although we designed ENCODE-rE2G to use particular assays for enhancer activity and 3D contact (including DNase-seq and Hi-C), ENCODE has collected hundreds of other assays that could be informative. Accordingly, we tested variations of the simpler ABC model in K562 cells, in which we substituted different ENCODE assays for the enhancer activity or 3D contact frequency components:

1. Out of 523 ENCODE one-dimensional chromatin experiments that could represent enhancer activity, DNase-seq was the best performing assay (Fig. 4d). ChIP–seq assays for cofactors *EP300*, *NCOA1*, *NCOR1* and several transcription factors achieved similar performance (Fig. 4d and Supplementary Fig. 6) H3K27ac was the best histone ChIP–seq assay (Fig. 4d, Supplementary Fig. 6a and Supplementary Table 3).
2. Out of six methods for estimating 3D enhancer–promoter contact frequency, the best estimate was cell-type-specific ENCODE Hi-C (precision at 70% recall = 53.3%; Hi-C depth = 23 billion reads, 5-kb resolution), which outperformed other estimates including cross cell-type average Hi-C (104 billion reads) or an inverse function of distance (precision at 70% recall = 48%, *P*_bootstrap_ = 7.62 × 10^−8^ or 44.3%, *P*_bootstrap_ = 6.35 × 10^−12^, respectively (Fig. 4e and Supplementary Note 2)). Incorporating cell-type-specific Hi-C data improved performance particularly for element–gene pairs at longer distances (for example, more than 100 kb; Supplementary Fig. N2.1g).

Towards guiding further data collection and model application to new cell types, we systematically compared the marginal utility of adding assays to ENCODE-rE2G. We constructed logistic regression classifiers with different combinations of DNase-seq, assay for transposase-accessible chromatin with sequencing (ATAC-seq), H3K27ac and Hi-C as cell-type-specific input datasets in K562 cells. Adding cell-type-specific H3K27ac and/or Hi-C data indeed improved model performance. Models using DNase-seq performed better than models using ATAC-seq (Fig. 4f,g) and adding H3K27ac or Hi-C led to a larger improvement for models using ATAC-seq (Fig. 4g), suggesting that DNase-seq measurements provide a better proxy for enhancer activity. For each of these models, the regulatory interactions that showed improved predictions showed higher than expected values for the additional assay (Extended Data Fig. 3b–d). For example, incorporating cell-type-specific Hi-C data identified 20 regulatory interactions missed by the DNase-only model, all of which showed 1.56- to 10.35-fold stronger 3D contact than expected by a power-law relationship with distance, and 1.13-fold to 4.38-fold stronger contact than estimated by the tissue-averaged Hi-C map (Extended Data Fig. 3b). Altogether, we produced pre-trained ENCODE-rE2G models for eight different combinations of input datasets, which can be used to apply to additional cell types depending on data availability (‘Code availability’).

## Super-additive functions of enhancers

Feature selection indicated that enhancer–enhancer interactions may be important to ENCODE-rE2G performance. In particular, ENCODE-rE2G includes a feature representing the sum of activities of other elements within 5 kb of the perturbed distal element, with higher nearby activities indicating a higher likelihood that the perturbed element affects gene expression. Removing this feature from ENCODE-rE2G led to a significant drop in performance (Fig. 4b, decrease in precision at 70% recall of 0.01, *P*_bootstrap_ = 0.04). The importance of nearby enhancer activity could be consistent with previous proposals that enhancers near one another in the genome can act in a synergistic manner^48–50^, and would contradict an assumption of the ABC model that enhancers act additively and independently to regulate gene expression^9^.

We explored whether, and in which cases, enhancers might have super-additive effects on gene expression—here defined as two enhancers, when added to a gene, having a larger effect than expected based on the sum of their individual effects in linear gene expression space (equivalently, such enhancers would seem to be ‘sub-subtractive’ in perturbation experiments, where combined inhibition of both enhancers leads to a smaller decrease in expression than expected from an additive model). We found several lines of evidence for such effects:

1. Of the 20 CRISPRi tiling experiments in which all nearby candidate enhancers near a gene were perturbed^9,19,26^, we identified ten genes where the sum of the effect sizes of individual enhancers linked to a given gene was greater than 100% (Fig. 5a). This indicates that at least some pairs of enhancers at these loci have super-additive effects.

**Fig. 5:**
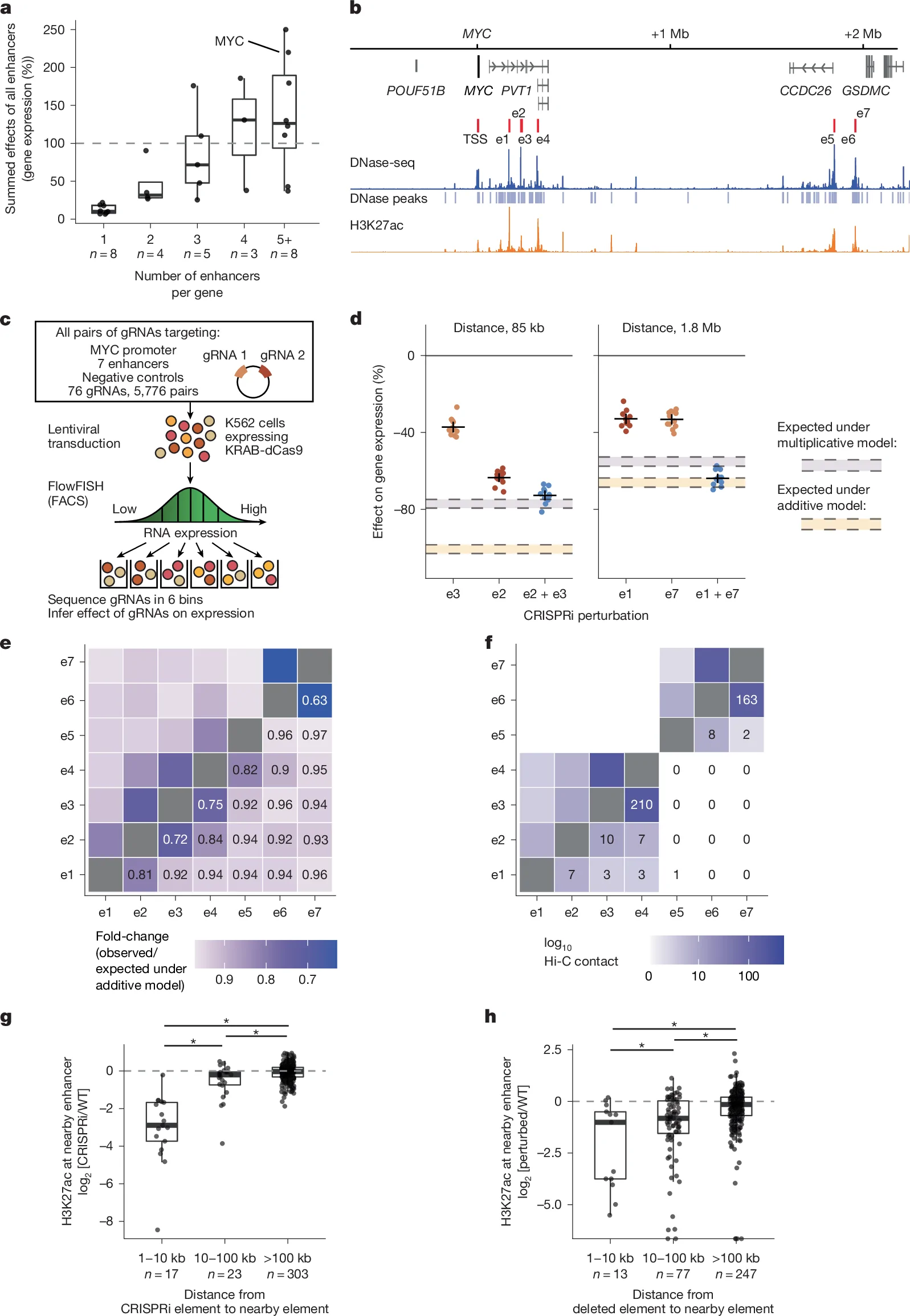
Super-additive functions of distal enhancers. **a**, Combined effect sizes on expression from CRISPRi experiments targeting all enhancers of a given gene^6,9,19^ by number of enhancers. **b**, H3K27ac and DNase-seq peaks at the *MYC* locus in K562. **c**, Experimental design: individual guides targeting a specific element at the *MYC* locus are paired with every other guide and transduced into K562-KRAB-dCas9 cells. Effects are measured with CRISPRi-FlowFISH. **d**, Individual and combined effects on gene expression from perturbing e2 and e3 (left) and e1 and e7 (right), annotated with expected effects under additive and multiplicative models (*n* = 12 guide pairs per group). Horizontal bars, mean; error bars, 95% confidence intervals. **e**, Fold-change in observed pairwise effects on gene expression versus expected pairwise effects under an additive model from paired CRISPRi screen. **f**, Normalized Hi-C contact (5-kb resolution) between *MYC* enhancers in K562. **g**, Effects of CRISPRi enhancer perturbations on H3K27ac at nearby elements, stratified by distance^26,51^. Each dot represents a perturbed enhancer × measured element pair. *Significant differences in effects (two-sided Wilcoxon rank sum exact test) between 1–10 kb and more than 100 kb (*P* = 1.79 × 10^−9^), 1–10 kb and 10–100 kb (*P* = 7.37 × 10^−6^), and 10–100 kb and more than 100 kb (*P* = 0.031). **h**, Effects of CRISPR–Cas9-mediated enhancer knockouts on H3K27ac at nearby elements, stratified by distance^52^. Each dot represents a perturbed enhancer × measured element pair. *Significant differences in effects (two-sided Wilson rank sum test with continuity correction) between 1–10 kb and 10–100 kb (*P* = 0.0077), 10–100 kb and more than 100 kb (*P* = 0.0026), and 1–10 kb and more than 100 kb (*P* = 0.00025). Box plot elements in **a**,**g**,**h**: centre line, median; box bounds, 25th and 75th percentile (interquartile range); whiskers extend to most extreme value within 1.5× interquartile range from the box. FACS, fluorescence-activated cell sorting; gRNA, guide RNA; WT, wild type.
2. We performed combinatorial CRISPRi perturbations to all pairwise combinations of seven enhancers (e1–e7) near the *MYC* gene in K562 cells using CRISPRi–FlowFISH^9^ (Fig. 5b,c and Extended Data Fig. 8). Of the 21 *MYC* enhancer pairs, 19 displayed evidence of significant super-additive interaction effects on gene expression (Benjamini–Hochberg corrected *P* < 0.05, two-sided *t*-test; Supplementary Table 11), although to differing degrees (Fig. 5d,e). Stronger interaction effects (16–37% difference from additive model) were observed for pairs of enhancers closer in genomic distance (3–89 kb) and with higher 3D contact frequency, whereas weaker interaction effects (2–10% difference from additive model) were observed for enhancers that were located farther from one another (107 kb–1.79 Mb) (Fig. 5f). This suggests that enhancers nearby to one another in the genome have super-additive effects on gene expression, whereas more distant enhancers combine approximately additively. We and others have shown that these enhancer–enhancer effects are not apparently an artefact of CRISPRi spreading (Supplementary Fig. N1.3 and Supplementary Note 1).

**Extended Data Fig. 9:**
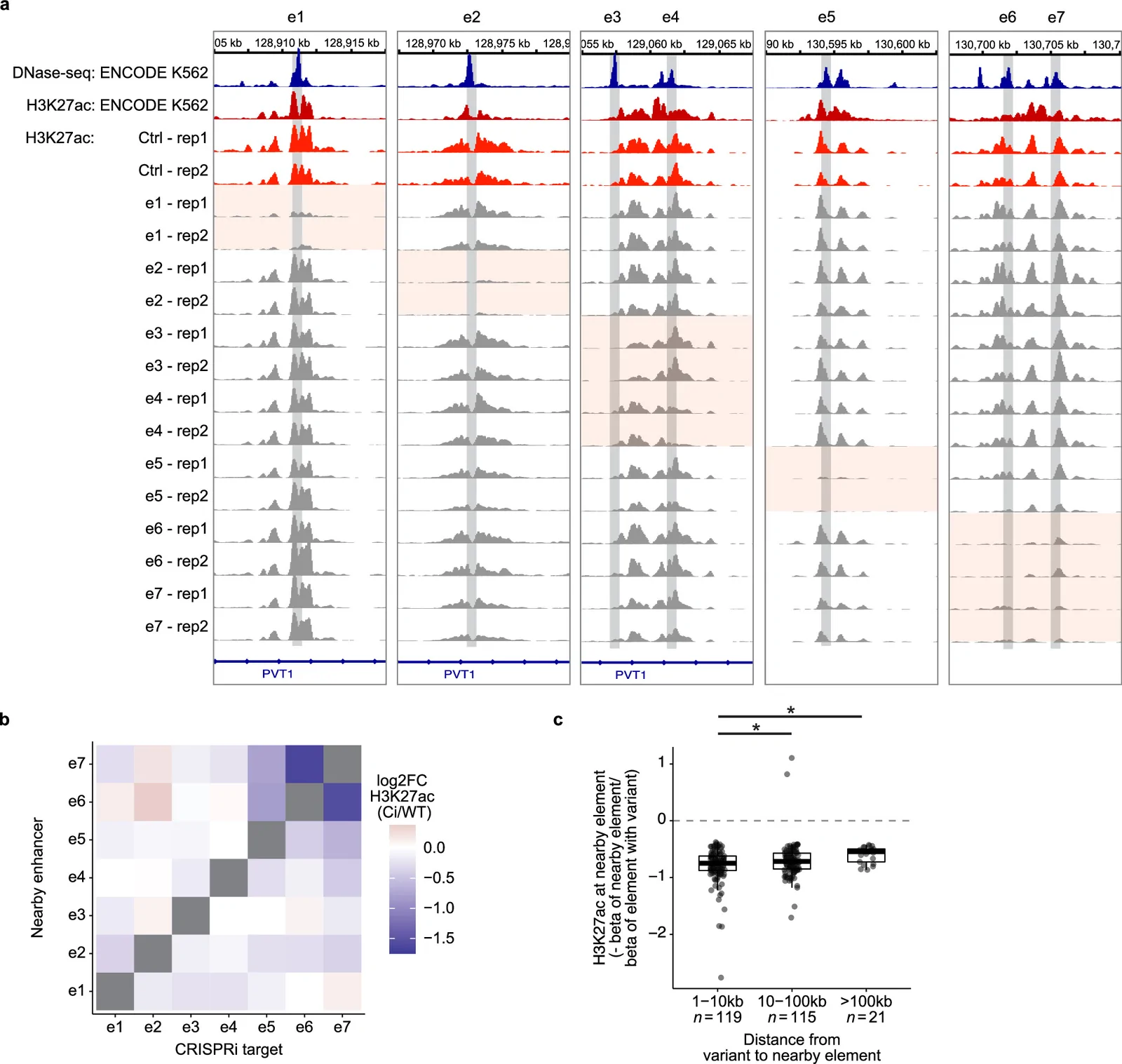
Impact of enhancer perturbations on H3K27ac at nearby elements. **a**, We performed CRISPRi-ChIP-seq at each of 7 *MYC* enhancers (e1-e7): we perturbed each enhancers in K562 using individual CRISPRi gRNAs and performed H3K27ac ChIP-seq to assess the effect on H3K27ac at other nearby enhancers. Signal tracks at top show DNase-seq (blue) and H3K27ac ChIP-seq (dark red) in wild-type ENCODE K562 datasets. Signal tracks below show H3K27ac ChIP-seq in K562 cells perturbed with a negative control gRNA (red) or a gRNA targeting one of the 7 *MYC* enhancers (gray tracks and gray vertical highlights). 2 replicate ChIP-seq experiments are shown for each enhancer. Perturbations to each enhancer successfully deplete H3K27ac at the intended target (orange highlight), as well as lead to quantitative reductions at other nearby elements. Genomic coordinates are shown in hg19. **b**, Heatmap of log2 fold-change in H3K27ac ChIP-seq signal at each *MYC* enhancer after targeting each other *MYC* enhancer from the experiment described in (**a**). **c**, Box plots showing effects of H3K27ac chromatin QTLs on other nearby H3K2ac ChIP-seq peaks relative to effect on the primary peak, stratified by distance between QTL and nearby peak^53^. Each dot represents a chromatin QTL peak x nearby peak pair. Significant differences in effects (two-sided Wilcoxon rank sum test) between 1–10 kb and 10–100 kb (*P* = 0.00685), and 1–10 kb and >100 kb (*P* = 0.0009174). Box plot elements: center line: median; box bounds: 25th and 75th percentile (interquartile range); whiskers extend to most extreme value within 1.5 x interquartile range from the box.

To further understand how nearby enhancers could have super-additive effects, we analysed experiments that combined enhancer perturbations (CRISPRi, genetic deletions or genetic variants) with H3K27ac ChIP–seq^51–53^. On average, perturbations reduced H3K27ac signal at other nearby enhancers with effects that decreased with enhancer–enhancer distance (Fig. 5g,h and Extended Data Fig. 9), providing orthogonal evidence that supports super-additive enhancer effects.

Together, these data indicate that enhancers near one another in the genome often influence each others’ chromatin state and have super-additive effects on gene expression. These effects could explain the importance of enhancer–enhancer interactions in the ENCODE-rE2G model, and highlight how interpreting the model can guide mechanistic explorations of enhancer function and highlight directions for future study.

## Discussion

Here we presented the ENCODE encyclopedia of enhancer–gene regulatory interactions in the human genome. This resource includes (1) new models that can be applied to new cell types using DNase-seq data alone or other combinations of input data; (2) benchmarking datasets and pipelines enabling systematic comparisons and iterative improvements to enhancer–gene models and (3) genome-wide maps of enhancer–gene regulatory interactions across ENCODE biosamples to identify enhancers for genes of interest, find genes regulated by enhancers of interest and interpret the functions of human genetic variants.

Identifying enhancers and their target genes has been a long-standing challenge in genomics^54^. For more than 20 years, the ENCODE Project has generated key datasets enabling the development of predictive models to address this challenge. ENCODE chromatin state maps led to the first models to link enhancers to target genes by correlating measures of enhancer activity with gene expression across tissues, cell types or conditions^13,14,55–58^. ENCODE maps of 3D chromosome contacts revealed physical interactions between enhancers and promoters^59–61^ and gave rise to predictive models aimed at identifying them^15,62^. Combining chromatin state and 3D contacts enabled the development of models that combine these features, including ABC and others^9,22^. Now, high-throughput CRISPR screens have provided a sufficiently broad survey of regulatory effects directly in the genome to enable our supervised learning framework to find optimal combinations of assays, processing methods and feature extraction approaches. This ENCODE resource of harmonized data, models and benchmarks will advance future efforts to develop models of gene regulation (including, for example, scE2G^39^, AlphaGenome^40^ and EPCOT^41^).

Our analysis, together with previous data, suggest simple rules that specify enhancer–gene regulatory interactions in the human genome. Enhancers activate promoters dependent on their 3D contact frequencies, as in the classic looping model, with contact frequencies determined by various factors including genomic distance, loop extrusion and the positions of CTCF sites^63^. The effect of perturbing an enhancer on gene expression is, to a first approximation, proportional to 3D contact frequency and the ‘activity’ of the enhancer, as described by the ABC model^9^. This effect is tuned by at least two additional factors. First, promoters of ubiquitously expressed genes are less responsive to distal enhancers (Supplementary Fig. N4.1b), which seems to be encoded in the promoter sequence^28–30^. Second, the activity of an enhancer is increased by the presence of other nearby enhancers, which seem to act super-additively with those in close 3D proximity (Fig. 5). These four factors—intrinsic enhancer activity, enhancer–promoter 3D contacts, promoter class and enhancer–enhancer interactions—together can explain a substantial fraction of the regulatory effects observed in CRISPR perturbation datasets.

Our observations regarding super-additive effects of enhancers require careful interpretation. Previous studies investigated how enhancers might combine additively, synergistically or redundantly by assessing effects at the level of gene expression^50,64–68^, cellular phenotypes^48^ or organismal phenotypes^69,70^. These previous studies have offered conflicting views, perhaps in part because these higher-order phenotypes might have varying non-linear relationships with quantitative gene expression. Here we studied pairs of enhancers using a highly quantitative assay with good power to distinguish between these models at the gene expression level (Fig. 5d–h). We observe pairwise effects ranging from approximately additive to approximately multiplicative in linear gene expression space, showing that different effects are possible even at a single gene. The interaction effects between enhancers seem to depend on 3D proximity, where enhancers that contact frequently combine super-additively and enhancers that contact less frequently combine additively (Fig. 5e,f). Notably, neither single-guide nor paired-guide CRISPR data provide evidence for enhancers having ‘redundant’ effects at the gene expression level: in the single-guide CRISPR data, many enhancers have significant individual effects, and in the dual-guide data we do not observe any sub-additive effects.

Our study highlights the importance of benchmarking models using genomic perturbation data. Here we developed and validated ENCODE-rE2G using independent datasets across several tissues and cell types, including CRISPR perturbations and genetic variants. Notably, each genetic perturbation dataset has certain strengths and limitations that provide complementary insights. CRISPR experiments identify cell-type-specific regulatory interactions by precisely perturbing individual elements, but have a limited number of positive examples in a limited number of cell types, contain biases in element and gene selection, do not distinguish direct from indirect effects, may identify non-enhancer effects, may miss elements that induce rather than maintain expression^16^ and are sometimes underpowered to detect small effect sizes (for example, <25%), and theoretically CRISPRi could affect gene expression by alternative mechanisms, for example, spreading of the repressive signal (Supplementary Note 1). The predictions of the ENCODE-rE2G model could therefore be biased in similar ways. eQTL datasets^36^ offer orthogonal evidence across many primary tissues, but lack cell type resolution, can be underpowered to detect small effects, identify regulatory mechanisms beyond enhancers^36^ and contain uncertainty in identifying causal variants. GWAS datasets test a key model application, but similarly contain uncertainty in identifying causal variants, cell types and target genes. Future efforts to train and evaluate improved models need larger, unbiased CRISPR perturbation and eQTL datasets with resolution for individual cell types, and include gene-level disease information, such as the PoPS^38^, ProGeM^71^ or V2G2P^42^. To accurately predict quantitative effects on target gene transcription, future models must account for the intrinsic strength of promoters, which can vary by several orders of magnitude^28^.

In summary, this study develops a new approach to map enhancer–gene regulatory interactions in the human genome, and provides an expansive resource for building, benchmarking and applying such maps. Our strategy included collecting and labelling high-quality genetic perturbation data, establishing benchmarking pipelines in a collaborative framework and developing predictive models that can be applied across human cell types. This strategy may be applicable more generally to learning rules of gene regulation beyond enhancer–gene regulatory interactions, including identifying the target genes of transcription factors, mapping effects of variants on chromatin state and others. Continued efforts to build such a human gene regulation map will provide a foundation for understanding the biology of health and disease, programming gene expression and interpreting the functions of genetic variants.

## Methods

This study did not involve human participants or animal subjects. The combinatorial enhancer perturbation experiment in this study used established cell lines obtained from commercial or publicly available sources. All other computational analyses used publicly available data, and are not considered human subjects research. Refer to the Supplementary Methods for more detailed descriptions of each section in Methods.

### Genome build and gene annotations

All coordinates are reported in GRCh38 unless otherwise specified. We used a curated set of 20,678 gene promoters (one per gene symbol), defined as 500-bp regions centred on the RefSeq TSS with the largest number of coding isoforms. Gene symbols were matched to ENSEMBL IDs using the HUGO database^72^ and GENCODE v.29, retaining genes annotated as protein_coding, processed_transcript or lincRNA by GENCODE.

### Biosamples and DNase-seq data processing

We computed ENCODE-rE2G and ABC predictions for 1,458 ENCODE DNase-seq experiments covering 369 unique cell types and tissues (Supplementary Table 12). Using the ENCODE API, we downloaded metadata and BAM files for released DNase-seq experiments, filtering for ENCODE4 analysis versions in GRCh38. Sequencing run type (single- or paired-ended) was determined by merging FASTQ metadata with the corresponding BAM file records. Single-ended BAM files were filtered to remove low-quality and multi-mapping reads (samtools view -F 780 -q 30) without PCR-duplicate removal, as duplicate reads are difficult to detect reliably in single-ended high-complexity DNase-seq data. Paired-ended BAM files were obtained as filtered alignments from the ENCODE pipeline, with filtering out low-quality, multi-mapping and PCR-duplicate reads (samtools view -F 1804 -q 30 -f 2 and Picard MarkDuplicates v.1.126). For experiments with mixed run types, only unfiltered alignments from single-ended reads were used. For ENCODE-rE2G^Extended^ models, additional assay input files were selected manually (Supplementary Table 13).

### Defining candidate elements and element–gene pairs

Candidate elements were defined from DNase-seq or ATAC-seq data using MACS2 (--shift −75 --extsize 150 --nomodel) on pooled replicates. After removing blacklisted regions, the top 150,000 peaks by read count were retained and resized to 500 bp centred on peak summits. TSS-centred 500-bp regions for all genes were added and overlapping regions were merged. Elements were classified as promoter (within 500 bp of any TSS), genic or intergenic. Candidate element–gene pairs included all pairs within 5 Mb.

### Annotating enhancer–promoter pairs with epigenomic and genomic features

We computed features for each element–gene pair encompassing chromatin state, 3D contact frequency and genomic position (Supplementary Table 2).

#### Chromatin state

DNase-seq and, where applicable, H3K27ac ChIP–seq read counts were computed for candidate elements, gene bodies and promoters using bedtools coverage, then quantile-normalized to a K562 reference^9^. Cell-type-specific enhancer activity was calculated as the geometric mean of normalized DNase-seq and H3K27ac signals, if included.

#### Three-dimensional contact frequency

Hi-C contact frequencies were calculated from either cell-type-specific ENCODE Hi-C data or a cell type average Megamap generated by aggregating 65 ENCODE Hi-C datasets (accession ENCSR492BKE). Processing of Hi-C contact matrices included SCALE normalization, interpolation of low-coverage bins using a power-law fit, diagonal replacement, doubly stochastic normalization and pseudocount adjustment^9^. K562 Hi-C loop calls were obtained from the ENCODE portal. CTCF ChIA-PET features (‘Loop Contain’ and ‘Loop Cross’) were computed from PET counts in K562 and GM12878 ChIA-PET data. CTCF contact domains were identified using a two-state hidden Markov model implemented in the sklearn.hmm package for Python 3.8.

#### Genomic features

Features included distance to TSS, number of intervening TSSs and candidate elements, local element density within 5 kb, enhancer complexity scores^6^, promoter class annotations^28^ and ubiquitous gene expression^28^.

### Correlation of enhancer–promoter activity across cell types

DNase-seq reads were counted at ENCODE4 candidate *cis*-regulatory elements (cCREs) and TSSs across 89 biosamples (Supplementary Table 14), normalized per million reads, and log-transformed. Pearson and Spearman correlations and generalized least squares regression coefficients (nlme v.3.1-162) were computed for cCRE–TSS pairs within 1 Mb, using a sample–sample correlation matrix to account for global inter-biosample correlations. An analogous approach was applied to DNase–RNA correlations using 96 polyA^+^ RNA-seq samples (Supplementary Table 15).

### Predictive models

#### ABC model

We applied the ABC model as described previously^9^, with updates including multi-replicate peak calling and activity estimation, ENCODE-recommended MACS2 parameters, hg38 support and a reproducible Snakemake pipeline. ABC scores were computed using DNase-only activity with Megamap Hi-C contact (ABC^A = DNase, C = Average ENCODE Hi-C^; threshold 0.018) for all 1,458 biosamples, and DNase+H3K27ac activity with cell-type-specific Hi-C (ABC^A = DNase ^^× H3K27ac, C = ENCODE Hi-C^; threshold 0.027) for K562 and GM12878 (Supplementary Table 1). The updated ABC pipeline is available on GitHub: https://github.com/broadinstitute/ABC-Enhancer-Gene-Prediction.

#### Other enhancer–gene predictive models

In addition to ENCODE-rE2G and ABC, we applied several other proposed predictive models: EpiMap^8^, EPIraction^27^, GraphReg^10^, Enformer^11^ and the CTCF loop-CIA model^16^. Each was adapted as needed to apply it to ENCODE4 data to generate predictions of enhancer–gene regulatory interactions. Details of each implementation are provided in Supplementary Methods.

### Training and applying ENCODE-rE2G

ENCODE-rE2G logistic regression models were trained on a combined CRISPR dataset in K562 containing 10,356 tested element–gene pairs, including 471 positives. For the initial model, 12 features computable from DNase-seq, average Hi-C and genome annotations were used, including both first-order and second-order terms for enhancer activity and 3D contact frequency to capture possible non-linear effects; for ENCODE-rE2G^Extended^, 45 features available in K562 and GM12878 were used (Supplementary Tables 3 and 8). Features were variance-stabilized (log[|x| + 0.01]) before fitting unregularized logistic regression models. Performance was evaluated using hold-one-chromosome-out cross-validation, holding out each of the 23 chromosomes (1–22 and X) as the test set in turn. To apply the models to new cell types, we generated cell-type-specific feature tables as described above and applied a model trained on all 23 chromosomes to each candidate element–gene pair. A Snakemake workflow for applying trained ENCODE-rE2G models to new datasets is available on GitHub: https://github.com/EngreitzLab/ENCODE_rE2G.

### Collection of published predictions and baseline predictors

We collected enhancer–gene predictions from ten previously published methods (Supplementary Table 1), lifting predictions from hg19 to GRCh38 where necessary. Details of each collected method are provided in Supplementary Methods. Baseline predictors were generated for 1,458 biosamples based on distance to TSS, nearest expressed gene and variants thereof, using both ENCODE DNase-seq narrowPeak (DNase hypersensitivity sites) and candidate elements defined here (ABC) (Supplementary Table 16). For 24 selected biosamples, additional baseline predictors were computed including distance to the gene body, nearest TSS or gene within 100 kb and DNase-seq signal (and H3K27ac signal for 20 biosamples) at the element multiplied by 1/distance. Classification thresholds for all models were set at 70% recall on the combined CRISPR data (Supplementary Table 1).

### Creating the combined K562 CRISPR training dataset

We collected three published CRISPR enhancer screen datasets in K562 from Nasser and colleagues^6^ (mostly CRISPRi-FlowFISH), Gasperini and colleagues^18^ (Perturb-seq) and Schraivogel and colleagues^22^ (TAP-seq). For Perturb-seq and TAP-seq data, we developed a unified analysis pipeline. Unique molecular identifier counts were normalized for cell-level size factors (excluding the top 10% expressed genes), log-transformed and differential expression was tested using MAST^73^ (v.1.20.0) between perturbed cells and sampled control cells, with Benjamini–Hochberg FDR correction (less than 5%). Statistical power was estimated by simulation-based analysis using negative binomial count distributions with gene-specific mean and dispersion parameters estimated by DESeq2 (ref. ^74^) (v.1.34.0), incorporating guide–guide variability in knockdown efficiency (*σ* = 0.13, learned from FlowFISH data^9^) across 20 simulation replicates. For the Gasperini dataset, guide-to-element assignments were re-computed using BLAT alignment, and candidate elements were defined from the top 150,000 K562 DNase-seq peaks. For the Schraivogel dataset, genes within the same genomic region (chr. 8 and chr. 11) were tested for each targeted element. Datasets were filtered to retain element–gene pairs with distances of 1 kb–1 Mb from TSS, excluding elements overlapping GENCODE v.29-annotated promoters or target gene bodies. Negative pairs required ≥80% power to detect a 15% decrease in expression. Negatives in the Nasser dataset were filtered for at least 80% power to detect a 25% decrease in expression based on the provided power analysis. Duplicate pairs across datasets were resolved by prioritizing significant results or, when significance status matched, highest statistical power at 25% effect size.

### Creating the combined held-out CRISPR dataset

Eight CRISPR perturbation screens across five cell types were collected for held-out benchmarking: Perturb-seq experiments in K562 (refs. ^31–33^), DC-TAP-seq in K562 and WTC11 (ref. ^75^) and CRISPRi-FlowFISH in K562 (ref. ^35^), HCT116 (ref. ^34^), GM12878 (ref. ^6^) and Jurkat^6^. Perturb-seq datasets were reprocessed uniformly with standard cell quality filtering (unique molecular identifier counts, mitochondrial transcript fraction and gene detection thresholds) and guide validation through BLAT alignment to hg38, retaining only guides mapping uniquely with 100% sequence similarity. Top 150,000 candidate elements were defined from K562 DNase-seq data using the ABC pipeline^9^. Differential expression was performed using SCEPTRE (v.0.10.0) with high multiplicity of infection settings, union guide integration, two-sided testing and 10% FDR. The above-described power analysis was adapted for SCEPTRE, testing effect sizes of 2–50% across 100 replicates per effect size. DC-TAP-seq datasets^75^ were obtained with published SCEPTRE-based results and power estimates; FlowFISH results and power analyses were obtained from the original publication^6^.

To create the final held-out dataset, duplicates within the same cell type were resolved (prioritizing significant results, then highest power), and pairs present in the training dataset were removed. Pairs were filtered by genomic location using GENCODE v.29 annotations, removing elements overlapping any annotated promoter (1 kb upstream of TSS) or the target gene body. Negatives were filtered for at least 80% power at 15% effect size (Perturb-seq/DC-TAP-seq) or 25% (FlowFISH), and positives required more than 5% effect size for Perturb-seq/DC-TAP-seq. Positives lacking H3K27ac signal (CTCF, H3K27me3 or no H3K27ac categories) were removed to focus on enhancer-mediated regulation.

### Annotating CRISPR elements with chromatin categories

Tested elements were annotated using H3K27ac, H3K27me3 and CTCF ChIP–seq peaks and quantitative H3K27ac RPM in the corresponding cell types (BAM files and peak calls from the ENCODE portal; additional ChIP–seq performed for Jurkat). Elements were categorized hierarchically based on H3K27ac peak overlap and RPM percentile as High H3K27ac, H3K27ac, CTCF, H3K27me3 or No H3K27ac (see Supplementary Methods for details).

### Calculating direct and indirect effect rates of CRISPR experiments

Indirect effect rates were estimated empirically for K562, WTC11 and uniformly reprocessed Perturb-seq datasets^18,31–33^, by testing each distal element perturbation against 100 randomly selected genes on other chromosomes, restricting to genes present in the *cis* discovery analysis to ensure comparable expression distributions. The dataset-specific indirect rate was calculated as the proportion of significant *trans*-acting tests using the nominal *P *value cutoff corresponding to the FDR threshold of each dataset in the *cis* discovery analysis. The direct effect rate as a function of distance to TSS was calculated by subtracting the distance-independent indirect rate from the observed positive hit rate, computed in 50-kb distance bins within 1 Mb of the TSS. To predict the direct effect rate for any distance, a power-law function was fitted to model direct effects versus distance using only bins with positive direct rates. For datasets where indirect rates could not be empirically estimated, the average empirical rate was used. The probability of each element–gene pair representing a direct effect was computed as:

$$\begin{array}{c}{P}_{\mathrm{direct}}=\mathrm{Probability}\,\mathrm{direct}\,\mathrm{versus}\,\mathrm{indirect}\,\mathrm{effect}\,\\ {r}_{\mathrm{direct}}=\,\mathrm{Direct}\,\mathrm{effects}\,\mathrm{hit}\,\mathrm{rate}\\ {r}_{\mathrm{indirect}}=\,\mathrm{Indirect}\,\mathrm{effects}\,\mathrm{hit}\,\mathrm{rate}\\ d\,=\,\mathrm{Distance}\,\mathrm{to}\,\mathrm{TSS}\\ {P}_{\mathrm{direct}}(d)\,=\,\frac{{r}_{\mathrm{direct}}(d)}{{r}_{\mathrm{direct}}(d)\,+\,{r}_{\mathrm{indirect}}}\end{array}$$

### CRISPR benchmark

Predictive models were benchmarked against both training and held-out CRISPR datasets. For each model, CRISPR elements were overlapped with prediction elements based on genomic coordinates (matched by gene symbol), with scores aggregated using predictor-specific functions (sum or max, Supplementary Table 1) when several predictions overlapped a single CRISPR element. Elements without overlapping predictions were assigned the minimum possible score. AUPRC and precision/recall at 70% recall were computed (ROCR v.1.0-11), with 95% confidence intervals estimated by bootstrapping (10,000 iterations; boot v.1.3-28.1). Significance of pairwise comparisons was calculated by bootstrapping delta metrics and computing two-sided *P *values by inversion of confidence intervals (boot.pval v.0.7.0). No correction for multiple testing was applied unless specified otherwise. Weighted precision-recall curves were computed using the yardstick package (v.1.2.0), weighting each pair by its estimated probability of being a direct regulatory effect (Supplementary Methods 22).

### GWAS benchmark

We assessed whether predicted enhancers were enriched for fine-mapped GWAS variants and could link those variants to probable causal genes. Fine-mapping was performed on 94 UK Biobank traits (application 31063) using SuSiE on up to 361,194 people of white British ancestry with variants meeting INFO > 0.8, minor allele frequency > 0.01% and Hardy–Weinberg equilibrium *P* > 1 × 10^−10^. Associations were estimated using BOLT-LMM (quantitative traits, inverse rank transformed) or SAIGE (binary traits), controlling for principal components, sex, age and related covariates. SuSiE allowed up to ten causal variants per region (± 1.5 Mb around lead variants), pruning credible sets at *r*^2^ > 0.25 and excluding the MHC region (chr.6, 25–36 Mb) and credible sets with variants having fewer than 100 minor allele counts. Noncoding credible sets were defined as those not containing coding or splice-site variants (within 10 bp of a splice site).

Enrichment was calculated as the fraction of noncoding variants (PIP > 0.1) in merged enhancer sets divided by the fraction of common and low-frequency 1000 Genomes variants in enhancers. Heritability enrichment was assessed using S-LDSC^76,77^, computing both enrichment and standardized effect size (τ*) for each annotation. For gene-linking evaluation, we identified 560 noncoding credible sets near exactly one gene carrying an independent coding variant (protein-truncating or damaging missense, PIP ≥ 0.5), after deduplication across genetically correlated traits, using 197 blood-trait loci as the primary benchmark. The combined ENCODE-rE2G ∩ PoPS score was computed by intersecting the top two ENCODE-rE2G gene predictions per biosample with the top two PoPS^38^ gene predictions per variant within a 1-Mb window.

### eQTL benchmark

We benchmarked models using GTEx^36^ v.8 eQTL variants fine-mapped with SuSiE^78^ (PIP > 0.5), filtered to variants in credible sets with corresponding HGNC gene symbols and linked to genes expressed above 1 transcript per million in the relevant tissue. Variants were filtered to distal noncoding regions by excluding coding sequences, untranslated regions, splice sites (within 10 bp of intron–exon junctions) and promoters (±250 bp from TSS) of protein-coding genes. GTEx tissues were matched to prediction biosamples for each model (Supplementary Table 5), with 13 tissues represented across all models used for primary benchmarking. Enrichment was defined as the fraction of eQTL variants overlapping predicted enhancers divided by the fraction of distal noncoding 1000 Genomes single nucleotide polymorphisms in predicted enhancers. Recall was computed as the fraction of eQTL variants overlapping predicted enhancers linked to the correct eGene, and enrichment-recall curves were constructed by evaluating 50–100 threshold quantiles per model.

### Assaying enhancer activity

To evaluate different activity measurements, ABC scores were computed for all element–gene pairs using 523 one-dimensional chromatin experiments in K562 (Supplementary Table 17). Each assay was substituted for DNase-seq in the activity calculation, with read-depth normalized signal (DNase-seq) or fold-change over control (ATAC-seq, ChIP–seq) bigWig files downloaded from the ENCODE portal. K562 ENCODE Hi-C was used for contact frequencies in all models.

### Linking variants to genes

The ENCODE-rE2G ∩ PoPS prediction was generated by intersecting the top two genes from ENCODE-rE2G scores for a peak overlapping a variant with the top two genes from PoPS^38^ in a 1-Mb window. PoPS prioritizes genes for a disease by training a model to predict MAGMA^79^ (v.1.08) gene scores using 57,543 gene-level functional features (bulk and scRNA-seq, biological pathways and PPI networks) in a leave-one-chromosome-out framework, making the combined score disease-specific. For each of 352 cell types and 76 diseases, the top two ENCODE-rE2G genes per biosample were intersected with the top two PoPS genes. For comparison, OpenTargets L2G > 0.5 maps were curated for 1,512 study IDs (Supplementary Table 10), and an OpenTargets ∩ PoPS combination was similarly constructed.

### Combinatorial enhancer perturbations at the *MYC* locus

A CRISPRi-FlowFISH screen was conducted to perturb all pairs of seven previously characterized *MYC* enhancers^26^ in K562 cells. A library of 10,080 paired single guide (sg)RNA vectors was designed using a lentiviral dual-promoter system (hU6 and mU6), targeting seven enhancers (e1–e7), the *MYC* TSS, two adjacent cCREs and one negative control element (NS1), with eight sgRNAs per element paired in an all-by-all fashion in both orientations, plus 80 additional vectors to calibrate recombination rates. sgRNAs were selected from previous experiments^26^ or GuideScan (CFD ≥ 0.2, efficiency ≥ 50), with 12 additional sgRNAs targeting safe harbour regions^80^.

The library (Twist Bioscience) was transduced into doxycycline-inducible dCas9-KRAB K562 cells at multiplicity of infection of approximately 0.1 (55 million cells per replicate, greater than 500-fold coverage) across two biological replicates. K562 cells were purchased from ATCC and engineered previously to express KRAB-dCas9 (ref. ^9^). After puromycin selection and doxycycline induction (48 h), cells were processed for FlowFISH using probe sets for *MYC* and *RPL13A* across seven technical replicates, sorted into six fluorescence bins on a BD Influx sorter. sgRNA pairs were amplified and sequenced on an Illumina NextSeq with custom primers. Guide pair abundances were quantified using Bowtie 2 (ref. ^81^) and Samtools^82^, and mean fluorescence estimated by maximum likelihood^9^. Effect sizes were corrected for background signal (1.57-fold correction^9^), quality-filtered (≥200 cells per guide pair per bin), and refined by selecting the two highest-effect sgRNAs per element before aggregation. Significance was assessed by two-sided one-sample *t*-tests across technical replicates. Observed dual perturbations were tested for non-additive effects using a two-sided *t*-test against 0 on the interaction term of a linear model (Effect = E1 + E2 + E1 × E2).

### Enhancer synergy analyses

To assess whether enhancer effects sum to more than 100%, we analysed 20 comprehensive CRISPRi tiling experiments^6,9,26^, retaining enhancers with significant negative effects (adjusted *P* (*P*_adj_) < 0.05) not overlapping promoters, with exceptions for two *PVT1* promoter regions shown to act as *MYC* enhancers^26^.

To examine whether enhancer perturbations affect nearby enhancer activity, we performed H3K27ac ChIP–seq following CRISPRi perturbation of seven individual *MYC* enhancers and one negative control element in K562 cells as described previously^83^ (two biological replicates per gRNA, 3–4 ChIP–seq experiments per element, 16 negative control experiments). Fold-change in H3K27ac reads per million was computed at each ABC candidate enhancer. We also analysed published datasets: CRISPRi–H3K27ac data from Fuentes and colleagues^51^ targeting long terminal repeats in NCCIT cells (625 uniquely mappable long terminal repeat regions); H3K27ac chromatin QTLs from Delaneau and colleagues^53^ and CRISPR-deletion H3K27ac data from Huang and colleagues^52^.

### Cell line authentication and mycoplasma contamination

All cell lines used in experiments performed as part of this study were authenticated through analysis of epigenetic data. All cell lines were tested for mycoplasma contamination.

### Data visualization

Plots were generated in R (v.4.2.0) using the following packages: tidyverse (v.1.3.2), ggplot2 (v.3.5.1), ggextra (v.0.10.1), ggpubr (v.0.6.0), ggcorrplot (v.0.1.4.1), ggrastr (v.1.0.2), cowplot (v.1.1.3), Gviz (v.1.42.0), GenomicInteractions (v.1.32.0).

Locus plots in Figs. 1a, 3e and 4a, Extended Data Fig. 6 and Supplementary Fig. 1 were created using the GViz (v.1.42.0) and GenomicInteractions (v.1.32.0) packages for R (v.4.2.0). Locus plots for Fig. 5b, and Supplementary Figs. N1.3 and N5.2 were created using the IGV genome browser^84^ (https://igv.org/app). Arcs in all locus plots show predicted E–G regulatory interactions above the chosen model thresholds.

### Reporting summary

Further information on research design is available in the Nature Portfolio Reporting Summary linked to this article.

## Online content

Any methods, additional references, Nature Portfolio reporting summaries, source data, extended data, supplementary information, acknowledgements, peer review information; details of author contributions and competing interests; and statements of data and code availability are available at https://doi.org/10.1038/s41586-026-10781-4.

## Supplementary information

## Figures and Tables

**Extended Data Fig. 8:**
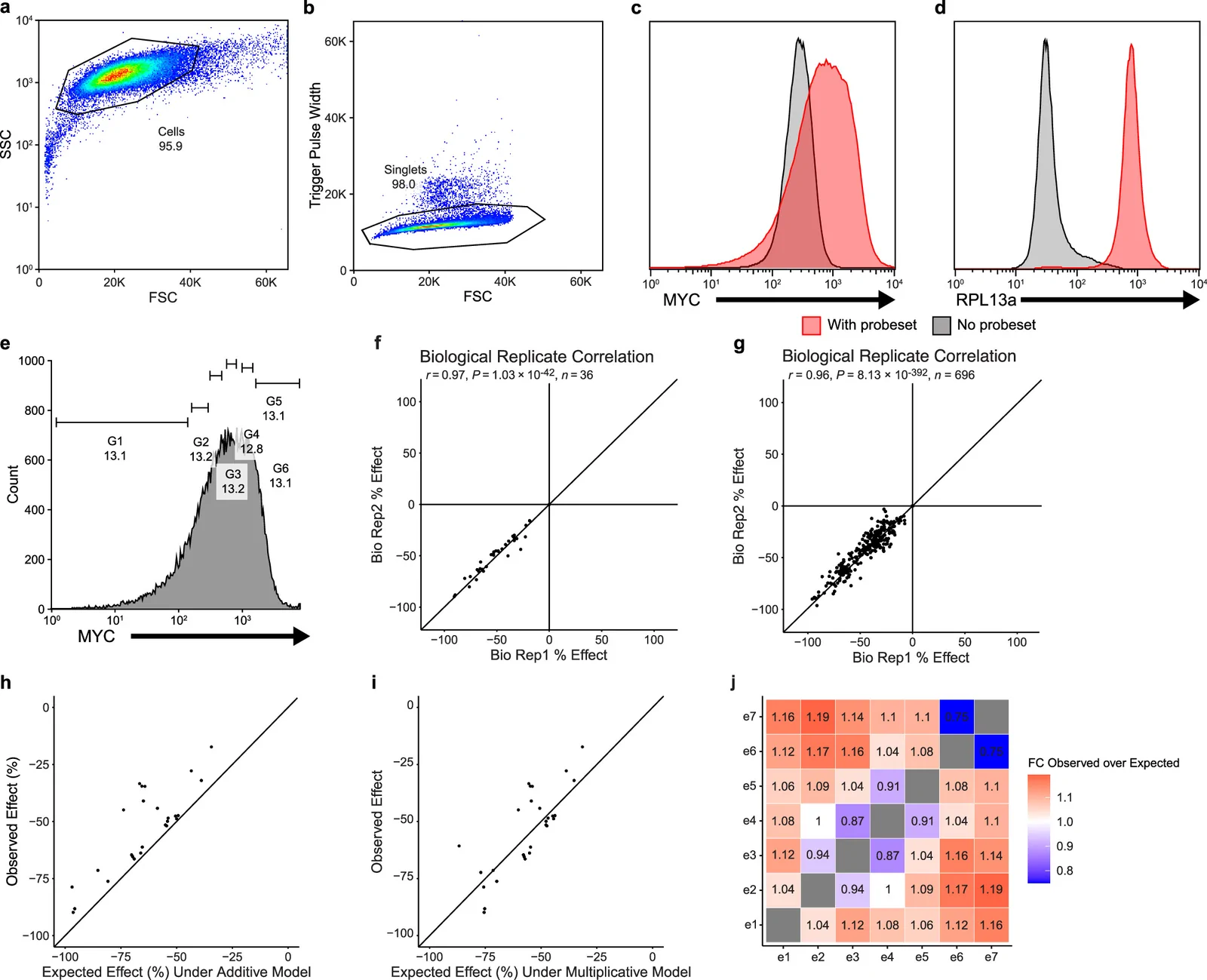
CRISPRi-FlowFISH for *MYC* to quantify super-additive enhancer interactions. **a,b**, We performed a CRISPRi screen at the *MYC* locus targeting the enhancer elements in pairs. RNA-FISH was used to measure subsequent effects on *MYC* expression. Cells were filter strained prior to flow sorting. Cells were then sorted from debris and from doublet populations based on chosen gates (black outlines). **c,d**, To measure background cellular fluorescence and confirm that our probe-sets are enriched for signal over noise, a sample was generated without introducing RNA-FISH probes (*gray*). This was compared to the average fluorescent signal from cells with probes (*red*). **e**, Representative sample of a typical sorting schema; cells were sorted into 6 bins of *MYC* expression. **f,g**, Effects on *MYC* expression were computed by comparing the non-targeting guide mean expression to the targeting guide mean expression. Biological replicate effect correlations were generated per perturbation (**f**) and biological replicate effect correlations for every guide (**g**) with a significant Pearson correlation coefficient (*r)* > 0.95 in both instances (two-sided *t*-test). **h**, Observed perturbation effects of each element pair vs. the expected effect under an additive model of enhancer-gene regulation, where the expected effect (X-axis) is generated by summing the mean effects of individually perturbing each enhancer. **i**, In this case we now multiplied the effects of the individual enhancers on gene expression to generate an expected multiplicative model of enhancer-gene regulation versus the observed effect. **j**, Heatmap of the observed effects divided by the expected effects under a multiplicative model.

## Data Availability

ENCODE-rE2G predictions and input epigenomics dataset are available on the ENCODE portal (www.encodeproject.org). ENCODE file accession IDs for predictions and inputs are listed in Supplementary Table 12. The aggregated Hi-C Megamap is available on the ENCODE portal under ENCSR492BKE. CTCF ChIA-PET data used to compute CTCF features are available on the ENCODE portal under ENCSR184YZV and ENCSR597AKG, and Hi-C data used to compute Hi-C loop features are available under ENCFF134HIZ and ENCFF173VDJ. ENCODE portal accession IDs for GraphReg and EPIraction predictions are available in Supplementary Tables 18 and 19, respectively. The combined K562 CRISPR dataset used to train ENCODE-rE2G is available at https://github.com/EngreitzLab/CRISPR_comparison/blob/v1.0.0/resources/crispr_data/EPCrisprBenchmark_combined_data.training_K562.GRCh38.tsv.gz and at https://www.synapse.org/Synapse:syn70814727 for a version including all ENCODE-rE2G^Extended^ features. The dataset is also available on the ENCODE portal under ENCSR998YDI. The held-out CRISPR dataset is available at https://github.com/EngreitzLab/CRISPR_comparison/blob/v1.0.0/resources/crispr_data/EPCrisprBenchmark_combined_data.heldout_5_cell_types.GRCh38.tsv.gz. CRISPR enhancer perturbation data for the individual datasets are available under GSE120861 (ref. ^18^), GSE135497 (ref. ^22^), https://github.com/EngreitzLab/ABC-GWAS-Paper (ref. ^6^), GSE171452 (ref. ^33^), GSE129837 (ref. ^32^) and ENCFF904ZDX (ref. ^31^). The ENCODE K562 DNase-seq experiment used to define candidate elements for CRISPR Perturb-seq data re-analyses is available on the ENCODE portal under ENCSR000EOT. For the eQTL benchmarking pipeline, GTEx eQTL variants and RNA expression data for each tissue are available on Synapse at https://www.synapse.org/Synapse:syn70198274. For the GWAS benchmarking pipeline, fine-mapped GWAS data for UK Biobank traits is available from https://www.finucanelab.org/data. Files containing baseline predictors used for benchmarking analyses can be found on Synapse at https://www.synapse.org/Synapse:syn58896208. Data from the pairwise CRISPRi enhancer perturbation experiment at the *MYC* locus in K562 are available on the ENCODE portal under ENCSR443VTK. The used gRNA library is available under ENCFF118BUP. H3K27ac ChIP–seq data from ref. ^52^ used in enhancer synergy analyses are available from GEO: GSE107726. CRISPRi–H3K27ac ChIP–seq experiments at the *MYC* locus are available from GEO GSE225157.

## Code availability

The following software was used: ENCODE-rE2G from https://github.com/EngreitzLab/ENCODE_rE2G; ABC from https://github.com/broadinstitute/ABC-Enhancer-Gene-Prediction; GraphReg from https://github.com/karbalayghareh/GraphReg; CRISPR benchmarking pipeline from https://github.com/EngreitzLab/CRISPR_comparison; eQTL benchmarking pipeline from https://github.com/EngreitzLab/eQTLEnrichment; GWAS benchmarking pipeline from https://github.com/Deylab999MSKCC/e2g-benchmarking; EPIraction from https://github.com/guigolab/EPIraction; CIA and CCD features from https://github.com/wangxi001/CIA; EpiMap from https://github.com/KellisLab/EpiMap_GRCh38_linking; combined K562 CRISPR data (training data) processing from https://github.com/argschwind/ENCODE_CRISPR_data; held-out CRISPR data processing from https://github.com/EngreitzLab/ENCODE_Test_Dataset_Analysis; and manuscript analysis code from https://github.com/EngreitzLab/ENCODE-Distal-Regulation-Paper.
