# Supplementary material for: An encyclopedia of human enhancer–gene regulatory interactions
Source: Nature. 2026 Jul 15;657(8130):179–90. doi: 10.1038/s41586-026-10781-4 (PMC13471189; doi:10.1038/s41586-026-10781-4)
Supplement: Supplementary file 1 [file 41586_2026_10781_MOESM1_ESM.pdf]

nature portfolio

https://doi.org/10.1038/s41586-026-10781-4

Supplementary information

An encyclopedia of human enhancer–gene regulatory interactions

In the format provided by the authors and unedited

Supplementary Information

Supplementary Notes **4**
Supplementary Note 1. Analysis of the integrated CRISPR datasets 4
Supplementary Fig. N1.1 | Generation of the combined K562 training CRISPR dataset 8
Supplementary Fig. N1.2 | Generation and analysis of the held-out CRISPR dataset 10
Supplementary Fig. N1.3 | Spreading of CRISPRi repressive signal 11
Supplementary Note 2. Evaluating features of 3D genome organization 12
Supplementary Fig. N2.1 | Impact of 3D contacts on enhancer regulation 14
Supplementary Note 3. Evaluating features of correlation between enhancer and promoter activity 16
Supplementary Fig. N3.1 | Informativeness of correlations in enhancer-promoter activity across cell types 18
Supplementary Note 4. Evaluating differences between ubiquitously and specifically expressed genes 20
Supplementary Fig. N4.1 | Differences in distal regulation between gene/promoter classes 21
Supplementary Note 5. Using ENCODE-rE2G maps to interpret GWAS signals 23
Supplementary Fig. N5.1 | Linking common variants fine-mapped from GWAS data by ENCODE-rE2G. 25
Supplementary Fig. N5.2 | Example GWAS loci linked to target genes by the ENCODE-rE2G ∩ PoPS 27
Supplementary Note 6. Feature selection and importance in ENCODE-rE2G models 28
Supplementary Fig. N6.1 | Performance of different ENCODE-rE2G feature subsets by subset size 30
Supplementary Fig. N6.2 | Feature categories ablation on ENCODE-rE2G and ENCODE-rE2G^{Extended} 31
Supplementary Fig. N6.3 | Sequential feature selection on ENCODE-rE2G and ENCODE-rE2G^{Extended} 32
Supplementary Fig. N6.4 | SHAP scores of ENCODE-rE2G and ENCODE-rE2G^{Extended} models 33
Supplementary Fig. 1 | K562 gene locus plots 34
Supplementary Fig. 2 | Additional CRISPR benchmarks 35
Supplementary Fig. 3 | Correlation between predictor scores and CRISPR effect sizes 36
Supplementary Fig. 4 | Correlating ENCODE-rE2G predictions with target gene expression levels 37
Supplementary Fig. 5 | Properties of enhancer-gene regulatory interactions 38
Supplementary Fig. 6 | Features of enhancer activity 40
Supplementary Table Descriptions **41**
Supplementary Table 1 | Collected enhancer-gene predictions 41
Supplementary Table 2 | Features of enhancers, promoters, and enhancer-promoter pairs 41
Supplementary Table 3 | Performance of predictive model on the CRISPR benchmark 41
Supplementary Table 4 | Pairwise comparisons of predictive models on the CRISPR benchmark 41
Supplementary Table 5 | eQTL benchmarking 41
Supplementary Table 6 | GWAS traits 41
Supplementary Table 7 | GWAS benchmarking results 41
Supplementary Table 8 | ENCODE-rE2G summary statistics per biosample 41
Supplementary Table 9 | ENCODE-rE2G summary statistics per gene 41

Supplementary Table 10 | OpenTargets L2G traits 41
Supplementary Table 11 | Pairwise *MYC* enhancer perturbation screen results 41
Supplementary Table 12 | Input datasets for ENCODE-rE2G and ENCODE portal Accession IDs 42
Supplementary Table 13 | Input datasets for training ENCODE-rE2G and ENCODE-rE2G^{Extended} 42
Supplementary Table 14 | Input files for DNase-DNase correlation 42
Supplementary Table 15 | Input files for DNase-RNA correlation 42
Supplementary Table 16 | Input data for baseline predictors Synapse URLs 42
Supplementary Table 17 | Enhancer activity ABC input files 42
Supplementary Table 18 | Input datasets for GraphReg and ENCODE portal Accession IDs 42
Supplementary Table 19 | Metadata for EPIraction 42
Supplementary Methods 43
1. Genome build 43
2. Gene reference file for ENCODE-rE2G 43
3. Defining a set of biosamples for ENCODE-rE2G and ABC enhancer-gene predictions. 43
4. Downloading and pre-processing DNase-seq data for ENCODE-rE2G and ABC predictions 43
5. Defining candidate elements and element-gene pairs for predictive models and annotations 44
6. Annotating enhancer-promoter pairs with epigenomic measurements and genomic features 45
7. Correlation of enhancer-promoter activity across cell types 49
8. Applying the Activity-by-Contact model to ENCODE4 data 50
9. Applying EpiMap to ENCODE4 data 51
10. EPIraction 52
11. GraphReg 52
12. Enformer 53
13. CTCF loop-constrained Interaction Activity (CIA) 53
14. Training and applying ENCODE-rE2G logistic regression models 54
15. Collection of previously published enhancer-gene predictions 54
16. Generating baseline predictors 56
17. Defining classification thresholds for predictive models 57
18. Creating the combined K562 CRISPR training dataset 57
19. Analyzing held-out CRISPR datasets 59
20. Annotating CRISPR elements with chromatin categories 62
21. Calculating direct/indirect effect rates 63
22. CRISPR benchmark 64
23. GWAS benchmark 67
24. eQTL benchmark 69
25. Benchmarking limitations 71
26. Additional analyses: Assaying enhancer activity 71
27. Additional analyses: Linking variants to genes analyses 72
28. Additional analyses: Combinatorial enhancer perturbations at *MYC* locus 72
29. Additional analyses: Enhancer synergy analyses 75
30. Additional analyses: Enhancer-promoter correlation 77

31. Data visualization 77
Supplementary References **78**

Supplementary Notes

Supplementary Note 1. Analysis of the integrated CRISPR datasets

CRISPR interference (CRISPRi) screens that target distal candidate regulatory elements, defined by accessible chromatin sites, and read out through growth phenotypes or gene expression have become an essential tool for probing gene regulation[1–11]. These experiments enable targeted knockdown of individual regulatory elements, allowing researchers to observe their effects on gene expression. Unlike expression quantitative trait loci (eQTL) studies — another high-throughput approach for studying gene regulation — CRISPRi screens are not confounded by the effects of linkage disequilibrium, which can complicate pinpointing regulatory interactions to specific regulatory elements in close proximity with each other. CRISPR enhancer perturbation screens have shown that detected enhancer-gene regulatory connections[3,4,10] and quantitative effects on expression[1,3,10] are reproducible.

We note several considerations and caveats when interpreting these data and using them to train models:

1. The vast majority of the CRISPR datasets we analyze here involve using CRISPRi (dCas9-KRAB). The repressive chromatin modification H3K9me3 deposited by dCas9-KRAB can spread from the intended target into other regulatory elements, leading to the detection of false-positive effects. Dox-inducible dCas9-KRAB expression is a widely used tool to control the duration of the activity of the repressive machinery and limit any potential spreading[1,11]. Studies looked at spreading of the repressive signal in distal element targeting CRISPRi screens, and have found little evidence of H3K9me3 spreading beyond 1kb from the targeted accessible chromatin site (**Supplementary Fig. N1.3**)[1,12,13]. In addition to CRISPRi, six enhancer perturbations in the combined K562 training data used in this study are from genetic deletions of single enhancers[14–16], showing an effect on the expression of 9 different target genes, and are in line with previous studies showing that quantitative effects from CRISPRi agree with effects from genetic deletions of the same elements[3,13,17].

2. The experimental design of different large-scale CRISPRi screens can influence the type of regulatory interactions detected. For example, the number of cells analyzed per perturbed element and the sensitivity of the phenotypic readout, *i.e.,* expression levels of nearby genes, can impact the statistical power to detect small effects on gene expression. This can bias experiments towards detecting enhancers with predominantly large effects on their target genes and missing enhancers with smaller effects[10]. The selection of candidate elements with particularly high enhancer activity signatures, *e.g.*, H3K27ac[18], will increase the number of detected positives, but bias the results towards detecting strong regulatory elements. Conversely, selecting a more random set of DNase-seq accessible sites as candidate elements will investigate a better representation of the genome landscape, but will decrease the positive hit rate and increase the proportion of non-enhancer effects, such as from perturbing CTCF sites[10].

3. Of note, CRISPR enhancer screen experiments do not discriminate between direct, *cis*-acting effects of enhancers (*i.e.,* enhancers regulating target promoters via 3D contact[2]) and other indirect effects on gene expression. Indirect effects as defined here include trans-acting effects and effects mediated by non-enhancer elements. For example, a perturbed regulatory element can directly regulate a nearby gene that in turn regulates another gene via a *trans*-acting effect, which is also detected in the experiment (also see Ray *et al.*, 2025[10]), or perturbing a CTCF site can affect gene expression by rearranging the 3D contact landscape.

Based on these limitations, we therefore cannot exclude that different CRISPR enhancer screens have varying statistical power to detect enhancer effects on target genes (**Supplementary Fig. N1.1b,c**),

and that some of the observed effects are caused by perturbing non-enhancer elements (**Supplementary Fig. N1.2a**) or indirect effects (**Supplementary Fig. N1.2b**), which might bias benchmarking analysis and training models using these datasets. When analyzing and harmonizing enhancer CRISPRi screens for this study, we performed a series of analyses outlined below to take these limitations into account.

Generating harmonized CRISPR enhancer perturbation datasets

We collected and reprocessed 3 previously published CRISPRi enhancer screen datasets from Nasser *et al.*, 2021[9] (mostly CRISPRi FlowFISH), Gasperini *et al.*, 2019[3] (Perturb-seq) and Schraivogel *et al.*, 2020[4] (TAP-seq) (**Supplementary Fig. N1a**). These different experiments applied different candidate element selection strategies, leading to different characteristics and possible different performance of E-G predictive models (**Supplementary Fig. N1e**). The CRISPRi FlowFISH experiments in Nasser *et al.*, 2021[9] applied a tiling approach where all DHS sites within five loci were perturbed with a median of 55 guide RNAs, and also incorporated data from other published studies. Gasperini *et al.*, 2019[3] targeted a selection of active enhancers across the genome with 2-4 guide RNAs. Lastly, Schraivogel *et al.*, 2020[4] targeted DHS sites within two genomic regions on chromosomes 8 and 11 and with four guide RNAs each. In an attempt to standardize the analysis of these datasets, we analyzed all experiments on the level of DHS sites, e.g. individual guide RNA perturbations were aggregated per targeted DHS.

To analyze the single-cell RNA-sequencing based datasets from Gasperini *et al.*, 2019 and Schraivogel *et al* 2020, we developed an analysis pipeline centered around performing differential expression tests of perturbed vs. non-perturbed cells for each target enhancer (**Supplementary Methods 18**, https://github.com/argschwind/ENCODE_CRISPR_data). Depending on the sensitivity of the molecular readout, the number of perturbed cells per targeted element and the expression levels of target genes, CRISPRi screens can have differing statistical power to detect the effect of CRISPRi perturbations on gene expression levels. In practice this can lead to tested element-gene pairs with insufficient statistical power to detect changes in expression of enhancer perturbations. This introduces false negatives, which will negatively impact benchmarking, where E-G pairs are treated as either true positives or true negatives. To filter out any potential false negatives from the collected CRISPR screens, we implemented a simulation-based power analysis that estimates the statistical power of each tested E-G pair to detect specific effect sizes on gene expression levels upon perturbation (e.g. 25% reduction in expression). A similar approach was previously applied in the analysis by Nasser *et al.*, 2021[9]. For effect sizes below 25%, we observed less than 80% statistical power for the majority of tested E-G pairs in both Gasperini *et al.*, 2019[3] and Schraivogel *et al.*, 2020[4] **(Supplementary Fig. N1b,c**). For our benchmarking analyses we filtered out any negatives with less than 80% power to detect an effect size of 15% for Gasperini *et al.*, 2019[3] and Schraivogel *et al.*, 2020[4], and 25% for Nasser *et al.*, 2021[9].

In the re-analyzed and power-filtered datasets we observed a total of 471 positive and 9,885 negative E-G pairs (**Supplementary Fig. N1.1d**)**,** covering a total of 3,942 candidate elements and 2,171 nearby candidate target genes from the used list of genes (**Supplementary Methods 18**) (**Supplementary Fig. N1d**). We observed a wide-range of measured effect sizes for detected enhancer-gene links ranging from 1% - 93% reduction of target gene expression levels upon CRISPRi perturbations (**Supplementary Fig. N1.1f**), with stronger effects observed for enhancers closer to the target gene TSS. Notably, 360 of the positive pairs come from Gasperini *et al.*, 2019, which tested elements with high activity levels (**Supplementary Fig. N1.1e**), leading to a potential bias for strong, high activity enhancers in the combined CRISPR training dataset. The re-analyzed CRISPR data is available on the ENCODE portal under the accession number ENCSR998YDI and as part of the CRISPR benchmarking pipeline under:

https://github.com/EngreitzLab/CRISPR_comparison/blob/main/resources/crispr_data/EPCrisprBenchmark_combined_data.training_K562.GRCh38.tsv.gz.

To evaluate the performance of ENCODE-rE2G and other models on an independent, unseen, set of CRISPR data, we re-analyzed and harmonized data from several additional published enhancer screens in K562[5–7,10,11], WTC11, HCT116[8], GM12878[9] and Jurkat[9] cells. We processed all these datasets using an adapted version of the differential expression testing and power simulation strategy used to analyze Perturb-seq experiments in the combined K562 training dataset (**Supplementary Methods 19**). In this held-out dataset, we identified a total of 249 positive and 4,215 negative enhancer-gene regulatory interactions across the 5 cell types (**Supplementary Fig. N1.2a**). The combined held-out CRISPR data is available at:
https://github.com/EngreitzLab/CRISPR_comparison/blob/main/resources/crispr_data/EPCrisprBenchmark_combined_data.heldout_5_cell_types.GRCh38.tsv.gz.

Assessing the performance of ENCODE-rE2G and other models

To better understand how experimental design decisions affect the types of detected regulatory interactions in all collected CRISPR datasets, which impact benchmarking analyses, we overlapped perturbed elements with different ENCODE chromatin assays[10] (**Supplementary Methods 20**). We observed that the combined held-out dataset contained more CRISPR positives that overlapped CTCF sites or did not overlap H3K27ac peaks than the training data (**Supplementary Fig. N1.2a**), indicating higher proportions of effects from other regulatory elements than true enhancers.

Next, we estimated the probability that a given E-G pair in a CRISPR experiment represents a direct, *cis*-effect versus an indirect effect (see above) using a previously developed analysis procedure (**Supplementary Methods 21**)[10]. This approach is based on the assumption that the rate of direct effects depends on the genomic distance between perturbed elements and target gene promoters (**Supplementary Fig. N1.1f**), whereas indirect effects occur at a distance-independent rate. We first estimated indirect effect rates by testing for effects of candidate element perturbations on genes on other chromosomes, which we assume to be indirect effects or false discoveries. Next, we calculated the direct effect rates by subtracting dataset specific indirect effect rates from the observed hit rates as function of distance to TSS, averaged direct effect rates across datasets and fit a powerlaw function to predict direct effect rates as function of distance to TSS. Finally, we derived a probability of each E-G pair to be a direct effect by calculating the difference between the predicted direct effects rate as function of distance to TSS and the estimated or imputed indirect effects rate for respective dataset (**Supplementary Fig. N1.2b, Supplementary Methods 21**). This analysis revealed different probabilities of direct effects between datasets (**Supplementary Fig. N1.2b**), with CRISPR positives in the held-out dataset being more likely the result of indirect effects than in the training dataset (**Supplementary Fig. N1.2c**).

We adjusted CRISPR benchmarking analyses on the held-out CRISPR data for these properties to ensure that the set of ‘positive’ examples represent true *cis*-acting enhancer-gene regulatory interactions. To do so, we 1) removed any CRISPR positive elements without H3K27ac signal or overlapping CTCF sites or <5% effect size on target gene expression (to ensure that positives in the held-out dataset represent true enhancers with reasonable effect sizes, which is what ENCODE-rE2G is trained to predict) and 2) computed performance metrics weighted by the probability of direct effects to adjust for effects from likely indirect effects (**Supplementary Methods 22**).

ENCODE-rE2G achieved significantly higher AUPRC ($P_{bootstrap}$ = 0.0003) and precision ($P_{bootstrap}$ = 0.0001) at the chosen threshold than ABC$^{A=DNase,\ C=Average\ ENCODE\ Hi\text{-}C}$ and other available models on the held-out CRISPR dataset (**Fig. 2d, Extended Data Fig. 3e,f**) and achieved similar recall as ABC$^{A=DNase,}$

$^{C=Average\ ENCODE\ Hi\text{-}C}$ (**Extended Data Fig. 3f**). ENCODE-rE2G also achieved similar precision at the chosen threshold on the held-out and training datasets (**Extended Data Fig. 3g**). Recall ($P_{bootstrap}$ = 0.0014) and therefore also overall AUPRC ($P_{bootstrap}$ = 0.0149) performance remained lower on the held-out compared to the training data, however this was also the case for other predictors including $ABC^{A=DNase,\ C=Average\ ENCODE\ Hi\text{-}C}$ and a distance to TSS baseline predictor (**Extended Data Fig. 3g**). Taken together these results demonstrate that ENCODE-rE2G maintains its superior performance over other predictors in an unseen, held-out CRISPR data across multiple cell types.

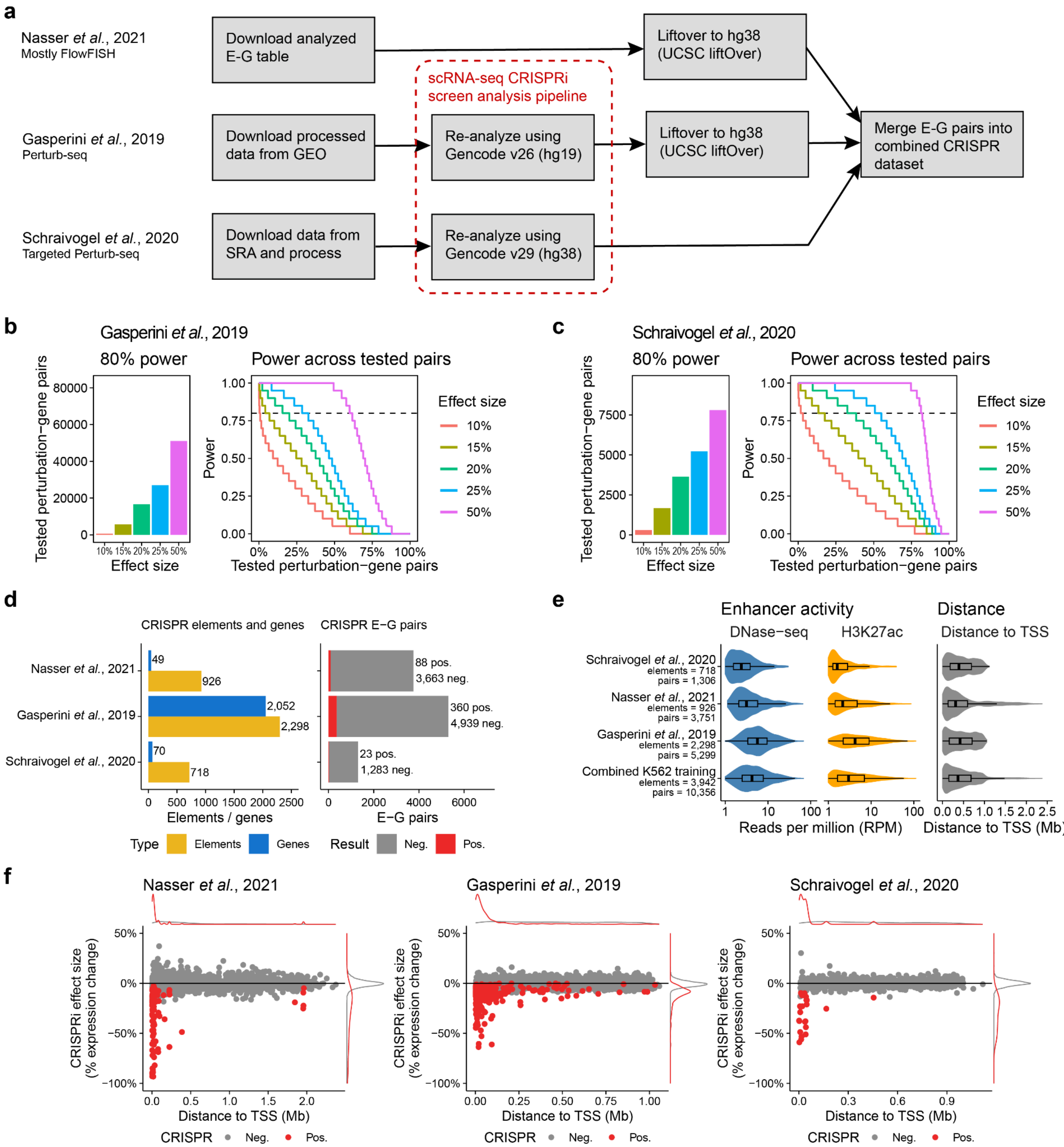

Supplementary Fig. N1.1 | Generation of the combined K562 training CRISPR dataset

a, Overview of approach to re-analyze and integrate three published CRISPRi screens from Nasser *et al.*, 2021[9], Gasperini *et al.*, 2019[3] and Schraivogel *et al.*, 2020[4].

b, Distribution of statistical power to detect specific effect sizes across all experimentally tested element-gene (E-G) pairs (n = 82,488) and number of E-G pairs with at least 80% power to detect specific effect sizes for the Gasperini *et al.*, 2019[3] dataset.

c, Distribution of statistical power to detect specific effect sizes across all experimentally tested element-gene (E-G) pairs (n = 9,492) and number of E-G pairs with at least 80% power to detect specific effect sizes for the Schraivogel *et al.*, 2020[4] dataset.

d, Number of tested elements and target genes, and number of CRISPR positive (significant negative effect on target gene expression) and negative E-G pairs per individual dataset in the combined K562 CRISPR dataset (**Methods**).
e, Properties of the combined K562 CRISPR dataset. Shown are activity metrics (DNase-seq and H3K27ac reads per million overlapping enhancers) for all tested elements and distance to TSS distributions for all tested E-G pairs. Data shown for the combined dataset and per individual input dataset. Box plot elements: center line: median; box bounds: 25th and 75th percentile (interquartile range); whiskers extend to most extreme value within 1.5 x interquartile range from the box; outlying points are not shown individually.
f, Observed CRISPR effect sizes as a function of distance to target gene TSS per for each individual dataset in the combined K562 CRISPR dataset.

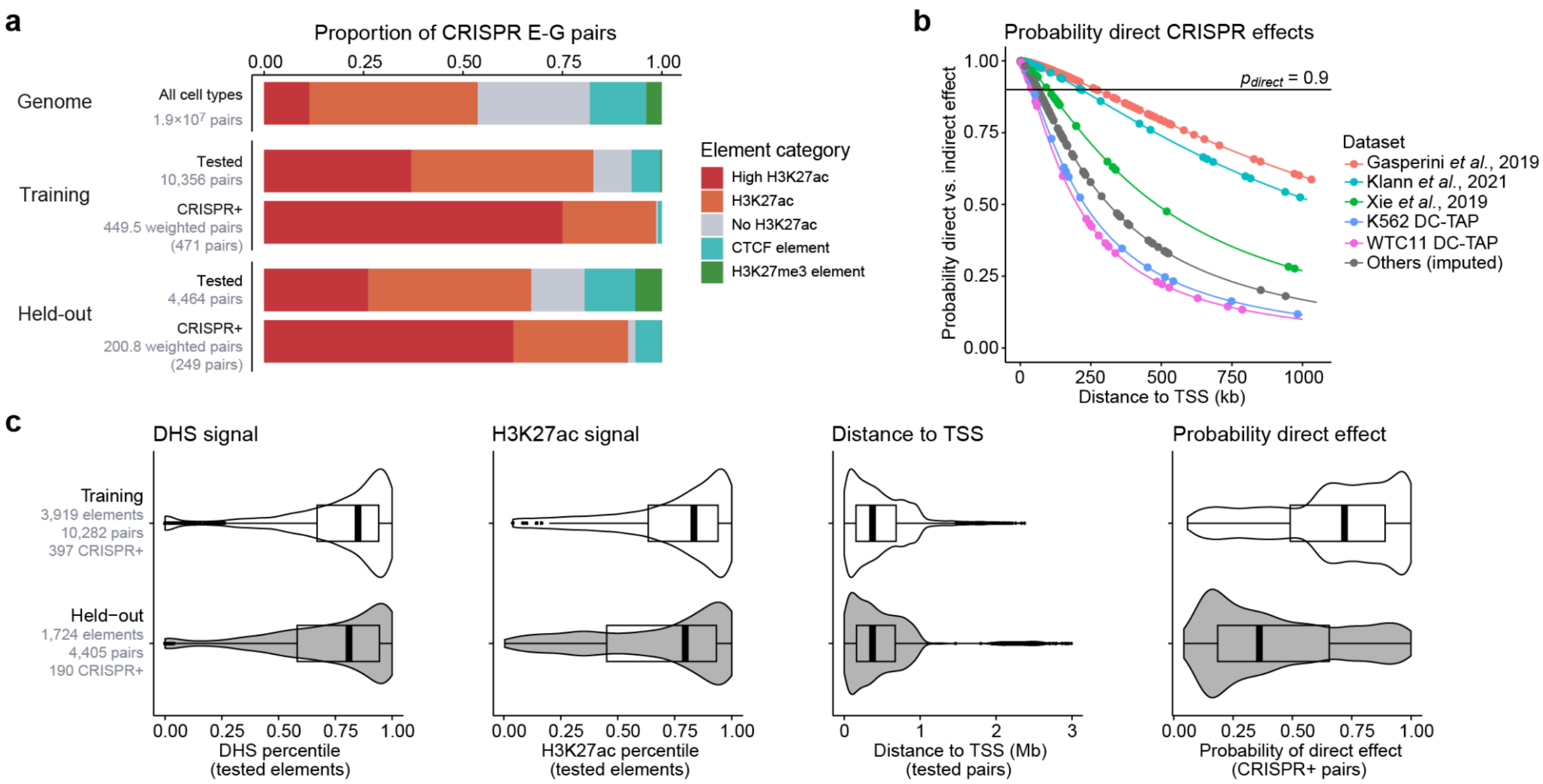

Supplementary Fig. N1.2 | Generation and analysis of the held-out CRISPR dataset

a, Proportions of element category proportions of CRISPR E-G pairs. Genome shows proportions of element categories for genome-wide candidate E-G pairs in K562, GM12878, HCT116, Jurkat, and WTC11 cells as reference. Other bars show element category portions for all tested and positive CRISPR E-G pairs in both the training and held-out datasets (**Supplementary Methods 20**). For positives, proportions are calculated for numbers weighted by the probability of being direct effects (**Supplementary Methods 21**).

b, Probability of CRISPR E-G pairs being the result of direct effects (**Supplementary Methods 21**) as function of distance to TSS (up to 1Mb). Lines show probability of being a direct effect across all tested pairs and dots indicate probability for CRISPR positive E-G pairs (*n* training = 471, *n* held-out = 249). Others (imputed) shows probabilities for datasets in training and held-out data for which no indirect effects rate was computed and instead was imputed by taking the average indirect effects rate across all datasets.

c, Properties of the filtered training and held-out CRISPR datasets (**Supplementary Methods 22**) showing distributions of DHS and H3K27ac signals at tested elements, distance of elements to TSS of tested pairs and probability of being direct *cis*-effects for CRISPR positive E-G pairs. Box plot elements: center line: median; box bounds: 25th and 75th percentile (interquartile range); whiskers extend to most extreme value within 1.5 x interquartile range from the box; dots: outlying data points beyond whiskers.

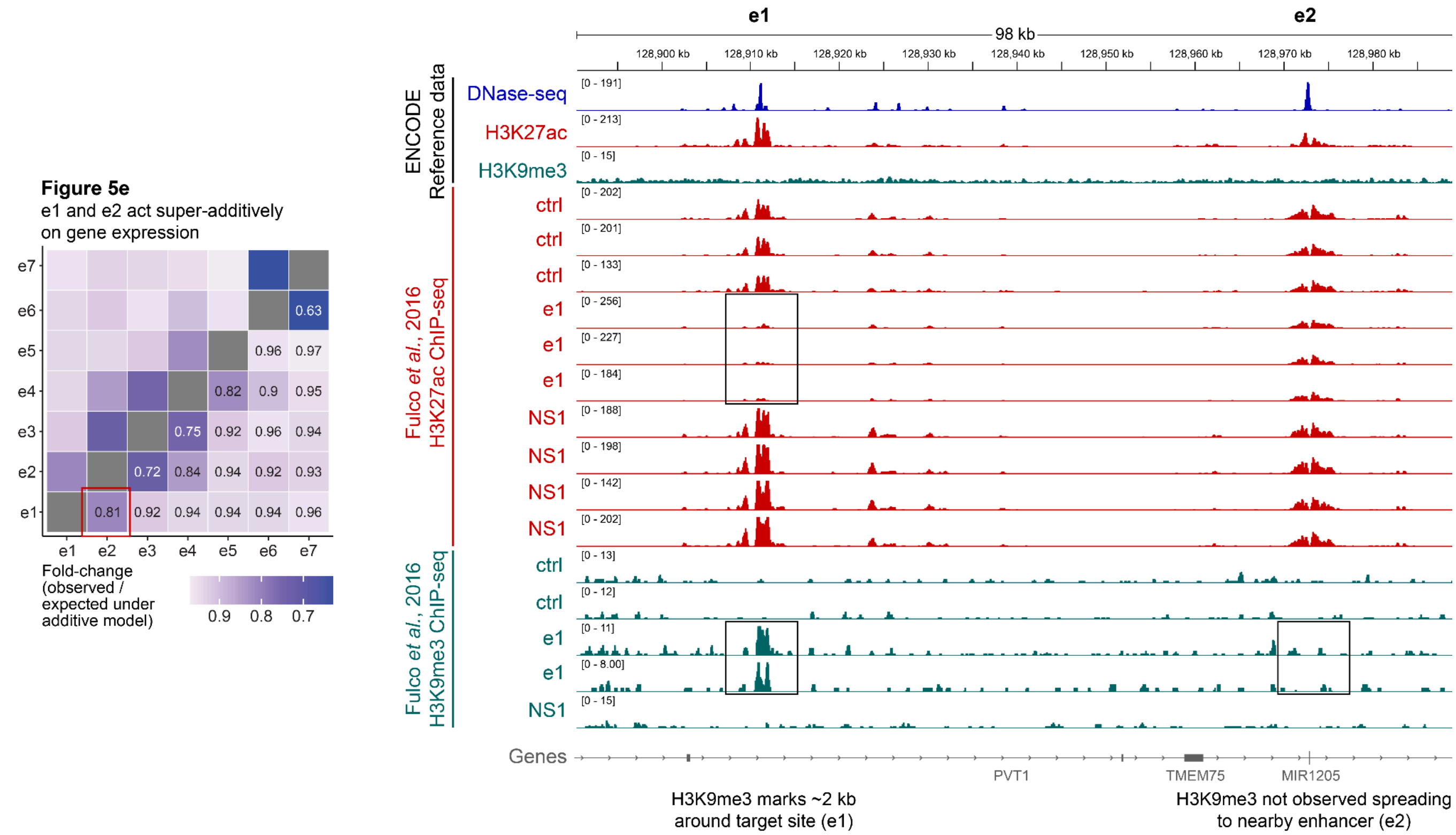

Supplementary Fig. N1.3 | Spreading of CRISPRi repressive signal

MYC enhancer e1 was repressed using dox-inducible CRISPRi dCas-KRAB for 24h. H3K27ac ChIP-seq shows successful repression of the activity of e1, the close-by *MYC* enhancer e2 remains active. H3K9me3 ChIP-seq shows no spreading of the repressive signal beyond 1-2kb from e1. Perturbation of another non-enhancer site (NS1) in the same region (not shown) does not affect activity of e1 or e2, and does not lead to spreading of repressive signal in the locus. Chromatin data was obtained from Fulco *et al.*, 2016[2] and the ENCODE portal[19].

Supplementary Note 2. Evaluating features of 3D genome organization

The regulatory functions of an enhancer on a target promoter are thought to be influenced by 3D genome organization, but the extent to which features of 3D contact maps can predict regulatory interactions has been debated[20]. To study such effects, the ENCODE Consortium has measured and defined various features of the 3D genome — including 3D loops (from Hi-C or ChIA-PET), 3D contact frequencies (from Hi-C), contact domains (from Hi-C), and CTCF occupancy (with ChIP-seq) (**Supplementary Table 2**). Here, we systematically assessed how individual features of 3D genome organization correlated with regulatory effects of enhancers on target genes and contributed to the accuracy of predictive models.

We first characterized the relationship of genomic distance with these features, as well as with measured and predicted regulatory enhancer-gene pairs (**Supplementary Fig. N2.1a-c**). As expected, each feature showed an inverse relationship with distance, with different distributions (**Supplementary Fig. N2.1a-c**). For example, the 3 types of measured regulatory enhancer-gene relationships each had different decreasing frequencies with distance (median distance = 16.1, 33.2, and 54.9 kb for eQTL, CRISPR, and GWAS enhancer-gene pairs, respectively; 87.1%, 74.1%, and 63.0% of regulatory pairs were located within 100 kb) (**Supplementary Fig. N2.1a**). The predictive models, including ENCODE-rE2G and ABC, showed similar relationships with distance (**Supplementary Fig. N2.1c**).

We next assessed the extent to which these features of 3D contact maps, either alone or in combination with other features, contributed to predicting regulatory interactions identified in CRISPR experiments:

Features from measurements of 3D physical interactions, each considered alone, showed poor prediction accuracy (**Supplementary Fig. N2.1d-e**). For example, assigning enhancers to promoters on the basis of quantitative 3D contact frequency performed better than binary loop or domain calls (recall = 48% at 70% precision, for cell-type specific ENCODE Hi-C at 2 billion reads), but only slightly better than genomic distance (recall = 32% at 70% precision, **Supplementary Fig. N2.1e**).

Strong 3D contact frequency was neither necessary nor sufficient for enhancer regulation (**Supplementary Fig. N2.1d**). Among the tested E-G pairs in the top 1% of contact frequencies, only 66 of 102 showed regulatory effects in the CRISPR experiments. The remaining 36 E-G pairs that did not affect gene expression had very low enhancer activity (as estimated from DNase-seq and H3K27ac signals), consistent with these elements being accessible but without strong activating functions (**Supplementary Fig. N2.1d**). Conversely, while 99% of the tested E-G pairs in the bottom decile of 3D contact frequencies did not have regulatory effects, 7 pairs did. These pairs tended to have very strong activity, consistent with a model where enhancer effects depend on both enhancer activity and 3D contact frequencies.

Accordingly, we next combined different estimates of 3D contact frequency with information about enhancer activity using the Activity-by-Contact framework[1], and found that models using contact frequencies from Hi-C outperformed models that used genomic distance alone. Specifically, we constructed ABC models in which we estimated 3D contact frequency with either: (i) cell-type specific ENCODE Hi-C data (K562, 2 billion reads) at 5 kb resolution, either coverage-normalized (scale-invariant, normalizing for 1D coverage) or unnormalized; (ii) CTCF or RNA Pol II ChIA-PET counts between the element and target gene promoter, or for loops spanning or crossing the E-G pair; (iii) a cell-type-average Hi-C Megamap, in which we averaged contact frequencies from ENCODE Hi-C datasets in 65 tissues to capture cell-type-invariant features of genome organization; or (iv) a power-law function of genomic distance fit to Hi-C data (1 / $\text{distance}^{1.03}$). All of these methods performed far better when combined with enhancer activity than they did alone (**Supplementary Fig. N2.1e-g**). Among these methods, ABC using cell-type-specific ENCODE Hi-C data (5-kb resolution, normalized) performed best (AUPRC = 0.601), cell-type-averaged ENCODE Hi-C (AUPRC = 0.565) and the power-law function of distance (AUPRC = 0.568) (**Supplementary Fig. N2.1f**). These

differences were especially pronounced for E-G pairs located >100 kb apart, where both cell-type specific and cell-type-averaged Hi-C measurements substantially outperformed the power-law (AUPRC = 0.151, 0.120, and 0.076, respectively; **Supplementary Fig. N2.1g**). Together, these observations confirm the importance of pairwise enhancer-promoter contact frequencies in predicting enhancer-promoter regulation, and show that cell-type-specific features of 3D contact maps contribute to long-range enhancer regulation beyond a simple function of genomic distance. Notably, cell-type-averaged ENCODE Hi-C explained 94% of the variance in the cell-type-specific map (at 5-kb resolution, **Supplementary Fig. N2.1k**), supporting the use of a generic map for making predictions in cell types in which Hi-C data are not yet available.

Finally, we found that combining pairwise E-G contact frequency with information about CTCF-bound loops that cross or span the enhancer-promoter pair improved regulatory predictions. We computed two quantitative scores based on CTCF ChIA-PET data: the PET counts spanning the E-G pair ("Spanning CTCF loop counts"), and the PET counts crossing the range of the E-G pair (where one PET anchor is inside the E-G pair, and one is outside; "Crossing CTCF loop counts") (**Supplementary Fig. N2.1h**). Regulatory E-G pairs from CRISPR experiments were 1.9-fold enriched for having at least one spanning CTCF loop count, compared to non-regulatory E-G pairs (P = 0.00000721, Fisher's exact test, **Supplementary Fig. N2.1i**), and tended to have lower crossing CTCF loop counts (odds ratio = 0.8, P = 0.000649, Fisher's exact test). We tested combining these features with the ABC score or with a set of baseline features in logistic regression models, and found that these features led to a modest but significant increase in performance (AUPRC = 0.601 vs 0.625 for ABC alone versus ABC plus spanning and crossing CTCF loop counts, $P_{bootstrap}$ = 0.008 (1,000 iterations); **Supplementary Fig. N2.1j**). In contrast to these CTCF looping features, incorporating CTCF binding strength or CTCF ChIA-PET counts directly at or between the enhancer or promoter did not improve performance in logistic regression models. Indeed, of the ~500 regulatory E-G pairs in the CRISPR dataset, only 88 (17.3%) had CTCF bound at or within 5 kb of the enhancer, only 49 (9.6%) had CTCF bound at or within 5 kb of the promoter. Together, these observations indicate that, while only a few regulatory enhancer-promoter pairs may be directly mediated by loops with CTCF anchors at the enhancer or promoter, information about CTCF loops spanning or crossing the E-G pair can inform enhancer-gene regulation beyond pairwise enhancer-promoter contact frequencies.

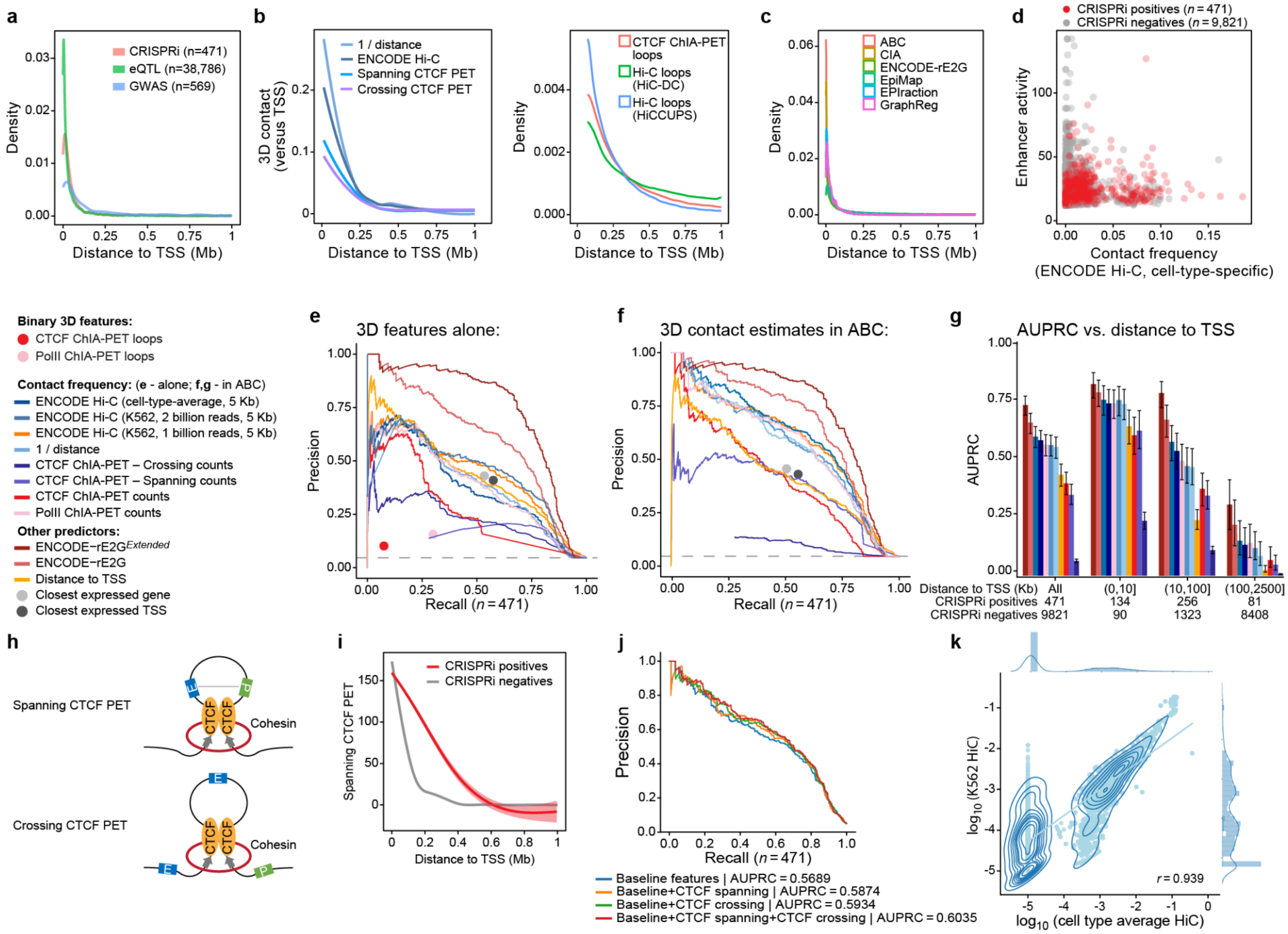

Supplementary Fig. N2.1 | Impact of 3D contacts on enhancer regulation

a, As a function of distance, frequency of E-G regulatory interactions from CRISPRi experiments (red), eVariant-eGene pairs (green), and known GWAS variant-gene pairs (blue).

b, As a function of distance, frequency of various 3D contact measurements.

c, As a function of distance, frequency of ABC and ENCODE-rE2G positive predictions.

d, Relationship between enhancer activity and 3D contact frequency for regulatory (red) and non-regulatory (gray) E-G pairs from the K562 CRISPRi dataset. X-axis: Enhancer-promoter contact frequency, from cell-type-specific Hi-C data (5-kb), normalized so that contact frequency at the promoter is 1. Y-axis: Enhancer activity as estimated by the ABC model (geometric mean of DNase-seq and H3K27ac ChIP-seq signals).

e, Precision-recall plot showing the performance of different 3D features at classifying E-G regulatory interactions from the combined K562 CRISPR dataset (*n* = 10,356 tested element-gene pairs, 471 positives). Curves: continuous predictors; dots: binary predictors; dashed line: baseline precision. Blue and purple curves represent different measurements or estimates of 3D contact frequencies used directly for prediction.

f, Similar to **e**, with blue and purple curves representing ABC models incorporating each 3D contact measurement.

g, AUPRC performance of ABC models using different estimates of 3D contact frequency for the combined K562 CRISPR dataset, binned by the distance from the element to the target gene TSS. Error bars: 95% bootstrap AUPRC range (10,000 iterations).

h, Illustrating two ways CTCF loop can impact enhancer-promoter (E-P) contacts: a CTCF loop containing E-P pairs facilitates their interaction; a CTCF loop crossing E-P pairs limits their interaction.

i, Spanning CTCF loop counts from CTCF ChIA-PET as a function of distance for CRISPRi positive (red, *n* = 471) and negative (gray, *n* = 9,821) E-G pairs.

j, Incorporating PET count of CTCF loops spanning and crossing the E-G pairs improves performance of logistic regression models on the combined K562 CRISPR dataset. “Baseline features” include activity, 3D contact, distance, and others (**Supplementary Methods 14**).
k, Scatterplot showing the relationship between K562 cell type specific Hi-C data and Average Hi-C data across 30 diploid cell types for all E-G pairs in the combined K562 CRISPR dataset. *r:* Pearson correlation coefficient.

Supplementary Note 3. Evaluating features of correlation between enhancer and promoter activity

Many previous approaches to link enhancers to their target genes have involved the assumption that the activities of enhancers and their regulatory target promoters should correlate across cell types or states[21–23]. Such correlations in enhancer and promoter activities depend on the compendium of cell types included in the analysis, and their utility in predicting regulatory interactions has not been systematically compared to other approaches. To test this, we computed 3 metrics: (i) a modified correlation between quantitative DNase-seq signals at enhancer-promoter pairs across 89 biosamples, in which we used generalized least squares to adjust the correlation for particular pair to account for the genome-wide covariance structure among cell types in the analysis ("GLS coefficient", **Supplementary Methods 7**); (ii) a standard uncorrected DNase-DNase Pearson correlation across the same 89 biosamples; and (iii) a "DNase-RNA GLS coefficient", in which we conducted a similar analysis correlating DNase-seq signals at a distal enhancer with RNA expression level of a nearby gene across 96 matched PolyA+ RNA-seq and DNase-seq datasets.

Overall, the GLS coefficient varied with genomic distance (**Supplementary Fig. N3.1a**), with higher values at shorter distances similar to the distance distributions of CRISPR-validated enhancers and eQTLs (**Supplementary Fig. N3.1b**). We compared GLS coefficients to experimental data on enhancer-gene regulatory interactions, and found that regulatory pairs in CRISPR experiments indeed showed a significantly higher GLS coefficient than distance-matched non-regulatory pairs (mean = 0.3209 vs. 0.1311, respectively; two-sided Wilcoxon rank-sum test $P = 3.28 \times 10^{-14}$; **Supplementary Fig. N3.1c**). However, we did not observe similar results for fine-mapped eQTL variants. We overlapped GTEx lymphoblastoid eQTLs with active enhancers in GM12878 cells and compared them with non-significant eQTLs. The GLS coefficient, on average, was not significantly lower for GM12878 enhancer-promoter pairs supported by GTEx eQTLs than the ones overlapping the distance-matched non-significant eQTLs (mean = 0.2899 vs. 0.3447, respectively; two-sided Wilcoxon rank-sum test P = 0.2141; **Supplementary Fig. N3.1d**). Furthermore, the distributions of GLS coefficients for both CRISPR enhancers and eQTL variants were highly overlapping with their respective controls (**Supplementary Fig. N3.1b-d**). As such, the GLS coefficient alone was a poor predictor of regulatory interactions in the CRISPR dataset (AUPRC = 0.174; **Supplementary Fig. N3.1e**). The two other correlation-based metrics showed even worse performance (AUPRC = 0.103 and 0.112 for Pearson correlation and RNA GLS coefficients, respectively).

We tested whether the correlations in enhancer-promoter activity across cell types might have independent predictive information from cell-type specific measurements of enhancer-promoter activities or 3D contact frequencies. To do so, we trained logistic regression models in which we supplemented the ABC model or other e feature sets with the GLS coefficient, and found that inclusion of the GLS coefficient led to a modest improvement in predictive performance (**Supplementary Fig. N3.1e**). However, excluding the GLS coefficient from the ENCODE-rE2G^{Extended} model did not significantly reduce performance (**Supplementary Fig. N3.1e, Supplementary Fig. N6.2b**).

Inspection of the variation of enhancers and promoters across cell types provided a simple explanation for this limited predictive power. We separated the genes from the CRISPR dataset into two groups: the genes expressed in all 89 biosamples (broadly active promoter) and the ones expressed in several samples (variably active promoters). Similarly, we separated enhancers into 3 groups based on their pattern of activity across cell types: broadly active, specifically active in a few tissues (1-4), and inactive (**Supplementary Fig. N3.1f**). Genes paired with either broadly active or specifically active enhancers showed similar distributions of enhancer-promoter distances (**Supplementary Fig. N3.1g**). In cases where both the enhancer and promoter were active only in specific cell types, the GLS coefficient performed well. In most cases, however, promoters are active in a much wider array of cell types than

the enhancer is active, leading to reduced correlation and worse predictive power (**Supplementary Fig. N3.1h-k**).

In summary, correlations in enhancer and promoter activity across cell types were insufficient to accurately identify enhancer-gene regulatory interactions, and had only modest/no independent predictive power that could not be captured by features derived from the single specific cell type of interest.

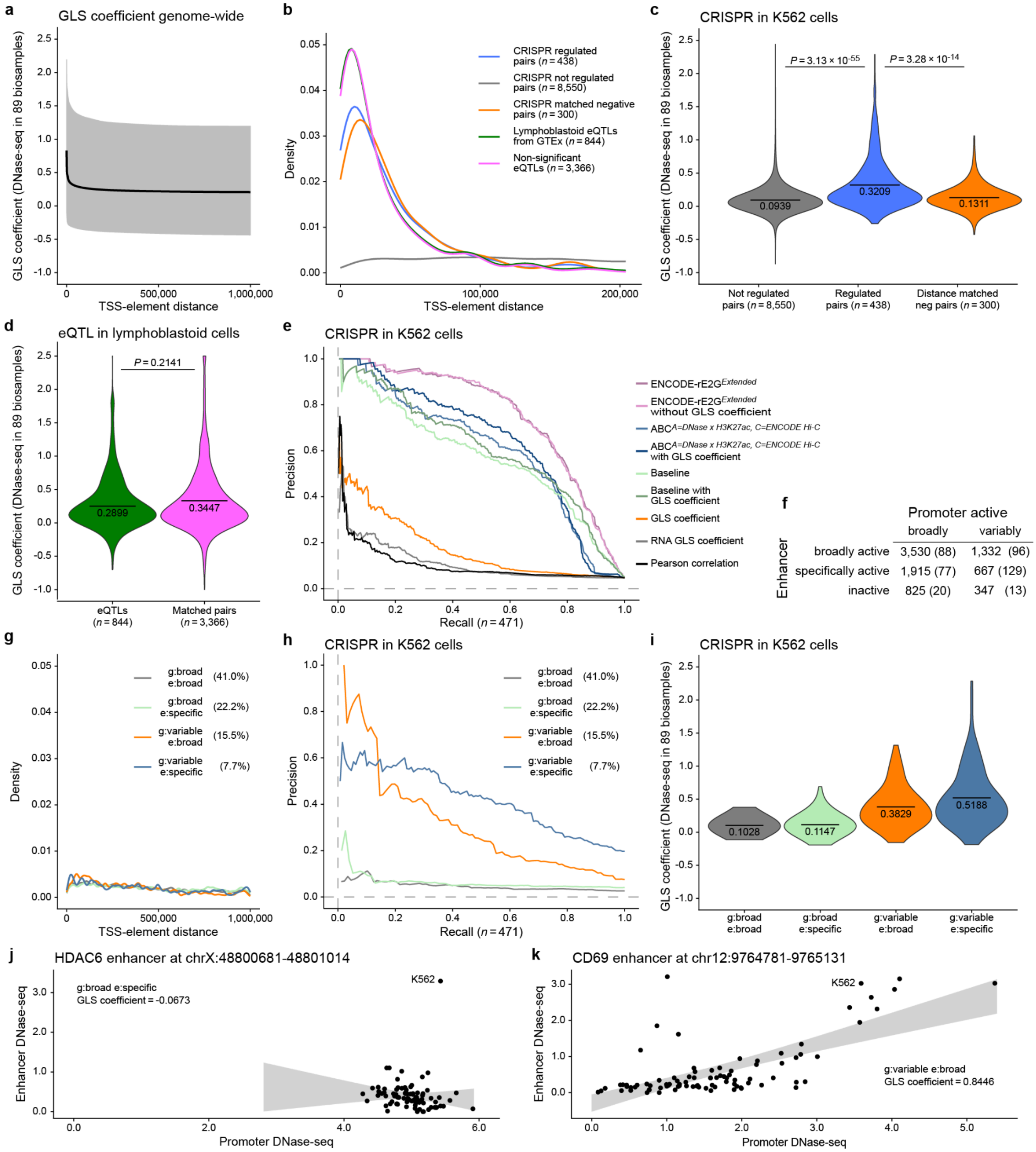

Supplementary Fig. N3.1 | Informativeness of correlations in enhancer-promoter activity across cell types

a, Distance distribution of GLS coefficient, 3.7 million observations were sorted by distance, mean and 95% intervals across 10,000 consecutive observations are shown.

b, Distance distribution of K562 CRISPR pairs and GTEx eQTLs.

c, GLS coefficient in K562 CRISPR positive and negative enhancer-promoter pairs. Horizontal lines indicate mean values.
d, GLS coefficient for enhancer-promoter pairs supported by GTEx eQTLs from lymphoblastoid cells. Horizontal lines indicate mean values.
e, Precision-recall curves for GLS coefficient at predicting CRISPR positive interactions in the combined K562 CRISPR dataset and its contribution to other models.
f, Classification of CRISPR pairs into different categories based on the amount of cell types corresponding promoter and enhancer are active in. The numbers in parentheses correspond to CRISPR positive pairs.
g, No substantial difference in distance distribution for enhancer-promoter pairs with different spectra of activity.
h, Precision-recall curves for GLS coefficient at predicting CRISPR positive interactions in the combined K562 for different groups of enhancer and promoter activities.
i, GLS coefficient distribution in corresponding groups. Horizontal lines indicate mean values.
j, Example of the broadly expressed gene *HDAC6* regulated by K562-specific enhancer in K562 cells. Biosamples with no active enhancer dominate for GLS coefficient calculation, resulting in non-informative random estimation.
k, Example of the gene *CD69* expressed in several biosamples and regulated by broadly active enhancers. GLS coefficient is able to efficiently predict such interactions.

Supplementary Note 4. Evaluating differences between ubiquitously and specifically expressed genes

Recent studies suggest that enhancer-promoter regulation in the genome may be influenced by differences in the architecture of different promoters, and in particular that ubiquitously expressed (*e.g.*, housekeeping) genes might be less sensitive to distal enhancers[24,25]. We have evidence to support this effect across our dataset:

We first classified genes via three metrics: (1) The enhancer correlation score - in which we give genes a score based on how consistent their enhancers are across cell types, as calculated by correlating the ABC scores of enhancers across our previous atlas[9]. (2) Promoter class - in which, as previously defined based on ExP-STARR-seq assays[24], “P1” promoters have weaker intrinsic activity and are more strongly regulated by enhancers and “P2” promoters are intrinsically stronger, less sensitive to enhancers and more ubiquitously expressed across cell types. (3) Expression ubiquity - in which we stratify genes based on the ubiquitousness and uniformity of their expression across cell types (**Supplementary Methods 6.6**).

We found that for each of these three metrics, genes in the more constitutive gene categories (those with enhancer correlation scores in the top 25% of genes, genes predicted to have P2 promoters, and ubiquitously and uniformly expressed genes) were less likely to be linked to eQTLs in predicted enhancers (**Supplementary Fig. N4.1a**) and predicted enhancers for these genes were less likely to be true regulators in CRISPRi datasets (**Supplementary Fig. N4.1b**).

We tested whether gene class could be used to improve our predictive models. We found that when we add each of these features to a logistic regression model with just the ABC score, all three lead to significant increases in the AUPRC relative to ABC score alone (**Supplementary Fig. N4.1c-d**). Promoter class and expression uniformity in particular significantly improve the precision of the models (**Supplementary Fig. N4.1e**) and reduce the number of false positive predictions (**Supplementary Fig. N4.1f**). Furthermore, expression ubiquitous (captured in the feature “ubiquitous expression”) and promoter class are both shown to be informative in our sequential feature selection analysis: they ranked 2nd and 8th out of 45 features in the ENCODE-rE2G^{Extended} model (**Supplementary Fig. N6.3b**) and ubiquitous expression ranked 2nd out of 12 features in the ENCODE-rE2G model (**Supplementary Fig. N6.3a**). Thus, incorporating information about promoter class improves predictions of enhancer-gene regulation by adding information orthogonal to that captured by the ABC score.

Together, these observations are consistent with a model in which sequence-intrinsic features of the promoters of ubiquitously expressed genes make them insensitive to distal enhancers.

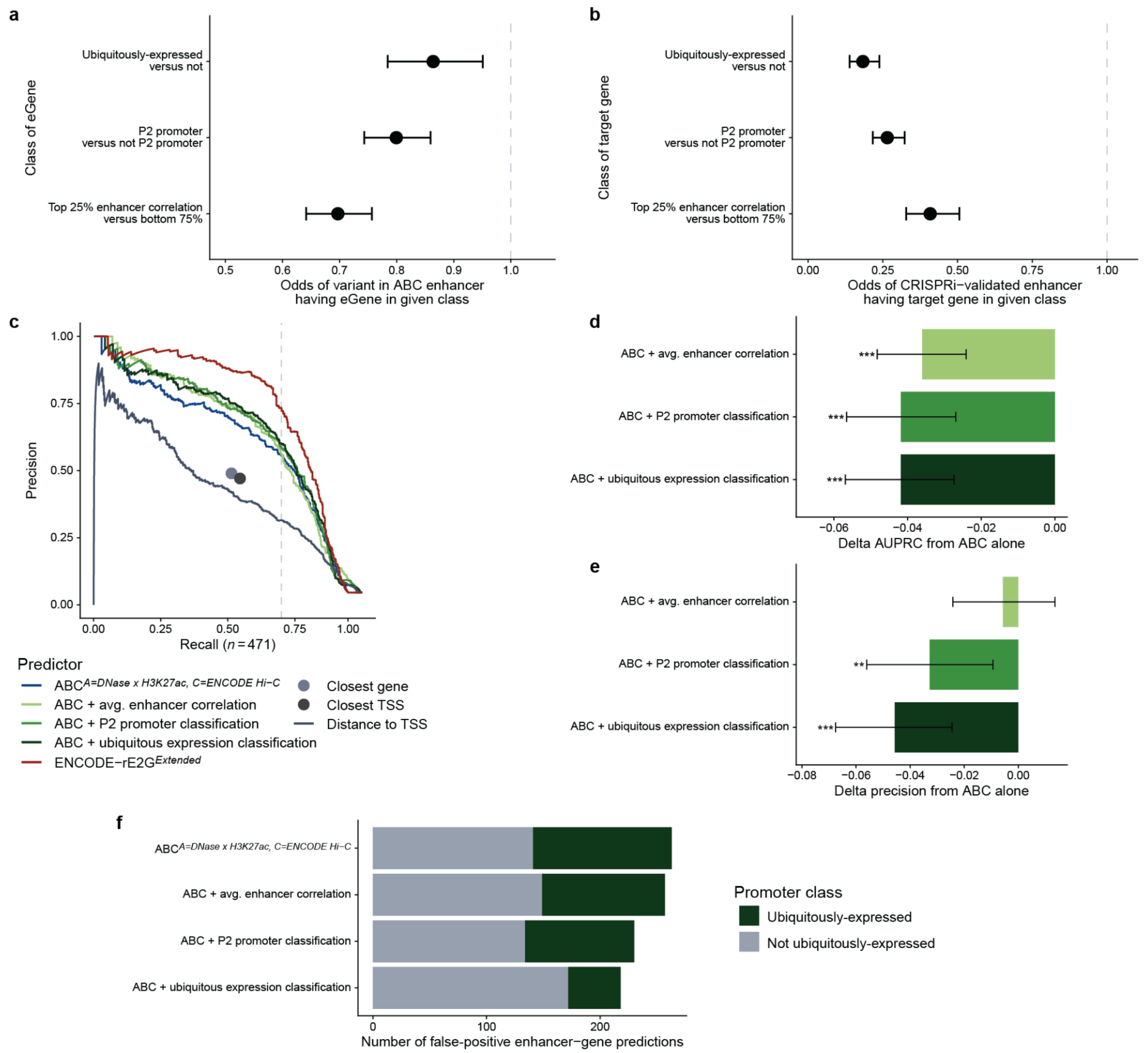

Supplementary Fig. N4.1 | Differences in distal regulation between gene/promoter classes

a, Odds of fine-mapped eQTL located in a predicted ABC enhancer having eGenes in each of the three classes. Ubiquitously-expressed genes, P2 promoter class, and genes with enhancer correlation scores in the top 25% of scores are respectively 86.4% ($P = 2.56 \times 10^{-3}$, Fisher's Exact Test), 80.2% ($P = 8.33 \times 10^{-10}$, Fisher's Exact Test), 69.7% ($P = 1.08 \times 10^{-18}$, Fisher's Exact Test) as likely as their counterparts to have an eQTL for that gene in a predicted ABC enhancer. The error bars denote 95% confidence intervals. Here, predicted ABC enhancers are from our previous atlas of predictions across 131 cell types[9]. The eQTLs considered had a fine-mapping PIP > 0.5, were located in distal noncoding regions of the genome, and were from any of 32 tissues from the GTEx V8 resource[26], the same as was used for the eQTL benchmark described in **Supplementary Methods 24**. Overlap of eQTLs and predicted enhancers was agnostic to eQTL biosample and prediction cell type.

b, Odds of a CRISPRi-validated enhancer-gene link gene having its target gene in each of three classes. Ubiquitously-expressed genes, P2 promoter class, and genes with enhancer correlation scores in the top 25% of scores are respectively 18.3% ($P = 3.83 \times 10^{-51}$, Fisher's Exact Test) , 26.5% ($P = 3.17 \times 10^{-43}$, Fisher's Exact Test), 40.9% ($P = 2.27 \times 10^{-18}$, Fisher's Exact Test) as likely as their counterparts to have an CRISPRi-validated enhancer targeting that gene. The error bars denote 95% confidence intervals.

c, Precision-recall curves showing performance of logistic regression models comprising ABC and one of the three gene class features on the combined K562 CRISPR dataset. Curves: continuous predictors; dots: binary predictors; dashed line: 70% recall.

d, Delta AUPRC on the CRISPR benchmark between ABC alone and the logistic regression models combining ABC with a gene class feature. Error bars represent 95% range of delta AUPRC values inferred via bootstrap (10,000 iterations). For each delta AUPRC shown, $P_{bootstrap} = 1 \times 10^{-4}$ (two-sided, 10,000 iterations); the three stars indicate $P_{bootstrap} < 0.001$.

e, Delta precision at 70% recall on the ABC benchmark between ABC alone and the logistic regression models combining ABC with a gene class feature. Error bars represent 95% range of delta precision values inferred via bootstrap (10,000 iterations). The $P_{bootstrap}$ values (10,000 iterations) for delta precision between ABC and the addition of enhancer correlation, P2 promoter class, and ubiquitous expression features are 0.56, 4.8×10^{-3}, and 1×10^{-4}, respectively. Two stars indicate $P_{bootstrap} < 0.01$; three stars indicate $P_{bootstrap} < 0.001$.

f, Number of false positive enhancer-gene predictions on the CRISPR benchmark for ABC alone compared to ABC with each of the gene class features, stratified by whether the target gene is classified as ubiquitously-expressed.

Supplementary Note 5. Using ENCODE-rE2G maps to interpret GWAS signals

We assessed how to interpret ENCODE-rE2G predictions when applied to link noncoding variants associated with common, complex diseases to target genes and cell types. To this end, we analyzed fine-mapped distal noncoding variants from GWAS studies for 94 traits from the UK Biobank (PIP > 0.1, n = 29,245) and expression QTLs for 49 tissues from GTEx (PIP > 0.1 for >=1 gene, n = 247,049) (**Supplementary Table 6, Data Availability**). To assess enrichment of these variants in enhancers identified by ENCODE-rE2G, we compared these fine-mapped variants to a control set: all distal noncoding variants detected in the 1000 Genomes project (n = 9.2 million).

In total, we identified that 56% of fine-mapped GWAS variants and 51% of eQTL variants overlapped an ENCODE-rE2G enhancer in one or more of the 1,458 biosamples, compared to only 33% of control variants (1.7 and 1.5-fold enrichment, respectively) (**Supplementary Fig. N5.1a**). In comparison, repeating this for all DNase peaks, 74% of GWAS variants and 69% of eQTL variants overlapped with DNase across all biosamples, compared to 55% of control variants (1.3 and 1.2-fold enrichment, respectively). Blood-related traits showed a 11% higher fraction of overlap with ENCODE-rE2G predictions than an average trait, while brain-related traits showed 31% lower fraction of overlap with ENCODE-rE2G predictions (**Supplementary Fig. N5.1b**). This can be attributed to both (i) poor representation of brain-related cell types compared to immune cell types among the ENCODE-rE2G biosamples, and (ii) high polygenicity of brain-related traits[27]. Our GWAS analyses expectedly showed that variants were enriched for overlapping ENCODE-rE2G enhancers in expected tissues (**Fig. 3d, Supplementary Table 7**). However, in some cases, we also observed significant enrichment for variants for a given trait in enhancers in other biosamples not likely to be relevant, presumably due to sharing of enhancers across multiple cell types or tissues. For any given variant that overlapped an enhancer in 1 biosample, that same variant overlapped enhancers in an average of 63 other biosamples providing multiple candidate biosamples in which the variant might act. At the same time, 22% of variants showed strong cell-state specificity, in that they were identified in only one biosample, often excluding other related biosamples.

Next, towards guiding the design of future data collection, we considered the marginal value of adding biosamples to the compendium. For each trait, we examined the fraction of prioritized fine-mapped variants that were uniquely captured by biosample with the greatest genome-wide enrichment for that trait — *i.e.*, the fraction of variants that would have been missed if one did not have this biosample in the dataset. For 11 traits, we observed at least one fine-mapped variant that is nominated by ENCODE-rE2G only in the top enriched biosample. For example, T2D adjusted for BMI, HepG2 was observed as the top globally enriched biosample, and 25% of fine-mapped variants validated by ENCODE-rE2G in HepG2 were unique to this biosample. We observed the proportion of fine-mapped variants for different traits supported by ENCODE-rE2G in (i) top 10 globally enriched biosamples and (ii) all biosamples (**Supplementary Fig. N5.1c**). We conclude that putatively disease-critical cell types tend to show the highest global enrichment of fine-mapped variants while for the disease, a large fraction of these fine-mapped variants are mapped by ENCODE-rE2G in other likely related biosamples; as a result, adding cell types closely related to biosamples already included in this ENCODE-rE2G resource may yield only limited additional discoveries with respect to predicting the target gene of noncoding variants. We examined the degree of redundancy in the ENCODE-rE2G variant-to-gene linking predictions across biosamples by focusing our analyses to a subset of top globally enriched biosamples for a trait. Genes linked to GWAS fine-mapped variants for a trait in the top 10 enriched biosamples showed on average 1.64-fold higher PoPS prioritization score for the trait compared to those linked in all biosamples. Of these, 64 of 94 traits showed significantly higher (FDR < 10%) average PoPS prioritization across linked genes between top 10 and all biosamples (**Supplementary Fig. N5.1c**).

As a further validation of biosample-specificity of traits, we examined the extent of overlap of GTEx eQTLs for 28 tissues in ENCODE-rE2G maps for 28 groups of biosamples matched to GTEx tissues (**Supplementary Table 7**). For 43% tissues, the GTEx eQTLs in a tissue showed the highest enrichment in ENCODE-rE2G for the matched set of biosamples; for 68% cases, the matched set of biosamples were among the top 3 most enriched (**Supplementary Fig. N5.1d**). For example, we observed the highest enrichment of fine-mapped eQTLs from GTEx cerebellum and cerebellar hemisphere in ENCODE-rE2G predictions of cerebellum-related biosamples (**Supplementary Fig. N5.1d**). These results highlight that using ENCODE-rE2G maps to identify candidate causal cell types for variants causally associated with diseases and molecular phenotypes should consider which biosamples are globally enriched for disease variants and consider sharing of enhancers across related cell types. We also expect that identification of causal cell types will likely benefit from additional information about the cell-type specificity of the effects of the variant on enhancer activity[28,29], which are not captured by ENCODE-rE2G.

We present 3 example GWAS causal variants for mean corpuscular hemoglobin (MCH) that are linked target genes by ENCODE-rE2G, in potentially causal cell types (**Supplementary Fig. N5.2**). The GWAS causal variant *rs4927708* for MCH (PIP = 1) is linked by ENCODE-rE2G to the gene *TFRC,* specifically in hematopoietic multipotent progenitor cells. The gene *TFRC* is known to play an important role in iron import and metabolism, and in erythroblast development[30,31]. *TFRC* is not only the top PoPS prioritized gene in the locus, but also is a top PoPs prioritized gene for MCH genomewide (rank 27). The variant *rs218265* (PIP = 1) is linked to the KIT gene in both K562 and hematopoietic progenitors. KIT is a proto-oncogene that has been linked to anemia and erythroid differentiation[32,33]. For the intronic GWAS causal variant, *rs7599488* (PIP = 0.89), we see an ENCODE-rE2G link to the overlapping gene, *BCL11A,* in hematopoietic multipotent progenitor cells but not in K562. This variant is located in a well-known clinically severe intronic enhancer for *BCL11A* that possesses erythroid-restricted, adult-stage-specific enhancer activity[34,35].

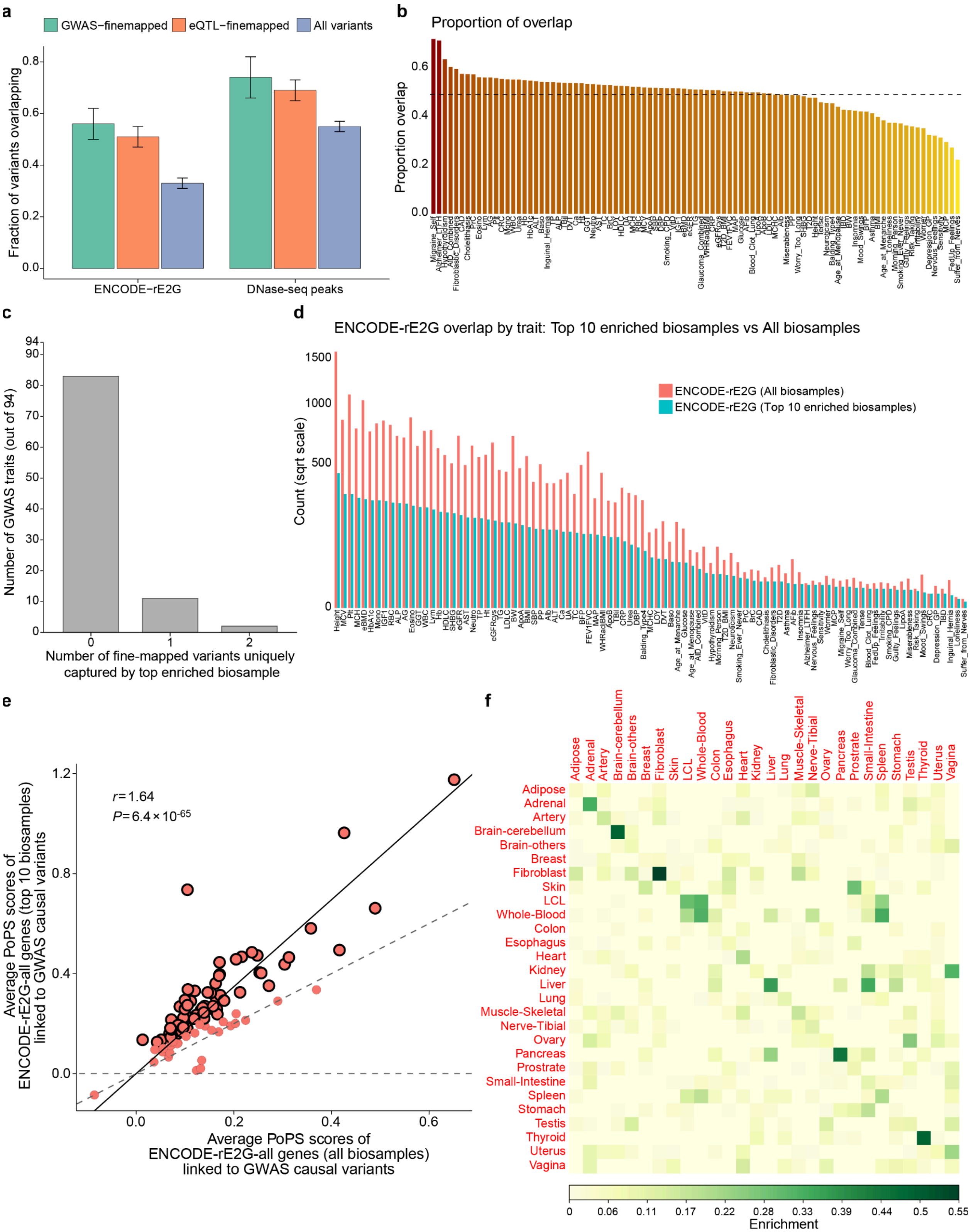

Supplementary Fig. N5.1 | Linking common variants fine-mapped from GWAS data by ENCODE-rE2G.

a, Fraction of (i) GWAS fine-mapped variants (PIP > 0.10) for one of 94 GWAS traits, (ii) eQTL fine-mapped variants (PIP > 0.50) in one of 49 GTEx tissues, and (iii) all common and low-frequency variants, that overlap (i)

ENCODE-rE2G predictions in at least one of 1,458 biosamples, or (ii) DNase-seq peaks in at least one of these biosamples. Error bars: 95% confidence intervals based on Jack-knife standard errors over chromosome blocks.
b, Fraction of GWAS fine-mapped variants (PIP > 0.10) that overlap an ENCODE-E2G gene-linked enhancer in one of the 1,458 biosamples. The traits are ordered based on the magnitude of this fraction.
c, Barplot of the number of GWAS traits corresponding to the number of unique fine-mapped GWAS (PIP > 0.1) variants specifically captured by ENCODE-E2G gene-linked enhancer in the top globally enriched biosample for the focal trait.
d, Barplot representing the number GWAS fine-mapped variants implicated by enhancer-gene links in (i) top 10 globally enriched biosamples in disease variants, and (ii) all biosamples, for each of the GWAS traits. We order the traits based on the highest number of represented fine-mapped variants in predicted enhancers.
e, Comparison of the average PoPS scores of genes linked to GWAS fine-mapped variants for traits by ENCODE-rE2G predictions in top 10 globally enriched biosamples based on Panel b (Y-axis) and ENCODE-rE2G predictions in all 1,458 biosamples (X-axis). Results are reported for 94 UKBiobank traits. Circled dots denote traits with significant (FDR < 10%) difference between these averages, the solid line denotes y=x, and the dashed line denotes the regression slope. *r*: slope of the regression line; *P: P*-value for the regression slope.
f, Heatmap representing the enrichment of GTEx fine-mapped eQTLs (maximum PIP across genes > 0.10) for 28 GTEx tissues in ENCODE-rE2G predictions in sets of biosamples that can be matched to each of these GTEx tissues. For a sparse representation, the enrichments are mean-adjusted across rows (GTEx tissues) and then across columns (ENCODE-rE2G biosample groups). All adjusted enrichments are thresholded below at 0, and are colored from white to green based on increasing magnitude of adjusted enrichment. Numerical results are reported in **Supplementary Table 7**.

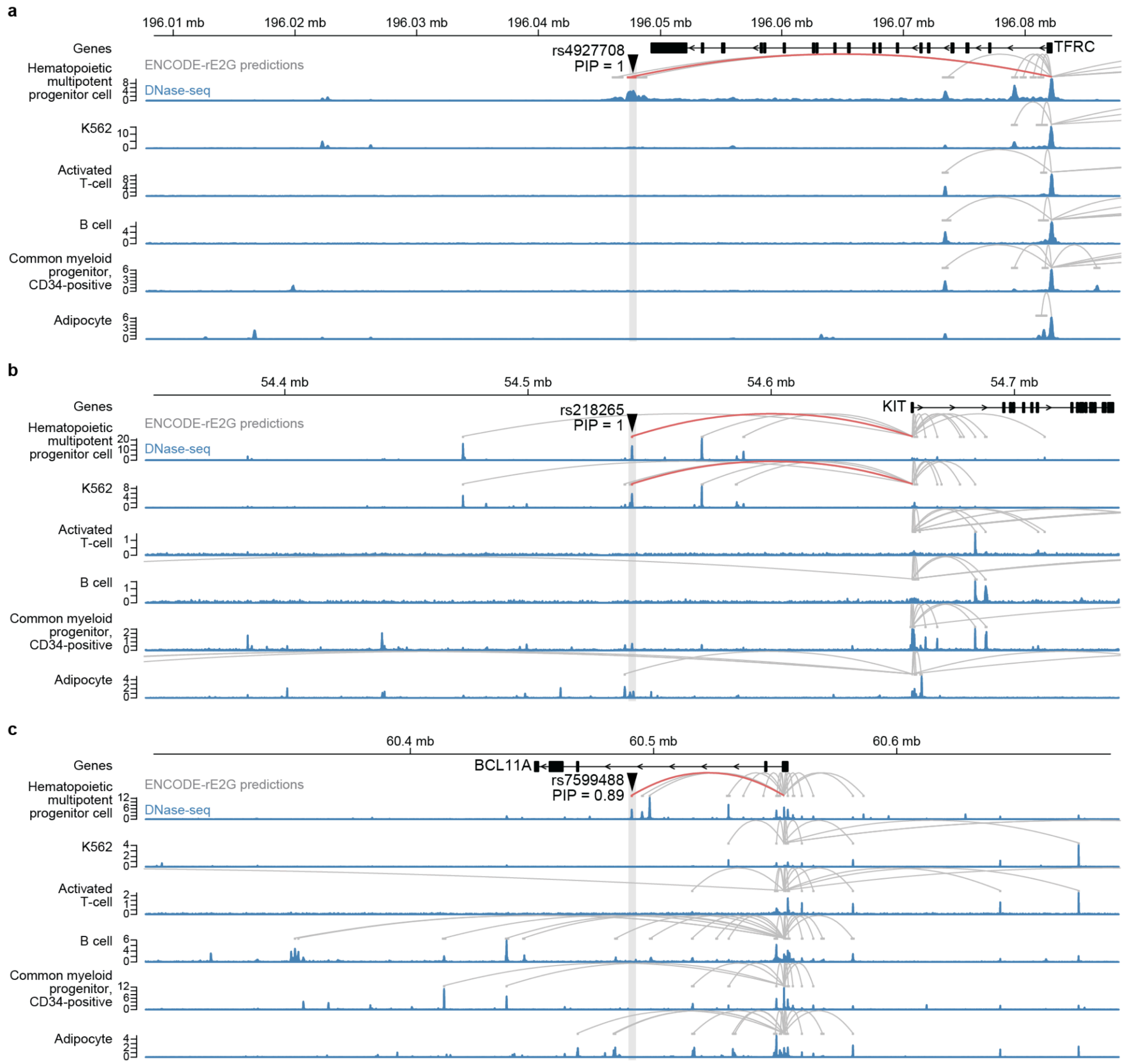

Supplementary Fig. N5.2 | Example GWAS loci linked to target genes by the ENCODE-rE2G ∩ PoPS

ENCODE-rE2G ∩ PoPS links two confidently annotated GWAS causal variants (PIP > 0.7) for mean corpuscular hemoglobin to target genes: **a** *rs4927708* to target gene *TFRC* in hematopoietic multipotent progenitor cells, **b** rs218265 to target gene KIT in hematopoietic progenitors and K562 and **c** intronic variant *rs7599488* to BCL11A gene specifically linked in hematopoietic progenitors.

Supplementary Note 6. Feature selection and importance in ENCODE-rE2G models

Feature selection:

To identify a minimal, non-redundant set of features for the model, we performed a “best subset” feature analysis by comparing the CRISPR benchmark performance of models trained on all possible sets of the twelve input features for ENCODE-rE2G, including several polynomial terms to account for potential nonlinear behavior. This approach to feature selection was chosen over L1 or L2 penalties to preserve the calibration of model scores to probabilities[36,37]. We found that the model could attain optimal performance (AUPRC greater than the 12 feature model) with over 30 feature sets, and minimally with 8 features (**Supplementary Fig. N6.1**). We chose one of the several 8-feature models (**Fig. 4a**) achieving optimal performance after evaluating the following additional feature analyses (**Supplementary Figs. N6.2a**, **N6.3a**, **N6.4a**) and considering known biological mechanisms of enhancer-promoter regulation.

We used a similar approach to select features for ENCODE-rE2G models built from other assay combinations (**Fig. 4f,g**). We also prioritized selecting analogous sets of features across models when supported by the “best subset” analysis (for example, using DNase signal at promoter in DNase-based models and ATAC signal at promoter in ATAC-based models).

Additional feature analyses:

We used several analysis strategies to evaluate the importance of features in the ENCODE-rE2G and ENCODE-rE2G^{Extended} models.

Because many individual features are correlated with one another, we performed a groupwise ablation analysis of the ENCODE-rE2G and ENCODE-rE2G^{Extended} models. To this end, we defined categories of related features (some features are assigned to multiple categories) and computed logistic regression models leaving out each category of features. We calculated the difference between the performance (AUPRC and precision at a recall of 70%) of the ablated model and the full-featured model to define the delta performance ($Delta_{AUPRC} = AUPRC_{ablated} - AUPRC_{full}$, and $Delta_{Precision} = Precision_{ablated} - Precision_{full}$). To assess the significance of differences in performance, we performed bootstrap sampling of the E-G pairs (1000 samples, with replacement). In order to see the effects of each feature group as a function of E-G distance, we also performed similar analyses after stratifying E-G pairs to three distance ranges [0, 10kb), [10kb, 100kb), and [100kb, 2.5Mb] (**Supplementary Fig. N6.2**).

For both ENCODE-rE2G and ENCODE-rE2G^{Extended}, the two most important categories were 3D contact & distance and enhancer activity (**Supplementary Fig. N6.2**) — as expected based on the high performance of previous models like ABC, which uses only these two components. Notably, ablation of the “contact” category, which removes all features derived from Hi-C and ChIA-PET but preserves features related to 1D genomic distance, significantly reduced performance. This supports observations that 3D contact measurements include information about enhancer-promoter regulation that is not captured by genomic distance alone (see also **Supplementary Note 2**). Interestingly, other categories of features beyond those included in ABC had smaller but significant effects on model performance, including promoter class and nearby enhancer activity (see also **Supplementary Note 4**, **Fig. 4**, **Fig. 5**).

The importance of feature categories varied across bins of genomic distances in the ablation analysis (**Supplementary Fig. N6.2a,b**). For example, while ablation of the “enhancer activity” feature category significantly affects model performance at all distance ranges, categories of features involving 3D contacts were only important in the longer distances ranges (>10 kb), as expected.

Next, we performed forward sequential feature selection on both models by adding the feature that leads to the largest increase in AUPRC (**Fig. 4c, Supplementary Fig. N6.3a**). For models using the 12 features from ENCODE-rE2G, AUPRC significantly increased when adding 7 features, although it achieved the highest AUPRC using 9 features including all features (**Fig. 4c, Supplementary Fig. N6.3a**). The first three features selected were the ABC$^{A=DNase,\ C=Average\ ENCODE\ Hi\text{-}C}$ score, whether the regulated gene was ubiquitously expressed, and the number of transcription start sites between the enhancer and promoter, consistent with the importance of these features in other analyses (**Fig. 4c-e**). For ENCODE-rE2G^{Extended}, only the addition of the first 7 of 45 features significantly improved AUPRC (**Supplementary Fig. N6.3b**). These 7 features included the ABC score, promoter class, enhancer and promoter activity, PET counts, and whether the enhancer and promoter are in the same topologically associating domain.

Finally, we performed a feature attribution analysis by computing SHAP scores for all used features (**Supplementary Fig. N6.4**). For ENCODE-rE2G, the top contributing feature is squared average Hi-C contact between the enhancer and promoter, and other top features include the DNase-seq signal at the enhancer (**Supplementary Fig. N6.4a**). For ENCODE-rE2G^{Extended}, the top feature is enhancer activity measured by DNase-seq and H3K27ac ChIP-seq. The top features contributing to performance of ENCODE-rE2G^{Extended} include those from diverse categories, including components of the ABC score, enhancer and promoter activity measured by DNase-seq and H3K27ac ChIP-seq, Hi-C contact frequency between the enhancer and promoter, EP300 ChIP-seq signal at the enhancer, and genomic features (e.g. the number of transcription start sites between the enhancer and promoter, number and activity of nearby elements) (**Supplementary Fig. N6.4b**).

Altogether, these results show that multiple categories of features are complementary and important for optimal performance of both the ENCODE-rE2G and ENCODE-rE2G^{Extended} models.

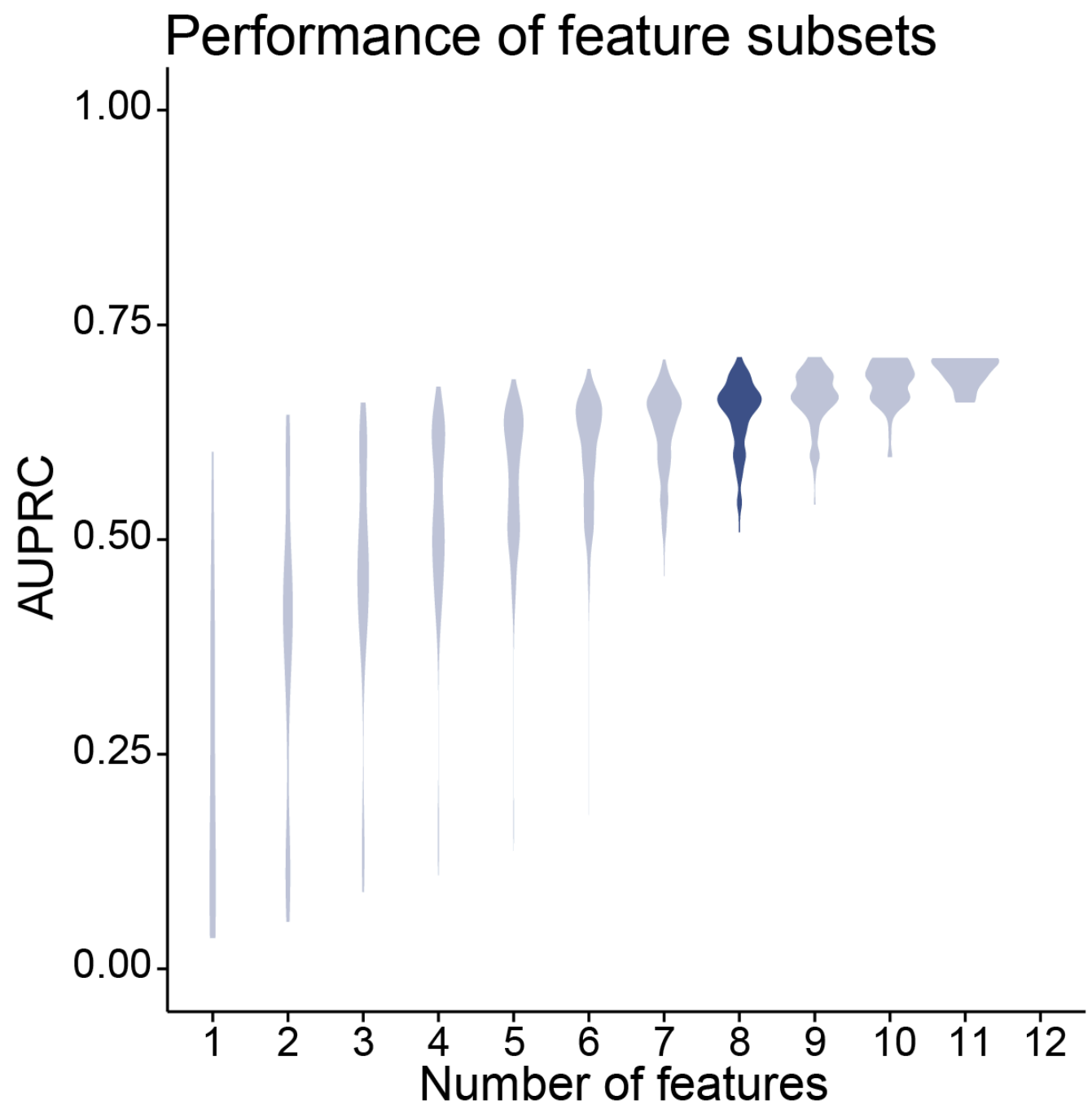

Supplementary Fig. N6.1 | Performance of different ENCODE-rE2G feature subsets by subset size
Violin plots showing AUPRC on the CRISPR benchmark for all possible subsets of the 12 input features for each subset size. The distribution of AUPRC for 8-feature subsets, which contains the final ENCODE-rE2G model is highlighted.

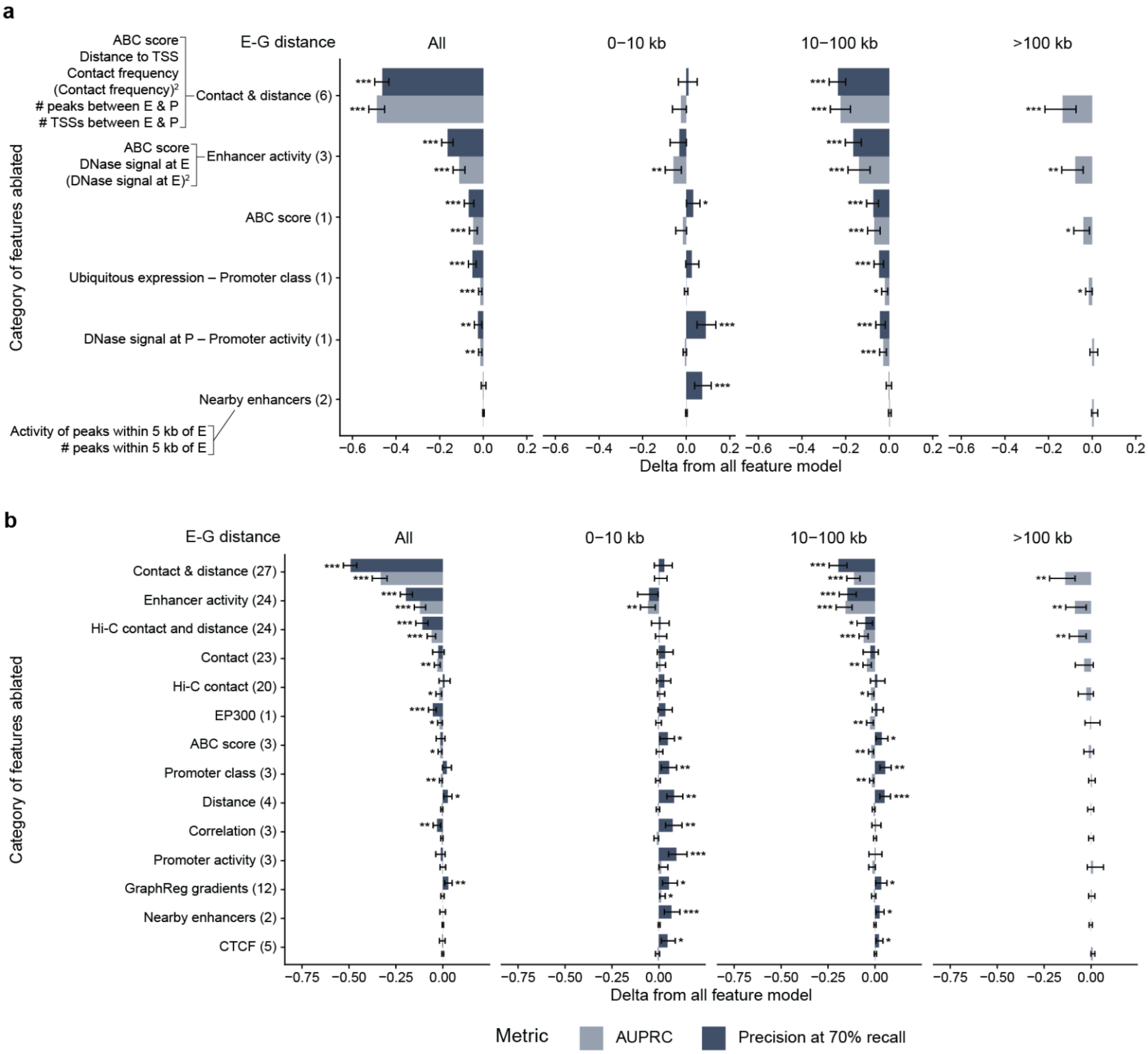

Supplementary Fig. N6.2 | Feature categories ablation on ENCODE-rE2G and ENCODE-rE2G^{Extended}

a, Effect of different feature categories on predicting CRISPR-validated E-G pairs for ENCODE-rE2G. Each feature category is removed from the feature set, then the model is retrained and evaluated to determine the change in performance reduction. The number of ablated features is indicated in parentheses, and the features in each category are listed to the side. The change in performance is shown for E-G pairs for different distance-stratified ranges; precision at 70% recall could not be computed for any model for the >100 kb category because no model reached 70% recall. Error bars: 95% range of AUPRC and precision values inferred via bootstrap (1,000 iterations). One, two, and three stars indicate P < 0.05, < 0.01, or 0.001, respectively, inferred via bootstraps (two-sided, 1,000 iterations, **Supplementary Methods 22**).

b, Same as **a**, but for ENCODE-rE2G^{Extended}. Features in each category are listed in **Supplementary Table 2**.

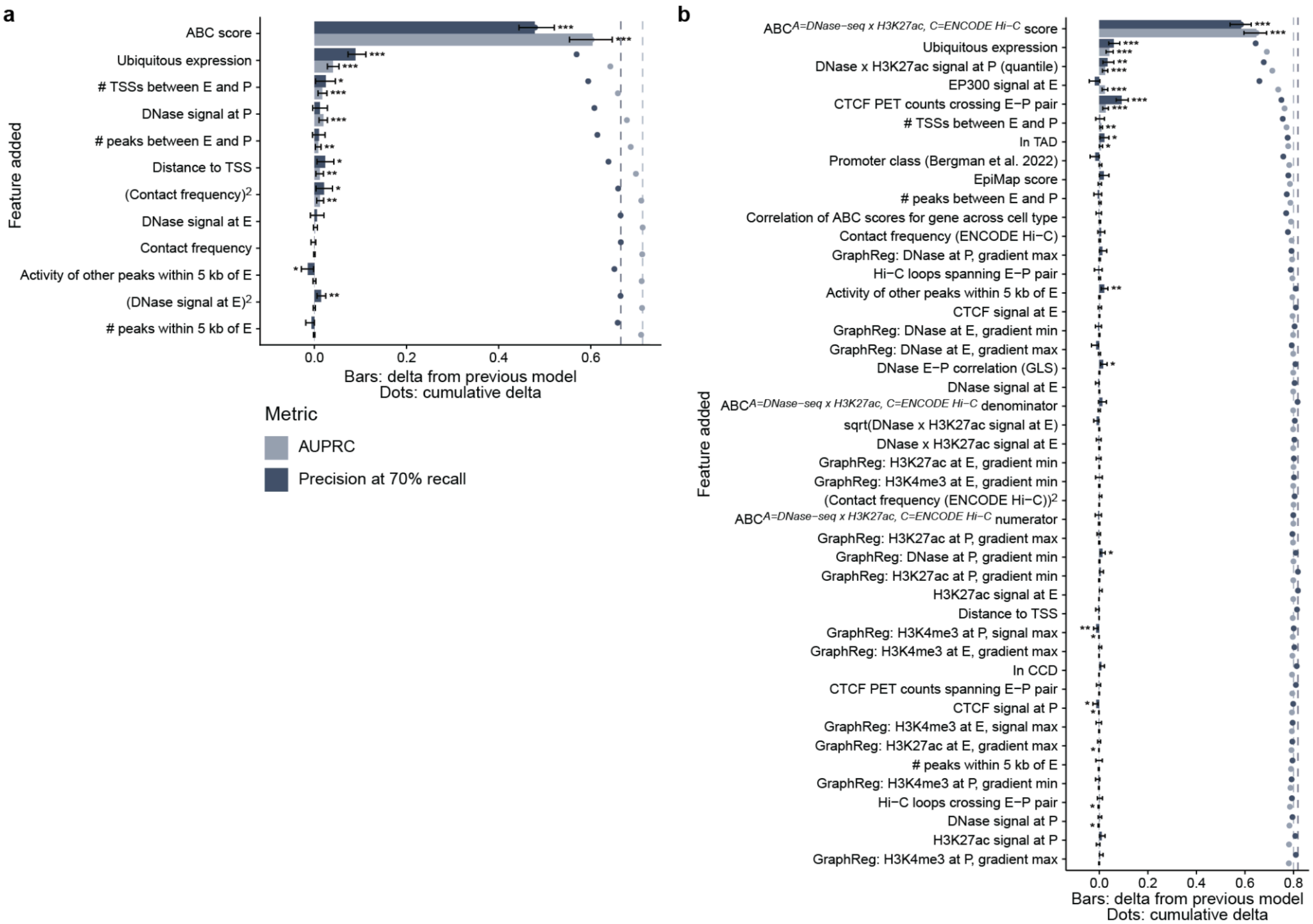

Supplementary Fig. N6.3 | Sequential feature selection on ENCODE-rE2G and ENCODE-rE2G^{Extended}

Features were sequentially added to each model based on maximum increase in AUPRC by adding a given feature. Bars indicate the change in AUPRC (light grey) and precision at 70% recall (dark grey) upon addition of each feature. Dots indicate the cumulative change in performance. Dashed vertical lines indicate the maximum performance. Error bars: 95% range of AUPRC and precision values inferred via bootstrap (1,000 iterations). One, two, and three stars indicate P < 0.05, < 0.01, or 0.001, respectively, inferred via bootstraps (two-sided, 1,000 iterations, **Supplementary Methods 22**).

a, Sequential feature selection for ENCODE-rE2G.

b, Sequential feature selection for ENCODE-rE2G^{Extended}.

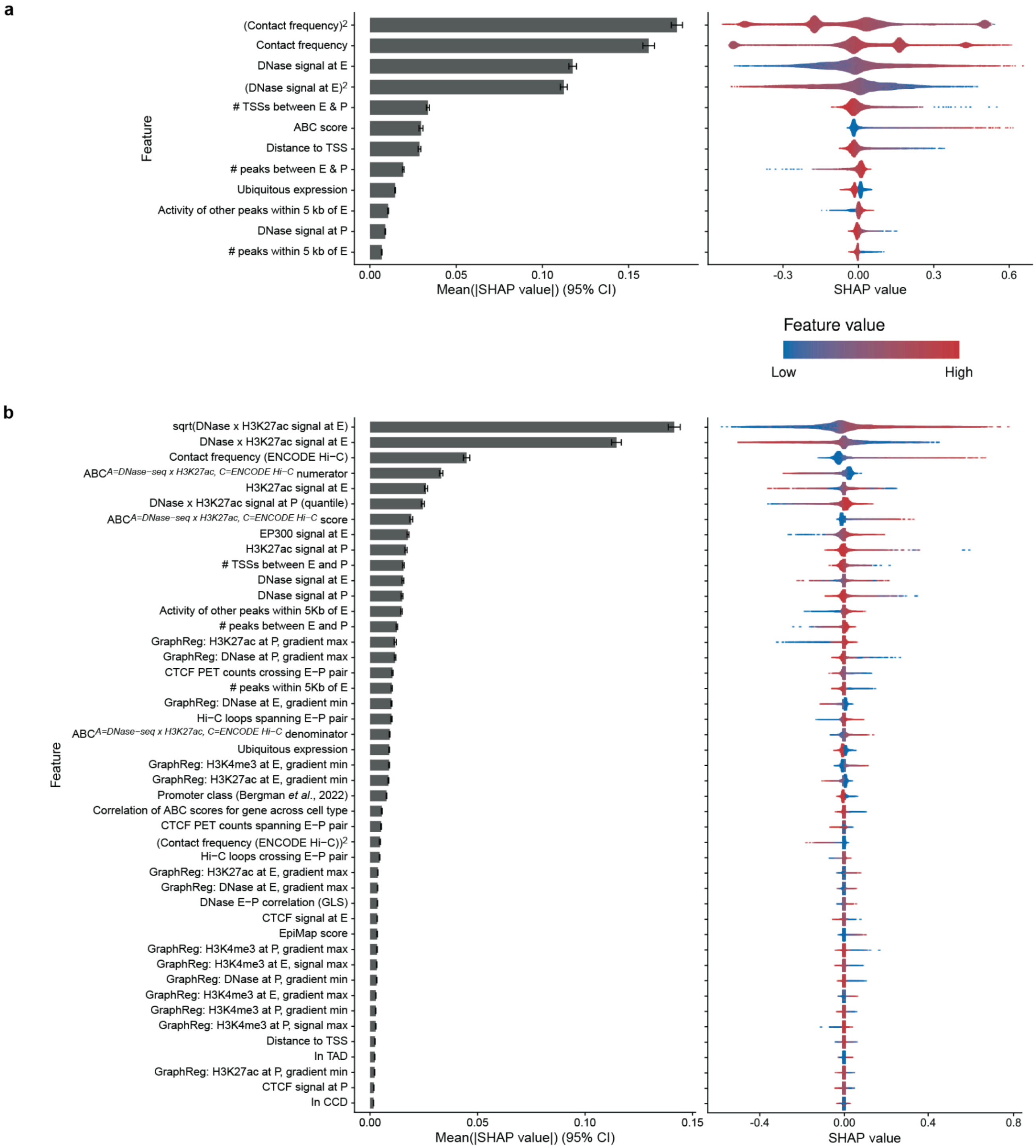

Supplementary Fig. N6.4 | SHAP scores of ENCODE-rE2G and ENCODE-rE2G^{Extended} models

a, SHAP scores of the 12 features used by ENCODE-rE2G model in a decreasing order. Left bar plot shows the mean of absolute SHAP scores for all the predicted EG pairs. Right dot plot shows the SHAP scores of each predicted EG pair color coded by the feature values. Error bars: 95% confidence intervals of SHAP values.

b, SHAP scores of all 45 features used by ENCODE-rE2G^{Extended} model in a decreasing order. Left bar plot shows the mean of absolute SHAP scores for all the predicted EG pairs. Right dot plot shows the SHAP scores of each predicted EG pair color coded by the feature values. Error bars: 95% confidence intervals of SHAP values.

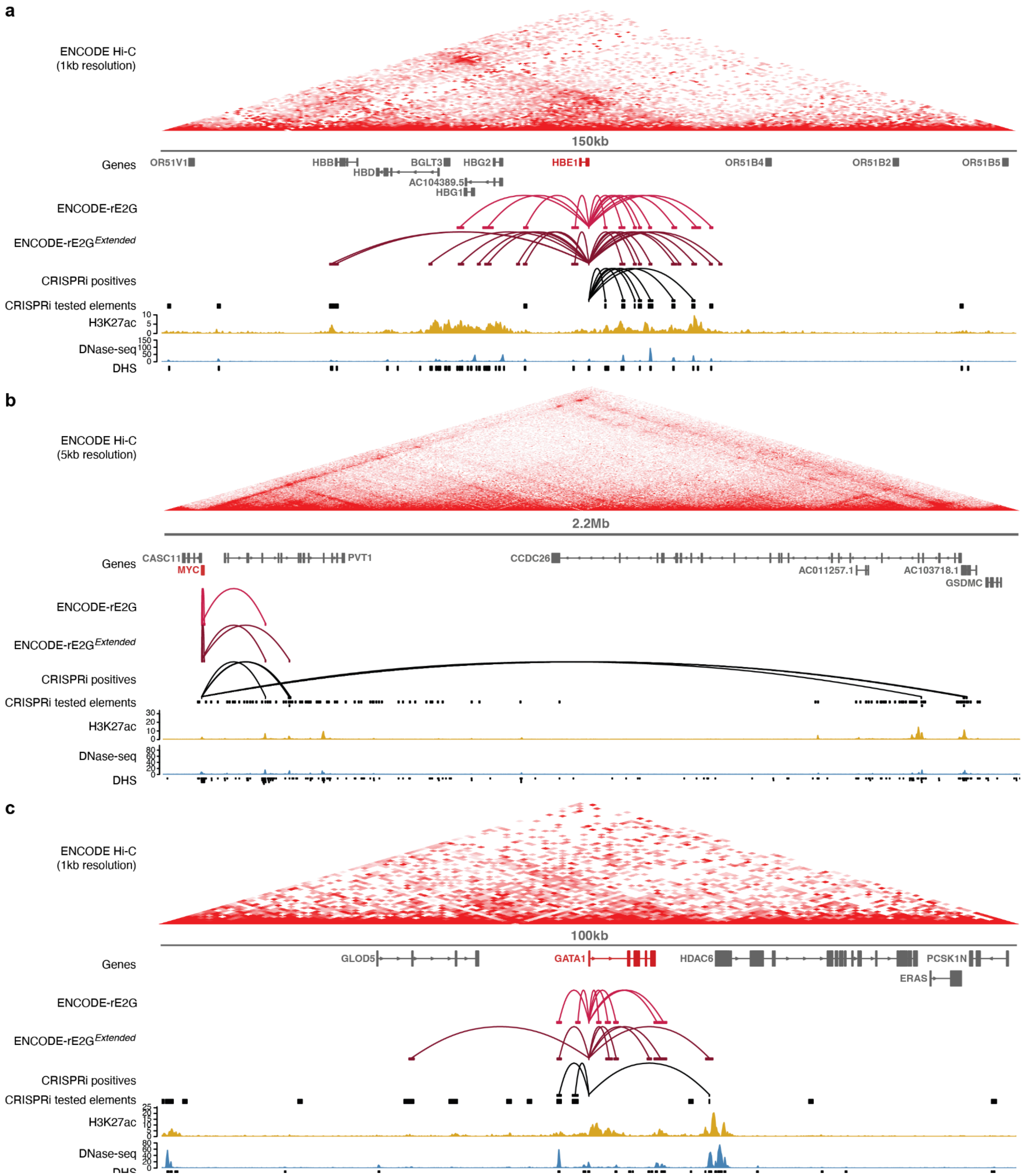

Supplementary Fig. 1 | K562 gene locus plots

Locus plots showing 3D contact (K562 ENCODE Hi-C), genes, predicted and CRISPR positive enhancer-gene regulatory connections, CRISPR tested elements, H3K27ac and DNase-seq data in K562 for *HBE1* (**a**), *MYC* (**b**) and *GATA1* (**c**). Genes for which enhancer-gene regulatory connections are shown are highlighted in red.

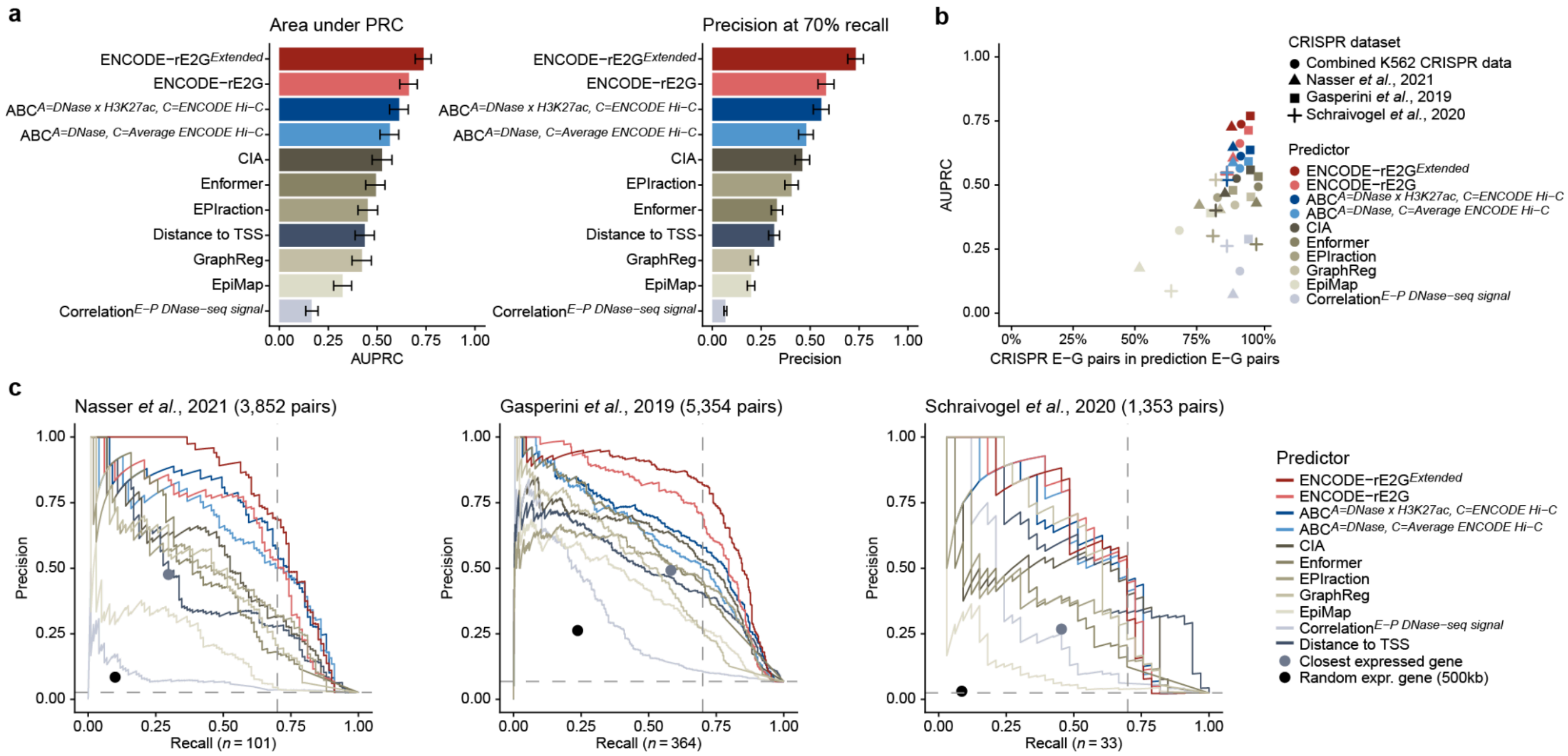

Supplementary Fig. 2 | Additional CRISPR benchmarks

a, AUPRC and precision at the inferred cutoff of 70% recall for quantitative predictors, benchmarked against the combined K562 CRISPR dataset. Error bars: 95% range of AUPRC values inferred via bootstrap (10,000 iterations).

b, AUPRC performance of predictive models versus the fraction of element-gene pairs in the combined K562 CRISPR dataset also found in the universe of element-gene pairs in the predictions from each model. A smaller fraction indicates that fewer CRISPR E-G pairs are also present in the predictions of a given model. The minimum score for each predictor was used as fill-in value for these missing cases in all benchmarking analyses (**Supplementary Methods 22**), generally lowering the performance of predictive models with low overlap with CRISPR benchmarking dataset.

c, Precision-recall curves showing the performance of predictive models benchmarked separately against each dataset in the combined K562 CRISPR data (10,356 tested element-gene pairs, 471 positives).

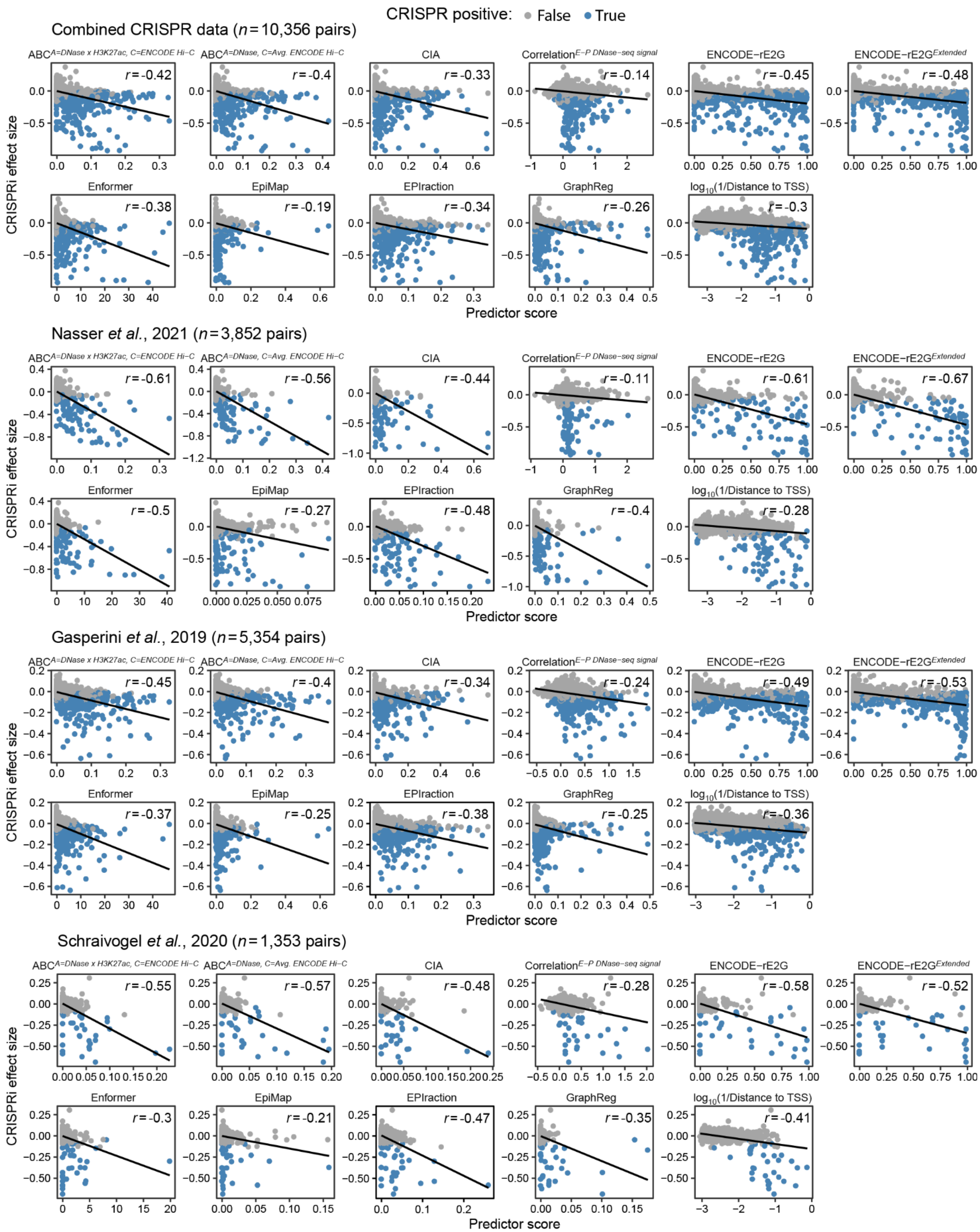

Supplementary Fig. 3 | Correlation between predictor scores and CRISPR effect sizes

Predictor scores versus observed effect size upon CRISPR perturbation for all E-G pairs in the different CRISPR datasets used to train ENCODE-rE2G. Effect sizes are percent changes in target gene expression and Pearson correlation coefficient (*r*) between predictor scores and CRISPR effect sizes is provided for each comparison.

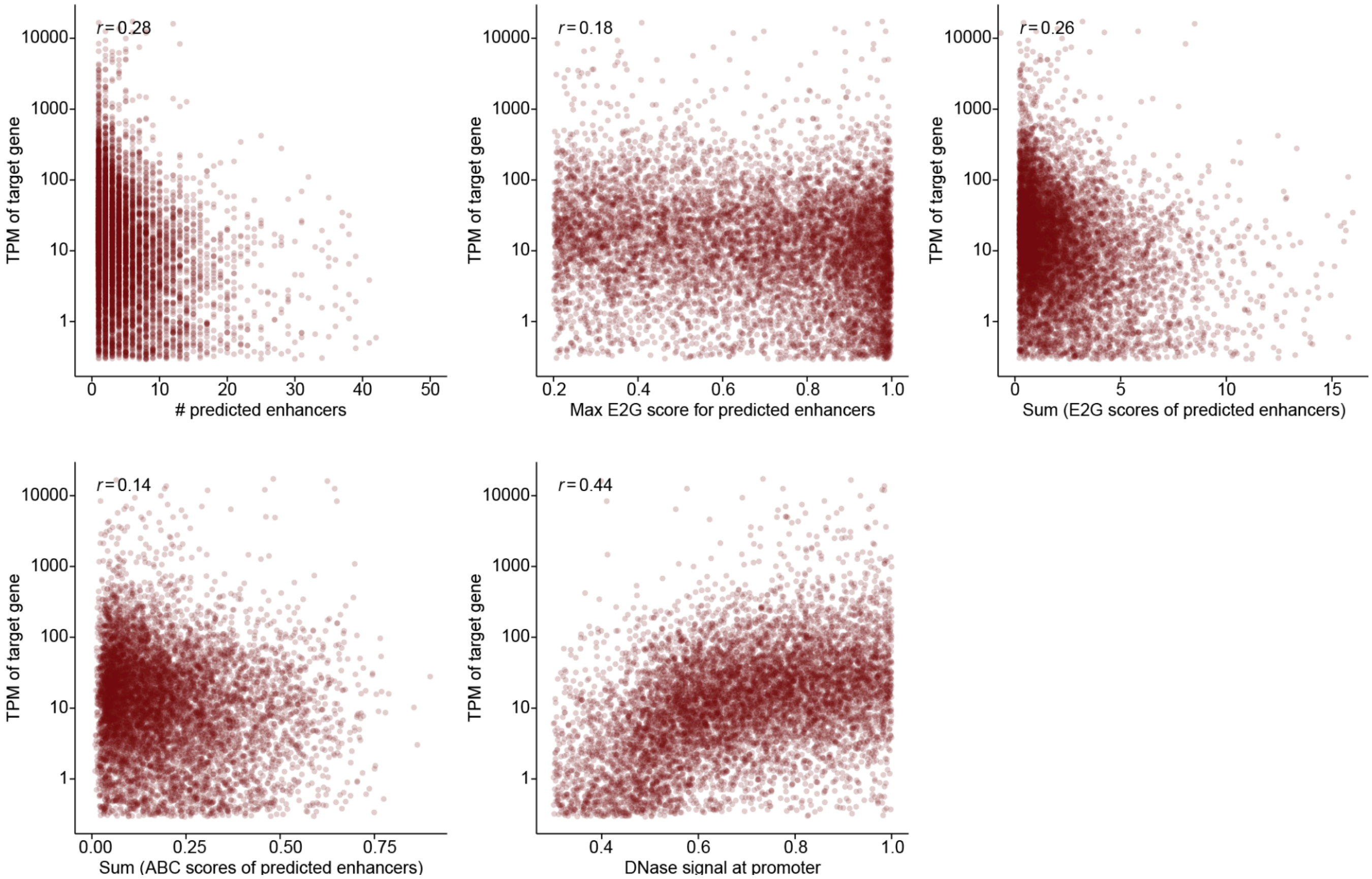

Supplementary Fig. 4 | Correlating ENCODE-rE2G predictions with target gene expression levels
Scatter plots showing TPM of genes in K562 computed from Gasperini *et al.*, 2019[3] as a function of enhancer predictions by ENCODE-rE2G and DNase-seq signal at the promoter (n = 13,135 genes). The Spearman correlation coefficient (ρ) is shown for each comparison.

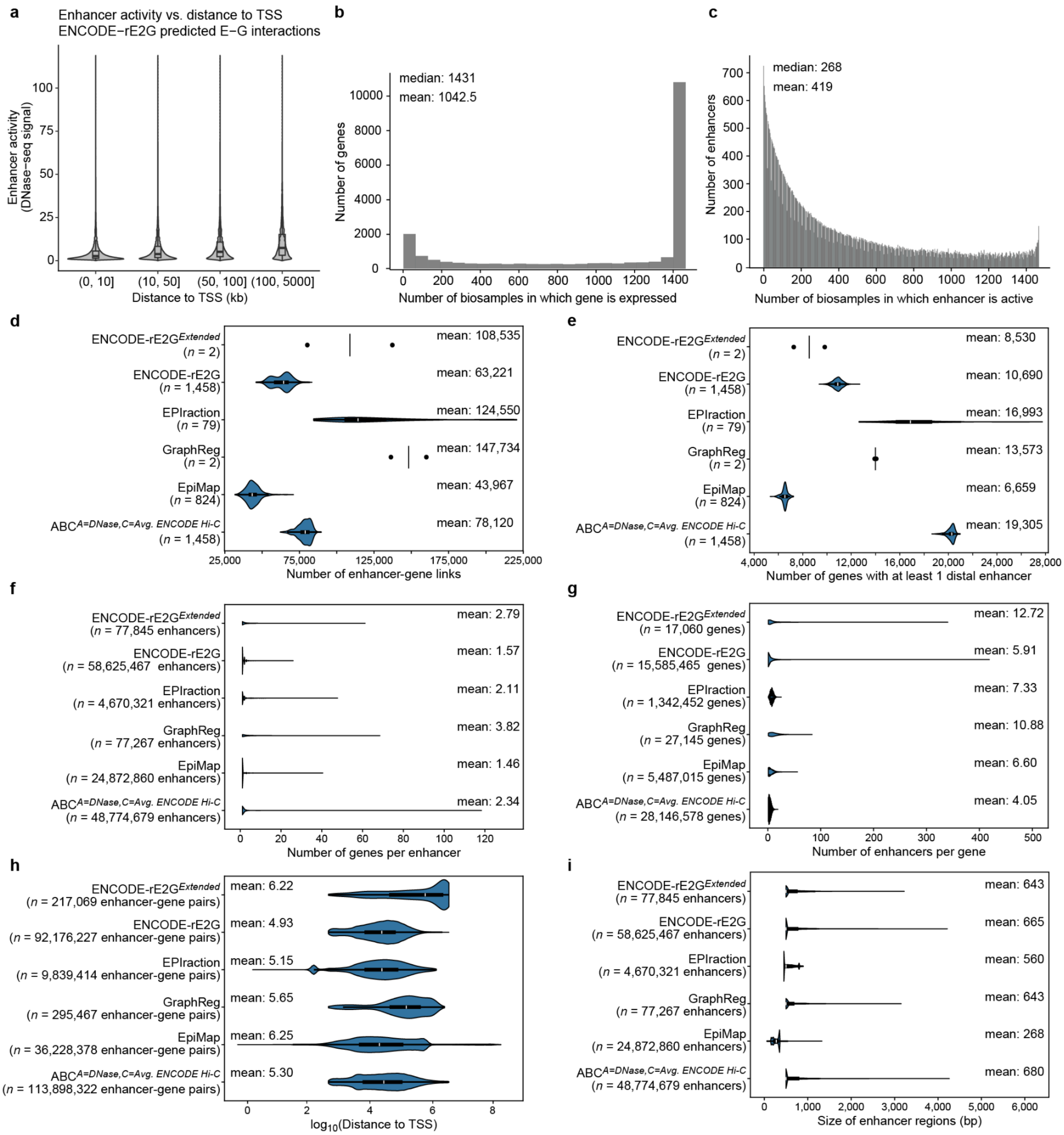

Supplementary Fig. 5 | Properties of enhancer-gene regulatory interactions

a, Enhancer activity (DNase-seq signal) of ENCODE-rE2G predicted enhancer-gene regulatory interactions as a function of distance to TSS across 1,458 DNase-seq experiments.

b, Gene Expression Across Biosamples. Genes were considered expressed in a given biosample if TPM > 1.

c, ENCODE-rE2G enhancer activity across biosamples. To account for shifting peak boundaries across biosamples, every element that was called as a significant enhancer element in at least one biosample was intersected with all other significant enhancer elements using `bedtools intersect`. Enhancers were considered the same if their boundaries were at least 50% overlapping in different biosamples and their biosample lists were aggregated to get the final biosample count.

d, Distribution of number of predicted "enhancer-gene regulatory interactions per biosample" across all biosamples.
e, Distribution of number of genes that are "regulated by at least one enhancer in a given biosample" across all biosamples.
f, Distribution of "number of genes per enhancer in a given biosample", including all genes in all biosamples that have at least 1 enhancer in that biosample.
g, Distribution of "number of enhancers per gene in a given biosample", including all genes in all biosamples that have at least 1 enhancer in that biosample.
h, Distribution of "distance to transcription start site (TSS)" for all enhancer-gene pairs in all biosamples.
i, Distribution of "enhancer size" across all biosamples.
Box plot elements in **a,d,e,h,i**: center line: median; box bounds: 25th and 75th percentile (interquartile range); whiskers extend to most extreme value within 1.5 x interquartile range from the box.

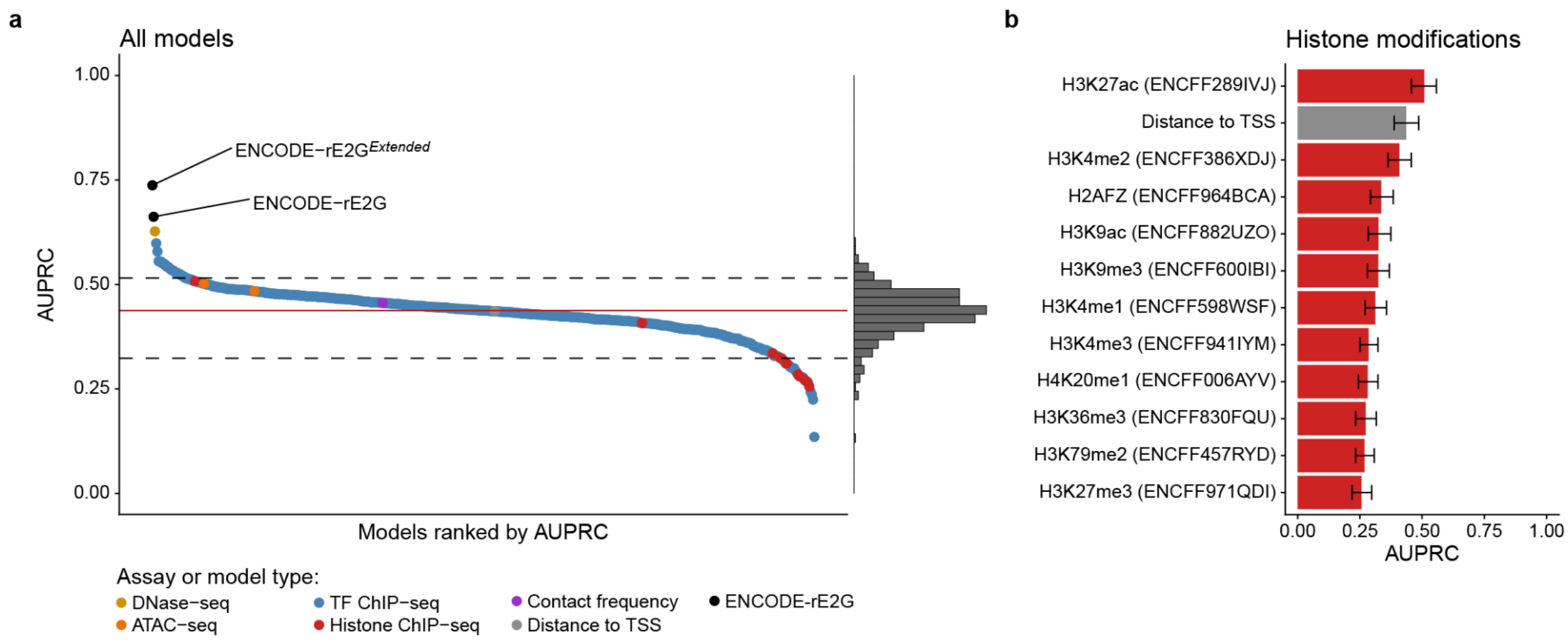

Supplementary Fig. 6 | Features of enhancer activity

a, Area under Precision-Recall Curve (AUPRC) performance of Activity-By-Contact (ABC) models using different ENCODE chromatin assays (n = 523 ENCODE experiments) to estimate enhancer activity at predicting experimental results of CRISPRi data in K562 cells. Models were benchmarked against the combined K562 CRISPR dataset. Red line: median AUPRC across all models; dashed lines 90% range of AUPRC values. Performance of ENCODE-rE2G models are shown as reference.

b, AUPRC of ABC models using ChIP-seq from different histone modifications to estimate enhancer activity. Error bars: 95% range of AUPRC values inferred via bootstrap (10,000 iterations).

Supplementary Table Descriptions

Supplementary Table 1 | Collected enhancer-gene predictions
A list of enhancer-gene linking models and other methods collected as part of this study. This table also contains parameters used for benchmarking analyses. Model versions from feature selection analyses are not listed individually.

Supplementary Table 2 | Features of enhancers, promoters, and enhancer-promoter pairs
A list of all molecular features of enhancers, promoters and enhancer-promoter pairs collected as part of this study to develop ENCODE-rE2G and ENCODE-rE2G^{Extended}.

Supplementary Table 3 | Performance of predictive model on the CRISPR benchmark
Performance of predictive models at predicting CRISPR E-G pairs in the CRISPR benchmarking analyses. Columns show area under the precision-recall curve (AUPRC), threshold at 70% recall, and precision and recall (held-out CRISPR dataset only) at the chosen threshold, including 95% value intervals for AUPRC, precision and recall from bootstrapping.

Supplementary Table 4 | Pairwise comparisons of predictive models on the CRISPR benchmark
Results of pairwise comparisons of performance metrics in the CRISPR benchmarking analyses. Columns show compared models, performance differences, and 95% confidence intervals, minimum, maximum values and *P*-values from bootstrapping differences (delta) in performance metric.

Supplementary Table 5 | eQTL benchmarking
Tissue-biosample pairs and numerical results from benchmarking predictive models of enhancer-gene regulatory interactions against finemapped eQTL variants.

Supplementary Table 6 | GWAS traits
List of traits and studies used for benchmarking models enhancer-gene regulatory interactions against fine-mapped GWAS variants.

Supplementary Table 7 | GWAS benchmarking results
Numerical results from benchmarking predictive models of enhancer-gene regulatory interactions against fine-mapped GWAS variants.

Supplementary Table 8 | ENCODE-rE2G summary statistics per biosample
Summary statistics for binary ENCODE-rE2G predictions of regulatory enhancer-gene interactions in 1,458 DNase-seq experiments.

Supplementary Table 9 | ENCODE-rE2G summary statistics per gene
Summary statistics per gene from binary ENCODE-rE2G predictions of regulatory enhancer-gene interactions in 1,458 DNase-seq experiments.

Supplementary Table 10 | OpenTargets L2G traits
List of OpenTarget study IDs used to benchmark the Locus-to-Gene (L2G) approach and matching GWAS traits used in this study.

Supplementary Table 11 | Pairwise *MYC* enhancer perturbation screen results
Effects of individual enhancer perturbations (percent change of *MYC* expression), effect of pairwise enhancer interactions (pairwise effects - individual effects; positive = super-additive effect), *P*-values and Benjamini-Hochberg adjusted *P*-values for enhancer interactions (**Supplementary Methods 29**).

Supplementary Table 12 | Input datasets for ENCODE-rE2G and ENCODE portal Accession IDs
ENCODE metadata for ENCODE-rE2G and ENCODE-rE2G^{Extended} models including Accession IDs for final predictions on the ENCODE portal.

Supplementary Table 13 | Input datasets for training ENCODE-rE2G and ENCODE-rE2G^{Extended}
ENCODE Accession IDs or URLs for input data used to compute features to train ENCODE-rE2G and ENCODE-rE2G^{Extended} models in K562, and apply ENCODE-rE2G^{Extended} in K562 and GM12878.

Supplementary Table 14 | Input files for DNase-DNase correlation
ENCODE Accession IDs for all DNase-seq files used as input data to compute correlation metrics between DNase-seq signal at cCREs and promoters. See Supplementary Methods 7 'Correlation of enhancer-promoter activity across cell types'.

Supplementary Table 15 | Input files for DNase-RNA correlation
ENCODE Accession IDs for all DNase-seq and RNA-seq files used as input data to compute correlation metrics between DNase-seq signal at cCREs and RNA-seq expression levels from genes. See Supplementary Methods 7 'Correlation of enhancer-promoter activity across cell types'.

Supplementary Table 16 | Input data for baseline predictors Synapse URLs
ENCODE Accession IDs for files used as input data to compute baseline predictors in 89 biosamples. Also contains urls for resulting baseline predictor files on www.synapse.org. See Supplementary Methods 16 'Generating baseline predictors'.

Supplementary Table 17 | Enhancer activity ABC input files
ENCODE Accession IDs for files used as input data to compute Activity-By-Contact modes using different chromatin assays to measure enhancer activity. See Supplementary Methods 26 'Additional analyses: Assaying enhancer activity'.

Supplementary Table 18 | Input datasets for GraphReg and ENCODE portal Accession IDs
ENCODE Accession IDs for features and input data that were used as input for the GraphReg model and resulting predictions. See Supplementary Methods 11 'GraphReg'.

Supplementary Table 19 | Metadata for EPIraction
Metadata and ENCODE Accession IDs for EPIraction predictions in 79 different tissues and cell types. See Supplementary Methods 10 'EPIraction'.

Supplementary Methods

1. **Genome build**
All coordinates in the human genome are reported using build GRCh38 unless otherwise specified.

2. **Gene reference file for ENCODE-rE2G**
We used the following set of genes and promoter coordinates when building ENCODE-rE2G and conducting all benchmarking analyses: https://github.com/EngreitzLab/ENCODE_rE2G/blob/dev/reference/CollapsedGeneBounds.hg38.TSS500bp.bed. This file contains one promoter per gene symbol, which we defined as a 500 base pair region centered around the RefSeq TSS with the largest number of coding isoforms. To curate the set of genes, we matched the gene symbols with ENSEMBL IDs using the HUGO database[38] and manual annotation, then used the GENCODE v29 database to annotate each gene with its "gene type". We retained genes that 1) were included in the GENCODE v29 database and 2) had a gene type of "protein_coding," "processed_transcript," or "lincRNA" for a total of 20,678 genes.

3. **Defining a set of biosamples for ENCODE-rE2G and ABC enhancer-gene predictions.**
We calculated ENCODE-rE2G and ABC$^{A=DNase,\ C=Average\ ENCODE\ Hi\text{-}C}$ predictions for 1,458 ENCODE DNase-seq experiments, referred to as biosamples in this study. Each biosample measured DNase-seq accessibility for a cell type and treatment and/or genetic modification combination, covering 369 unique cell types and tissues. The full list of biosamples, including donor, treatment and genetic modification information is reported in (**Supplementary Table 12**).

4. **Downloading and pre-processing DNase-seq data for ENCODE-rE2G and ABC predictions**

    4.1. *Downloading metadata.* Using the ENCODE API, we downloaded metadata for available bam and fastq files from all released DNase-seq experiments using the following queries:

    Metadata for ENCODE DNase-seq bam files:
    https://www.encodeproject.org/metadata/?type=Experiment&status=released&assay_title=DNase-seq&assembly=GRCh38&files.file_type=bam&replicates.library.biosample.donor.organism.scientific_name=Homo+sapiens

    Metadata for ENCODE DNase-seq fastq files:
    https://www.encodeproject.org/metadata/?type=Experiment&status=released&assay_title=DNase-seq&assembly=GRCh38&replicates.library.biosample.donor.organism.scientific_name=Homo+sapiens&files.file_type=fastq

    We filtered the list of available DNase-seq bam files for released ENCODE4 versions using the GRCh38 genome build using 'File status == "released"', 'File analysis title == "ENCODE4*"' and 'File assembly == "GRCh38"'. Using the file accession identifiers we merged the sequencing run type information ('single-ended' or 'paired-ended') from each fastq file to the metadata of the corresponding bam file. Finally, for experiments using single-ended reads or a combination of single-ended and paired-ended reads, we retained only the bam files from 'unfiltered alignments.' For experiments using only paired-ended reads, we kept bam files from the filtered 'alignments.' Files from all

biological and technical replicates per experiment were used as input for a given biosample (**Supplementary Methods 8, 14**).

DNase-seq bam files used for each biosample are provided in **Supplementary Table 12** and we provide the code to reproduce our filters on GitHub: https://github.com/EngreitzLab/ENCODE-Distal-Regulation-Paper/tree/main/process_dnase_metadata

For ENCODE-rE2G^{Extended} and ENCODE-rE2G models with additional assays we manually selected input files for additional assays. Experimental inputs for each of these models are provided in **Supplementary Table 13**.

4.2. *Data preprocessing.* We processed the bam files differently depending on whether the bam files were paired-ended or single-ended.

For single-ended bam files, we removed reads that were unmapped, mate unmapped, not primary alignment, or failing platform (-F 780), and further filtered out multi-mapped reads with MAPQ < 30 (-q 30). This filtering was performed using the command `samtools view -F 780 -q30 -u single-ended.bam`. Single-end bam files were not further filtered to remove duplicate fragments, because duplicate fragments are difficult to detect with single-end data and running removal of duplicate reads would lead to undercounting of the true number of duplicate fragments giving the high complexity and sequencing depth of these DNase-seq datasets.

For paired-ended bam files, we directly downloaded the "filtered" alignments from ENCODE. These bam files were previously processed by the encode atac-seq-pipeline and encode chip-seq-pipeline filtering steps. Filtering steps are as follows: We removed reads that were unmapped, mate unmapped, not primary alignment, failing platform and PCR or optical duplicates (-F 1804), as well as multi-mapped reads with MAPQ < 30 (-q 30). We retained properly paired reads (-f 2). This filtering was performed using the command `samtools view -F 1804 -q 30 -f2 -u paired-ended.bam`, (samtools v1.7). We marked and filtered PCR duplicates using Picard's MarkDuplicates (Picard v1.126).

5. Defining candidate elements and element-gene pairs for predictive models and annotations

We defined a set of candidate elements in each biosample based on ENCODE DNase I hypersensitivity or ATAC-seq data using an approach similar to that we previously described for the ABC model[1]. We used this same set of candidate elements to construct the following predictive models: ENCODE-rE2G, ABC, CIA, GraphReg, and baseline models.

5.1. *Calculating candidate elements.* To define candidate elements we started by calling peaks from DNase-seq and ATAC-seq bam files with MACS2 (v2.2.9.1) using parameters recommended by the ENCODE guidelines for chromatin accessibility assays (`--shift -75 --extsize 150 --nomodel`). For experiment accessions that have multiple biological and technical replicates, we passed all replicate bam files as input to pool all available reads per experiment. We initially considered all peaks with $P < 0.1$ and then removed any peaks overlapping regions of the genome that have been observed to accumulate anomalous number of reads in epigenetic sequencing experiments (blacklisted regions, downloaded from

https://sites.google.com/site/anshulkundaje/projects/blacklists). We then counted DNase-seq (or ATAC-seq) reads overlapping these peaks and kept the 150,000 with the highest number of read counts. We then resized these peaks to 500-bp in length centered on the peak summit. To this peak list, we added 500-bp regions centered on the transcription start site of all genes. Any overlapping regions resulting from these additions or extensions were merged.

We define these extended and merged peaks as candidate elements. We classified each candidate element as a promoter, genic or intergenic element. Promoter elements are those that are within 500 bp of any TSS contained in the promoter reference file described in **Supplementary Methods 2**. Genic elements are those contained within any annotated gene body. Intergenic elements are all other candidate elements. We denote any genic or intergenic element as a 'distal' element.

5.2. *Computing a list of candidate element-gene pairs.* We generated a list of candidate element-gene pairs to consider in predictive models using the list of elements described in **Supplementary Methods 5.1** and the list of promoters described in **Supplementary Methods 2**, including all pairs located within 5 Mb of one another.

6. Annotating enhancer-promoter pairs with epigenomic measurements and genomic features

We systematically computed features of enhancer-promoter pairs, including using measurements of chromatin state, measurements/estimates of 3D contact frequencies, and other features including genomic distance and element density (**Supplementary Table 2**).

6.1. *Chromatin state at enhancers and promoters.*

6.1.1. *Calculation of DNase, H3K27ac, and other ChIP-seq signals.* We first utilized the bam files for the corresponding assays from the ENCODE portal. For information on any preprocessing we did, please refer to S4.2 on data preprocessing. Once the bam files have been preprocessed, we proceed to use `bedtools coverage -counts` (bedtools v2.26.0) to count reads for candidate element regions, gene bodies and gene promoter regions. We calculated cell-type specific enhancer activity for all candidate enhancers by computing the geometric mean of DNase-seq (and, if applicable, H3K27ac ChIP-seq count reads) because we expect that strong enhancers and strong promoters would have strong signals for both. Once DNase-seq (and, if applicable H3K27ac ChIP-seq count reads) have been computed for candidate element regions, gene bodies and gene promoter regions, we quantile normalized ("rank normalized") these values with a reference located here: https://github.com/broadinstitute/ABC-Enhancer-Gene-Prediction/blob/main/reference/EnhancersQNormRef.K562.txt . This quantile normalization reference was generated using K562 predictions made in hg19 for the original ABC paper[1]. We first distinguish quantile normalization references for promoters and non-promoter regions respectively. Generally, we ranked the promoters in K562 by their magnitude, calculated the average value for all promoters with the same rank and substituted the values of all promoters occupying the same rank. This was repeated for non-promoter regions. This quantile normalization step is key in ensuring that we can compare read counts, and any downstream features (such as the ABC score), across different cell types. Additionally, quantile normalization is utilized to minimize technical variability that might have been introduced in the experimental setup. We've found that using this reference works well across

hg19 and hg38 genome builds. Code for these calculations can be found by running the default parameters in the rule `neighborhoods.smk` located here: https://github.com/broadinstitute/ABC-Enhancer-Gene-Prediction/blob/main/workflow/rules/neighborhoods.smk

6.2. *3D contact frequencies from Hi-C.* We calculated 3D contact frequencies from either cell type-specific or cell type-average (Megamap contact maps) Hi-C data using a similar approach as previously described for the ABC model[1].

6.2.1. *Calculating contact frequency from Hi-C data*. For 3D contact values from Hi-C used for predictions with ENCODE-rE2G^{Extended} and the ABC model, we used our Hi-C preprocessing approach previously described for the ABC model[1], with slight modifications. Specifically, we used normalized ENCODE Hi-C contact maps (at 5-kb resolution), and processed these maps in the following steps:

For rows and columns from ENCODE Hi-C experiments corresponding to SCALE normalization factors < 0.25, we did not use SCALE normalization (these typically correspond to 5-kb bins with very few reads). Instead, we linearly interpolated the Hi-C signal in these bins by calculating an expected value based on power-law fit (**Supplementary Methods 6.2.3** for more information on how this power law relationship is calculated).

Each diagonal entry of the Hi-C matrix was replaced by the maximum of its four neighboring entries. As previously described[1], this step is taken because the diagonal of the Hi-C contact map corresponds to the measured contact frequency between a 5-kb region of the genome and itself. The signal in bins on the diagonal can include restriction fragments that self-ligate to form a circle, or adjacent fragments that re-ligate, which are not representative of contact frequency. Empirically, we observed that the Hi-C signal in the diagonal bin was not well correlated with either of its neighboring bins and was influenced by the number of restriction sites contained in the bin.

In order to normalize the Hi-C matrix to imitate a doubly stochastic matrix, we divided every contact value by the mean of the accumulated sum of Hi-C contact values in that row.

We then computed contact frequencies for an element-gene pair by rescaling the data as follows:

- We start by subsetting rows of the entire 2D Hi-C contact matrix in order to control for memory usage. We set the contact of the element-gene pair to the Hi-C signal at the bin of this row corresponding to the midpoint of E. For element-gene pairs that do not have a corresponding contact value (i.e., NaN), we set contact to zero.
- For distances greater than 5 kb, we added a small adjustment (pseudocount) based on the power law expected count at a given distance threshold (as predicted by the power-law relationship between contact frequency and genomic distance). The distance threshold is usually determined by the resolution of the Hi-C data used. Distances less than 5 kb were given the pseudocount computed at 5 kb. We compute the power law by utilizing the steps in **Supplementary Methods 6.2.3**.

- We found that different Hi-C datasets have slightly different power-law parameters. To weight all cell types equally in generating an average Hi-C profile, we scale the Hi-C profile in a given cell type by the cell-type specific gamma parameter from the power law relationship in that cell type. The scaling factor at distance d is given by d ^ ($\gamma_{ref} - \gamma_{celltype}$), where γ_{ref} is the reference gamma parameter. For this study, we used γ_{ref} = 0.86.
- In order to handle cases of insufficient Hi-C coverage at promoter regions, we replaced the Hi-C contact of all links with that promoter with the corresponding powerlaw contact value.

These processing steps are implemented in: https://github.com/broadinstitute/ABC-Enhancer-Gene-Prediction/blob/main/workflow/scripts/predictor.py

6.2.2. *Megamap contact maps.* Comprehensive summed ENCODE Hi-C Megamap across solid and immune tissue samples, were generated by aggregating 65 ENCODE Hi-C datasets. Raw, unnormalized contact matrices from each dataset were summed at 10-basepair resolution to produce a combined contact map. Only intrachromosomal contacts between loci separated by ≤10 megabases, and with a mapping quality score ≥30, were retained. The aggregated contact map was subsequently normalized using coverage correction and SCALE to account for sequencing depth and systematic biases. The resulting dataset provides a unified representation of short-range chromatin interactions, within 10 megabases, across diverse tissue types. The Hi-C datasets included in this summation were: ENCSR847RHU, ENCSR335JYP, ENCSR351NAI, ENCSR321BHC, ENCSR908NSV, ENCSR518LPK, ENCSR998LLZ, ENCSR385MKW, ENCSR315OOJ, ENCSR348KEP, ENCSR771MVF, ENCSR160XGG, ENCSR044YKV, ENCSR168PIR, ENCSR387RQD, ENCSR595UCM, ENCSR749PID, ENCSR352EGR, ENCSR333BMU, ENCSR503LWK, ENCSR673UZF, ENCSR206OUF, ENCSR872ZCP, ENCSR942NSE, ENCSR374OJL, ENCSR967VFA, ENCSR530DID, ENCSR591SNM, ENCSR921BHU, ENCSR476RFQ, ENCSR812PIJ, ENCSR941KKW, ENCSR439ILZ, ENCSR173QPK, ENCSR874EIH, ENCSR892BZA, ENCSR753CKM, ENCSR298UUL, ENCSR498AFT, ENCSR173VDQ, ENCSR210LEF, ENCSR994DPD, ENCSR975NRV, ENCSR236EYO, ENCSR971CJS, ENCSR324EJR, ENCSR898ANZ, ENCSR556QHB, ENCSR502GIO, ENCSR972FIP, ENCSR941AMF, ENCSR068FXF, ENCSR538PNC, ENCSR421CGL, ENCSR557QRO, ENCSR165UJN, ENCSR797MWY, ENCSR500AJN, ENCSR529BKV, ENCSR434MBR, ENCSR446NJD, ENCSR782YIX, ENCSR462CIE, ENCSR305GRJ, ENCSR408HOG.

We downloaded this aggregated Megamap contact file from the ENCODE portal under accession ENCSR492BKE (https://www.encodeproject.org/aggregate-series/ENCSR492BKE) and calculated contact frequencies for DNase-only ENCODE-rE2G models using the Hi-C contact from this file as described in **Supplementary Methods 6.2.1**.

6.2.3. *Estimating the power-law relationship between 3D contacts and distance.* It has been previously shown that Hi-C contact frequencies generally follow a power-law relationship (with respect to genomic distance). We computed a

power-law fit for Hi-C datasets by first examining the relationship between genomic distance (binned at 5-kb resolution) and normalized 3D contact values. For each bin, we calculated a value for each distance bin by normalizing the Hi-C contact value (normalized over the total number of values in that distance bin). Finally, we log-transformed both the distance bins and mean hic contact values and fitted the data using linear regression. Once a line of best fit is obtained using the Hi-C data ($Contact \sim (hic\ distance\ bin) \cdot \gamma\ +\ c$), we extracted the slope and intercept values denoted as γ and c. We utilized the power-law relationship between 3D contact and distance described by these learned parameters (slope and intercept values) to impute missing 3D contact values as described in **Supplementary Methods 6.2.1** and to conduct certain analyses using the power-law in place of 3D contact data (*e.g.*, see **Supplementary Note 2**).

6.3. *Detecting CTCF contact domains (CCD)*
We first computed CTCF loop coverage for each base by summing up the PET count of all CTCF loops from in situ ChIA-PET that span the base. This base-pair resolution track was binned at 200-bp resolution by using the maximum signal in the bin. Then, we applied a Hidden Markov Model (HMM) with two hidden states on this track to segregate the genome into high-coverage and low-coverage domains based on their levels of CTCF interaction frequency. We call the domains with high CTCF loop coverage as CTCF contact domains (CCD). The HMM model is imported from the `sklearn.hmm` package for python (v3.8).

6.4. *Computing CTCF ChIA-PET features: "Loop Cross" and "Loop Contain"*
CTCF loops for K562 and GM12878 from ChIA-PET were downloaded from the ENCODE portal (ENCSR184YZV, ENCSR597AKG). 'Loop Contain' is given by the ratio of total PET count of all CTCF loops containing both the enhancer and the promoter over total PET count of CTCF loops containing the promoter. 'Loop Cross' is given by the ratio of total PET count of all CTCF loops across the enhancer and the promoter pair over total PET count of CTCF loops containing the promoter. All CTCF loops have a minimum PET count of 3 and maximum length of 500kb.

6.5. *ENCODE Hi-C loops and domains.* For measurements involving binary loop calls and domain calls from ENCODE Hi-C data, we obtained data from ENCODE Accessions ENCFF134HIZ and ENCFF173VDJ respectively. Binary loop and domain calls were converted to contain a start region, end region and contact value associated with each entry.

6.6. *Features related to promoter class.* We used 3 correlated metrics to define classes of promoters. (i) An enhancer "complexity" score, in which we previously calculated the degree to which ABC enhancers for a given gene are correlated across cell types[9]. Higher correlations indicate that the enhancer landscape for a gene is similar across cell types, whereas lower correlations indicate that the enhancer landscape differs. (ii) Promoter class as defined by Bergman and Jones et al.[24], in which a subset of "P2" promoters were measured to be less sensitive to distal enhancers, and then additional P2 promoters were identified across a genome based on logistic regression of sequence and chromatin features. (iii) Is this a ubiquitously expressed housekeeping gene? We defined ubiquitously expressed data based on FANTOM5 gene expression data as previously described[24].

6.7. *Features of genomic position*

6.7.1. *Distance to TSS.* Distance to TSS for each candidate element-gene pair was computed as the absolute distance between the TSS and the center of the candidate element.

6.7.2. *How many protein-coding TSSs (or other candidate elements) away is a specific candidate element from the promoter? (i.e., how many protein-coding gene TSSs are located between the enhancer and promoter? 0 = closest TSS).* Utilizing the full list of enhancer-gene predictions, we generated a bed file by starting at the midpoint of each enhancer and extending it to end at the promoter of the target gene. Each bed file now consists of the chromosome, the midpoint of the enhancer and the promoter of the target gene. Using `bedtools intersect` (bedtools v2.26.0), we intersected this bed file with the list of gene TSSs and counted the total number of elements intersecting each enhancer-gene connection, excluding the target gene promoter.

6.7.3. *How many other candidate elements away is a specific candidate element from the promoter? (i.e., how many other candidate elements are located between the enhancer and promoter? 0 = closest candidate element).* Utilizing the full list of enhancer-gene predictions, we generated a bed file by starting at the midpoint of each enhancer and extending it to end at the promoter of the target gene. Each bed file now consists of the chromosome, the midpoint of the enhancer and the promoter of the target gene. Using `bedtools intersect` (bedtools v2.26.0), we intersected this bed file with the list of candidate elements and counted the total number of elements intersecting each enhancer-gene connection.

6.7.4. *How many other candidate elements are within 5 kb of a specific element? (count or quantitative sum of activities, cell-type specific).* For each candidate element, we annotated the element with either the count or summed enhancer activity of all other nearby candidate elements within 5 kb. (An element is counted if both its start and end coordinate are within 5 kb of the midpoint of the query element). For enhancer activity, we used the definition of the corresponding ABC model (see **Supplementary Methods 8**), *i.e.* using quantitative H3K27ac and DNase signals for certain models including ENCODE-rE2G^{Extended} and only DNase signals for the DNase-only models including ENCODE-rE2G. The code to generate these features and those from the above section is implemented here: https://github.com/EngreitzLab/ENCODE_rE2G/blob/main/workflow/scripts/feature_tables/gen_new_features.py.

7. Correlation of enhancer-promoter activity across cell types

cCRE-TSS pairs were generated using ENCODE4 cCREs obtained from http://users.wenglab.org/moorej3/Registry-cCREs-WG/V4-Files/GRCh38-cCREs.V4.bed.gz and the curated RefSeq TSS list. DNase-seq reads were counted within 100bp from the center of each cCRE and within a 500bp window centered on each TSS across 89 ENCODE biosamples (**Supplementary Table 14**). DNase-seq reads were normalized for library size to 1 million reads per biosample across all cCREs, respectively all TSS, and log transformed with adding one pseudocount. Pearson and Spearman correlation coefficients were calculated for each cCRE - TSS pair within 1Mb using R (4.1.1). Generalized Least Square (GLS) regression models were used to model DNase-seq reads at TSSs as a function of DNase-seq reads at cCREs within 1Mb. To account for global correlation among biosamples, a correlation matrix between all samples was computed from normalized DNase-seq reads in all cCREs. The gls function from the nlme (3.1-162) R package was used to fit regression models using the correlation matrix as correlation structure. The cCRE coefficients were extracted from the fitted models and used as measurement of correlation between cCRE and TSS activity.

For DNase-RNA correlations, RNA-seq data for 96 ENCODE PolyA+ RNA-seq samples (**Supplementary Table 15**) was downloaded and normalized to 1 million reads per sample. The same correlation approach as described above was applied, but instead of promoter accessibility, DNase-seq accessibility at cCREs was correlated with library size normalized and log transformed RNA-seq TPM values from genes with promoters within 1Mb of cCREs.

The snakemake pipeline used to compute correlation of enhancer-promoter activity across cell types is available on GitHub: https://github.com/argschwind/ENCODE_eg_correlation_predictors.

8. Applying the Activity-by-Contact model to ENCODE4 data

8.1. Updates to the Activity-by-Contact Model. We applied the Activity-by-Contact model using the same formula as previously described[1], with updates and improvements to the processing code, data inputs, and quality control metrics:

8.1.1. Enabling biological and technical replicates when defining candidate elements. We enabled the calling of peaks using MACS2 (v2.2.9.1) on all replicates of either DNase-seq or ATAC-seq as a measure of chromatin accessibility (previous versions of ABC used just one replicate to call peaks and define candidate elements). We then counted DNase-seq (or ATAC-seq) reads overlapping these peaks from all replicates, and report the average reads overlapping these peaks. We kept the 150,000 peaks with the highest number of read counts and annotated these as candidate regions. Here, we used 1 or more DNase-seq replicates as described above.

8.1.2. Enabling biological and technical replicates when calculating enhancer activity from DHS or ATAC-seq and H3K27ac ChIP–seq signals. We enabled the computation of the geometric mean of DNase-seq or ATAC-seq (and, where applicable, H3K27ac ChIP-seq) signals from all biological and technical replicates. For every enhancer element, we calculated enhancer "activity" as the averaged read coverage from all replicates for DNase-seq or ATAC-seq (and, where applicable, H3K27ac ChIP-seq) signals. Here, we used 1 or more replicates as described above.

8.1.3. Updated peak calling. As implemented in ABC, we call peaks using MACS2 (v2.2.9.1) on all replicates of either DNase-seq or ATAC-seq as a measure of chromatin accessibility, using the parameters recommended by the ENCODE guidelines for chromatin accessibility assays (`--shift -75 --extsize 150 --nomodel`). These parameters more accurately define peaks based on the cut sites of the DNase I and Tn5 enzymes as opposed to ChIP-seq assays, which the default MACS2 (v2.2.9.1) parameters, which were previously used, are optimized towards.

8.1.4. Reproducibility. We improved overall ease and reproducibility of running ABC by implementing a Snakemake pipeline including conda environments to manage software dependencies, allowing users to easily apply the ABC model and generate predictions across different biosamples. We document this new pipeline here: https://abc-enhancer-gene-prediction.readthedocs.io/en/latest/

8.1.5. *Additional updates.* We updated the codebase to use ENCODE Hi-C data, including adjustments to the Hi-C preprocessing steps and include support for reading remote files as described in **Supplementary Methods 6.2.1**. We updated the ABC pipeline to support analyses in hg38, including by adding an hg38 reference promoter file (**Supplementary Methods 2**).

8.2. *Applying ABC pipeline to ENCODE4 data*. We applied this updated ABC pipeline to compute various versions of ABC scores using different combinations of input datasets. Importantly, different ABC score thresholds are used for each of these types of ABC models, selected based on obtaining 70% recall in comparison to CRISPRi perturbation data (see **Supplementary Table 1**):

$ABC^{A=DNase,\ C=Average\ ENCODE\ Hi\text{-}C}$. We computed one set of ABC predictions for 1,458 released ENCODE4 DNase-seq experiments, in which we used DNase-seq only to estimate activity, and Megamap ENCODE Hi-C data (**Supplementary Methods 6.2.2**) to estimate 3D contact frequency (**Supplementary Table 1**). The threshold used for $ABC^{A=DNase,\ C=Average\ ENCODE\ Hi\text{-}C}$ scores was 0.018.

$ABC^{A=DNase\ x\ H3K27ac,\ C=ENCODE\ Hi\text{-}C}$. We computed one set of ABC predictions for K562 and GM12878, the two Tier 1 samples, in which we used DNase and H3K27ac to estimate activity, and cell-type specific ENCODE Hi-C data to estimate 3D contact frequency (**Supplementary Table 1**). The threshold used for $ABC^{A=DNase\ x\ H3K27ac,\ C=ENCODE\ Hi\text{-}C}$ scores was 0.027.

In K562 cells, we computed various other versions of the model using different assays for Activity (**Fig. 5d**, **Supplementary Fig. 6**) and Contact (**Fig. 5e, Supplementary Note 2**).

For all ABC models, ENCODE Hi-C data was analyzed at 5-kb resolution unless otherwise specified.

9. Applying EpiMap to ENCODE4 data

We modified the EpiMap[39] correlation-based peak gene linking methodology to (1) update the genome build to GRCh38, (2) focus on distal enhancers, (3) de-bias compositional changes, and (4) better model peak-gene distance.

To do so, we utilized GRCh38 mappings of the ENCODE4 cCRE index and mapped histone modifications and DNase-seq profiles in EpiMap to these locations. To do this for all 833 EpiMap biosamples, we obtained the corresponding GRCh37 locations of these GRCh38 cCREs via UCSC liftOver. We then used UCSC bigWigAverageOverBed to get the signal associated with these regions over imputed and observed marks in EpiMap. To get active enhancers per biosample, we filter these cCREs to those overlapping at least 50% of one of the following ChromHMM states: EnhA1, EnhA2, EnhWk, EnhG1, EnhG2, EnhBiv (this step is different than the original EpiMap predictions, in which promoter and TSS-proximal ChromHMM steps were also used).

Then, we updated the core EpiMap peak-gene linking methodology. We utilized ZCA whitening to de-bias compositional changes in the EpiMap sample-by-cCRE matrices per-mark as well as in the RNA-seq FPKM matrix. When correlating cCREs to genes, we utilize weighted correlations that use weights from sample-sample correlations generated from the

sample-by-cCRE matrix. These weighted correlations weigh similar samples more to provide more accurate cell type specific cCRE-gene links.

Finally, we utilize an ABC-style normalization to better model peak-gene distances. When combining cCRE-gene links across ChromHMM states, we take the probability of links output from the model, multiply these by the ABC distance decay (Power=0.87), and then normalize all links per-gene such that the sum of all link scores per gene equals 1.

10. EPIraction

EPIraction[40] is an enhanced implementation of the ABC algorithm, which similarly uses epigenetic data to measure enhancer activity and Hi-C data to measure enhancer-promoter contact probabilities. In addition, gene expression data is used to determine whether a gene is active in a particular tissue. To calculate enhancer activity, EPIraction includes, in addition to H3K27ac and open chromatin, tissue-specific CTCF and H3K4me2 or H3K4me1 signals. This leads to a more accurate estimate of activity, in particular for enhancers that do not have H3K27ac signatures. To calculate contact probabilities between candidate enhancers and promoters, EPIraction uses high quality Hi-C data from the same or similar tissues as well as consensus Hi-C matrix generated from 29 intact Hi-C datasets. In the first step, EPIraction calculates the conventional Activity-by-Contact scores for every enhancer-gene pair within 2Mb from gene TSS. In the second step, EPIraction uses a case-based reasoning method that utilizes predictions in similar tissues to boost the ABC scores of tissue-specific enhancers.

For this study, we included EPIraction predictions across 79 tissues (**Supplementary Table 19**). The pipeline to compute EPIraction predictions is available on GitHub: https://github.com/guigolab/EPIraction.

11. GraphReg

GraphReg[41] is a chromatin-interaction-aware gene regulation model which uses 1-dimensional epigenomic signals as well as 3-dimensional genomic interaction graphs, extracted from Hi-C data, to predict the gene expression values by graph attention networks. As GraphReg is a distal-enhancers-aware predictive model, its feature attribution values for each gene could be informative for the task of E-G predictions. The methods described in this section are the same as GraphReg paper but the input epigenomic and Hi-C datasets are different. As a result, the trained models are different from the published models. Here we trained GraphReg models in two cell types K562 and GM12878 using three informative epigenomic features at 100bp resolution, DNase, H3K27ac, and H3K4me3, and extracted genomic interaction graphs from ENCODE Hi-C contact maps at the resolution of 5kb (**Supplementary Table 18**). We used HiC-DC+[42] with the false discovery rate (FDR) of 0.1 to extract the genomic interaction graphs containing the interactions up to 2Mb. For gene expression, we have used CAGE-seq, binned at 5kb to be consistent with the Hi-C graphs, and predicted it at all the bins. We have used chromosome-wise cross-validation for the predictions and held out two chromosomes for each test and validation sets and trained on the remaining chromosomes. As such, we have trained 11 GraphReg models. After training, the models are used for feature attributions. As we need to get the feature attributions of all the genes, we have used only one model and the gradient method for feature attribution to speed up the process. The process of calculating the gradients of the genes with respect to the input features works as follows. We first retain all the genomic bins that contain the TSSs' of the genes. If the minimum distance between TSS and the boundaries of the 5kb bin is more than 200bp, we assign only that bin to that gene, and if the distance between TSS and the right (left) boundary is less than 200bp, we assign that bin plus the right (left) 5kb bin to that gene. Then for each gene, we calculate the gradient of the model output for its assigned bins (1 bin or the sum of two bins) with respect to the 1D epigenomic

features in the input of the model using automatic differentiation in TensorFlow. This means that we will have three gradient values (one for each epigenomic feature used) at each 100bp bins for each gene. These gradient values are always non-zero for the promoter bins but could be zero for the distal enhancer regions if there are no E-G interactions.

For an initial set of GraphReg scores (denoted GraphReg$^{Raw\ Scores}$, see **Supplementary Table 1**), we use our previous approach[41] in which we use the gradient values and input epigenomic features to define a score which could be used for enhancer predictions as follows:

$$s[i, g] = |\, exp(x_{dnase}[i] - 1) \times dg/dx_{dnase}[i] + exp(x_{h3k27ac}[i] - 1) \times dg/dx_{h3k27ac}[i] \,|,$$

$$score_{e,g} = \Sigma_{k=-L:L} s[i_{mid} + k, g] / \Sigma_k (s[k, g]),$$

where x_{dnase} and $x_{h3k27ac}$ denote log-normalized ($log(x + 1)$) DNase, H3K27ac values used in the training of the GraphReg model (they are exponentiated back to their original values here); $dg/dx[i]$ denotes the gradient of gene g w.r.t. the epigenomic feature x at bin i; $s[i, g]$ denotes the absolute value of the dot product of the two epigenomic features with their corresponding gradients at the bin i for the gene g; i_{mid} denotes the index of the bin related to the summit of the enhancer element e; L is the one-sided window size around the enhancer summit; and $score_{e,g}$ is the score of enhancer e for the gene g. This unsupervised score worked well in the GraphReg paper where only the genes with at least 10 candidate enhancers and at least one true functional enhancer were considered, as the main goal was per-gene enhancer rankings. However, if the goal is to have a global score for every E-G pair in the genome, this unsupervised score performs suboptimally for the task of enhancer prediction (**Fig. 2b,c**).

12. Enformer

Enhancer-gene predictions using Enformer[43] were created for sets of element-gene pairs. For benchmarking the performance against CRISPR data, Enformer predictors were generated for all element-gene pairs in the combined CRISPR dataset. For benchmarking predictions against eQTL variants in GM12878, two lists of DNase peak-gene pairs were used: 1) a list of all MACS2 (v2.2.9.1) DNase peaks in GM12878 overlapping the filtered set of LCL eQTLs, linked to the overlapping variant's eGene (*n* = 2,187); 2) a list of all MACS2 (v2.2.9.1) DNase peaks in GM1278 overlapping a random selection of 10,000 distal, noncoding background SNPs, each linked to every gene within 1 Mb (*n* = 74,340).

For each set of element-gene pairs, Enformer predictions were made for pairs where the distance between the element and the gene was less than 100kb (due to Enformer's input sequence length of ~200kb). We focused the analysis on Enformer outputs corresponding to CAGE K562 tracks, and we considered the genomic position corresponding to each gene's primary TSS, as chosen by the Enformer model. For each gene, we computed gradients back to the input reference nucleotides for a 200kb region centered at the TSS and took the absolute value to score each single nucleotide. For each element, we calculated the gaussian-weighted mean with sigma = 200 of nucleotide scores in a 2kb window centered on the element. Finally, to normalize for different gene expression levels and compare sites across genes, we divided the element-gene pair scores by the mean absolute value of the nucleotide scores across the entire 200kb region for each gene.

13. CTCF loop-constrained Interaction Activity (CIA)

We implemented a "CTCF loop-constrained Interaction Activity" (CIA) model that combines enhancer activity with a measure of 3D contact estimated from CTCF ChIA-PET data. This model has similarities to the ABC model, in that it combines chromatin activity of enhancers and the 3D contact between enhancers and promoters. In the CIA model, enhancer activity (A_E) was

computed from the geometric mean of normalized DNase-seq and H3K27ac ChIP-seq signal, as defined in the ABC model. Because spatial interactions between enhancers and promoters are largely constrained by CTCF loops, 3D contact was characterized by CTCF Constraint ($CC_{E,P}$), *i.e.* the difference between total PET count of CTCF loops that contain the enhancer-promoter pair ($\Sigma_{CTCF\ loop\ i\ contains\ E\text{-}P}\ PET_i$) and the total PET count of CTCF loops that cross the enhancer-promoter pair ($\Sigma_{CTCF\ loop\ j\ crosses\ E\text{-}P}\ PET_j$). This term was normalized by the sum of PET count of CTCF loops that span the promoter pair ($\Sigma_{CTCF\ loop\ i\ contains\ P}\ PET_i+1$) to allow cross-gene comparison. We filtered out CTCF loops greater than 500kb because most of them were weak and can be noisy. The CIA model implemented in this manuscript is different from Luo *et al.*, 2023[44] in some respects, including that, in Luo *et al.*, 2023[44], only CTCF loops containing enhancer-promoter pairs are considered.

14. Training and applying ENCODE-rE2G logistic regression models

We trained multiple logistic regression models using different combinations of input features, including ENCODE-rE2G and ENCODE-rE2G^{Extended} (**Supplementary Tables 2, 13**). For ENCODE-rE2G, we used 12 initial features that can be computed from cell type-specific DNase-seq data, cell type-averaged Hi-C data, and genome annotations alone across 1,458 ENCODE experiments (**Supplementary Table 12**). For measurements of enhancer activity and 3D contact frequency, we included both first-order and second-order features to capture possible non-linear effects of these features (**Supplementary Tables 2, 13**). For ENCODE-rE2G^{Extended}, we used an extended set of 45 features available in ENCODE K562 and GM12878 cell lines (**Supplementary Table 13**). Our training dataset consisted of 10,356 element-gene pairs tested by CRISPR in K562 cells (**Supplementary Methods 18**). We first applied a variance-stabilizing transformation $log(|x| + \epsilon)$, with $\epsilon = 0.01$, to all the features before feeding them to a standard logistic regression model without regularization.

To evaluate ENCODE-rE2G's performance on the training CRISPR data, we used hold-one-chromosome-out cross-validation, meaning that we held out one chromosome as the test set to calculate performance and train the models on the remaining chromosomes. As our ensemble CRISPR dataset contains the E-G pairs in the chromosomes 1-22 and X, we did the cross-validation 23 times to get the predictions in all the E-G pairs across the genome. To apply the models to new cell types, we generated similar cell type specific feature tables as described above and applied the model to each candidate element-gene pair using a model trained on all 23 chromosomes in K562 cells.

All code for model training and feature analysis were run as implemented in the ENCODE-rE2G GitHub repository using the training-specific code in workflow/Snakefile_training (https://github.com/EngreitzLab/ENCODE_rE2G/blob/main/workflow/Snakefile_training).

A snakemake workflow to apply trained ENCODE-rE2G models to new input datasets is available in GitHub: https://github.com/EngreitzLab/ENCODE_rE2G.

15. Collection of previously published enhancer-gene predictions

We collected the following additional enhancer-gene predictions that have been previously published (**Supplementary Table 1**). If predictions were provided in hg19, we used UCSC tools LiftOver (v377) to lift them over to GRCh38.

15.1. *ENCODE2012 E2G.* Distal accessible elements were previously linked to gene promoters by looking at correlation of DNase I hypersensitivity across 125 cell and tissue types from ENCODE[21]. We downloaded these links from: ftp://ftp.ebi.ac.uk/pub/databases/

ensembl/encode/integration_data_jan2011/byDataType/openchrom/jan2011/dhs_gene_connectivity/genomewideCorrs_above0.7_promoterPlusMinus500kb_withGeneNames_32celltypeCategories.bed8.gz.

15.2. *Anderson 2014.* The transcriptional activity of enhancers and TSSs was previously linked using the FANTOM5 CAGE expression atlas[45]. We downloaded these predictions from: http://enhancer.binf.ku.dk/presets/enhancer_tss_associations.bed.

15.3. *Liu 2017*. Gene expression was previously correlated with five active chromatin marks (H3K27ac, H3K9ac, H3K4me1, H3K4me2 and DNase I hypersensitivity) across 56 biosamples, and these correlation links were then used to make predictions for the predicted enhancers (regions with the '7Enh' ChromHMM state) in 127 biosamples from the Roadmap Epigenome Atlas[22]. We downloaded these predictions from www.biolchem.ucla.edu/labs/ernst/roadmaplinking and made predictions using the confidence score.

15.4. *Granja 2019 (healthy)*. Single-cell ATAC-seq and RNA-sequencing data in peripheral blood and bone marrow mononuclear cells, CD34+ bone marrow cells and cancer cells from patients with leukaemia were previously analyzed and the ATAC-seq signal in accessible elements was correlated with the expression of nearby genes[46]. We downloaded these predictions from https://github.com/GreenleafLab/MPAL-Single-Cell-2019 and used the correlation in samples from healthy individuals as the quantitative score. Cell-type-specific links were not reported.

15.5. *Gao 2020*. EAGLE was previously used to predict enhancer–gene interactions across a number of human tissues and cell lines[47]. The method calculates a score based on six features obtained from the information of enhancers and gene expression: correlation between enhancer activity and gene expression across cell types, gene expression level of target genes, genomic distance between an enhancer and its target gene, enhancer signal, average gene activity in the region between the enhancer and target gene, and enhancer–enhancer correlation. We downloaded enhancer annotations for 104 cell types from http://www.enhanceratlas.org.

15.6. *Sheffield 2013*. The DNase I signal and gene expression levels were previously correlated using data from 112 human samples representing 72 cell types to identify regulatory elements and to predict their targets[48]. We downloaded these predictions from http://dnase.genome.duke.edu and used the correlation as the quantitative score. Cell-type-specific links were not reported.

15.7. *Cao 2017*. Correlations between gene expression and various enhancer features (for example, DNase1 and H3K4me1) were previously computed across multiple cell types to identify a set of putative enhancers[49]. Then, a sample-specific model is used to predict the enhancer gene connections in a given cell type. We downloaded the lasso-based JEME predictions in all ENCODE+Roadmap cell types from http://yiplab.cse.cuhk.edu.hk/jeme. We used the JEME confidence score as a quantitative score.

15.8. *TargetFinder*. A model was previously generated to predict whether nearby enhancer–promoter pairs are located at anchors of Hi-C loops[50]. TargetFinder predictions were downloaded from https://raw.githubusercontent.com/shwhalen/targetfinder/master/paper/targetfinder/combined/output-epw/predictions-gbm.csv and intersected with candidate E-G pairs. If the element and gene TSS overlapped with a predicted enhancer and promoter loop, we considered it a predicted enhancer-gene regulatory interaction.

15.9. *ABC (Nasser 2021)*. Full ABC prediction files for 131 cell types[9] were obtained from ftp://ftp.broadinstitute.org/outgoing/lincRNA/ABC/Nasser2021-Full-ABC-Output and lifted over from hg19 to GRCh38 using UCSC tools LiftOver (v377).

15.10. *Schraivogel 2020.* A random forest model trained on CRISPR enhancer perturbation data to predict enhancer-gene regulatory interactions from molecular features[4]. The published model trained on the targeted Perturb-seq (TAP-seq) CRISPR enhancer screen in the chromosome 8 and 11 regions was taken to make genome-wide predictions in K562 cells. Candidate enhancer elements were defined using the same strategy as for the TAP-seq enhancer screen, *i.e.*, DHS overlapping active enhancer chromatin states from GenoSTAN[51]. Candidate element-gene pairs were created with all protein-coding genes within 300 kb of elements and molecular features for each pair were computed in the same as the original publication: https://github.com/argschwind/TAPseq_manuscript/blob/master/scripts/chromatin_annotated_etps/genome_wide_etps.R

16. Generating baseline predictors

We generated several simple baseline predictors for the same 1,458 biosamples used to apply ENCODE-rE2G. Baseline predictors were built based on two different universes of elements: The first was based on ENCODE4 DNase-seq pipeline narrowPeak calls (DHS) (ENCPL848KLD) available through the ENCODE portal (www.encodeproject.org). The second was based on candidate elements defined in this study using MACS2 (v2.2.9.1) peak-calling (ABC) (**Supplementary Methods 5.1**). For each universe, all element-gene pairs were created by pairing candidate TSSs with all candidate elements within 1Mb.

For all 1,458 biosamples we generated the following baseline predictors based on DNase-seq peaks (**Supplementary Table 16**): 1) Distance from elements to the TSS calculated as the absolute distance between the center of the element and the TSS ("distance to TSS"); 2) Assign each element to the nearest expressed gene ("nearest expressed gene"), where "expressed" was approximated using promoter activity in the ABC model as previously described[1].

For 24 selected biosamples used in benchmarking analyses, we generated the same and additional baseline predictors for both universes of elements (**Supplementary Table 16**): 1) Distance from elements to the gene body calculated as the absolute distance between the center of the element and the gene body ("distance to gene"). If the element overlapped the gene, distance was set as 0; 2) Assign each element to the nearest TSS or gene ("nearest TSS or gene"); 3) Assign each element to the nearest expressed TSS ("nearest expressed TSS"), where "expressed" was defined as described above; 4) Assign each element to every TSS or gene within 100kb ("within 100kb of TSS or gene"); 5) Assign each element to every expressed TSS or gene within 100kb ("within 100kb of expressed TSS or gene"), where "expressed" was defined as described above; 6) Multiply the read-depth normalized DNase-seq signals within the element by 1/distance to TSS or gene ("reads by distance to TSS or gene"); 7) "reads by distance to TSS or gene" scores normalized to 1 per gene (a simplified version of the ABC score).

For 20 of these biosamples we obtained ENCODE H3K27ac ChIP-seq data and also computed "reads by distance to TSS or gene" scores based on H3K27ac signal within the element for both universes of elements (**Supplementary Table 16**).

All generated baseline predictors are available on Synapse (https://www.synapse.org/Synapse:syn58896208) and code used to compute baseline predictors is available on GitHub: https://github.com/argschwind/ENCODE_eg_baseline_predictors.

Not all candidate elements in the collected CRISPR datasets are part of the baseline predictor element universes and others can have different boundaries. For optimal baseline predictors in the CRISPR benchmarks, additional versions of the "distance to TSS", "distance to gene", "nearest TSS of Gene", "nearest expressed TSS or gene", "within 100kb of TSS" were computed based on the universe of all CRISPR element-gene pairs as part of the CRISPR benchmarking pipeline.

17. Defining classification thresholds for predictive models

We used performance in the CRISPR benchmarking analysis to define classification thresholds for all generated and collected predictive models. For each predictor, a threshold was chosen that corresponds to 70% recall on the combined CRISPR data (**Fig 2b, Supplementary Table 1**). These thresholds were then applied to create binary predictions of enhancer-gene regulatory connections in all cell types or biosamples to which a given predictor was applied.

18. Creating the combined K562 CRISPR training dataset

We developed a CRISPR model training and benchmarking framework to develop and compare predictive models to perturbation data from previous studies in which CRISPR perturbations were used to perturb candidate elements and effects on gene expression were measured in K562 cells. We collected and reprocessed 3 previously published CRISPR enhancer screen datasets from Nasser *et al.*, 2021[9] (itself comprised most of CRISPR screens from Fulco *et al.*, 2019[1]), Gasperini *et al.*, 2019[3] (Perturb-seq) and Schraivogel *et al.*, 2020[4] (TAP-seq). See **Supplementary Note 1** for more information on the overall approach.

18.1. Analyzing CRISPRi Perturb-seq/TAP-seq enhancer screen data. We have developed an analysis pipeline to identify regulatory enhancer–gene pairs from Perturb-seq experiments. For each perturbed element, differential expression testing between perturbed and unperturbed cells was performed to identify target genes. UMI counts were normalized for a size factor based on total counts per cell, excluding top 10% expressed genes per cell for size factor calculation, and then log-transformed. Cells carrying at least one guide RNA targeting a given element were tested against a specified number of control cells sampled from cells not carrying a perturbation for the element using two-sided differential expression tests implemented in MAST (v. 1.20.0)[52]. Benjamini-Hochberg false discovery rate was computed to correct for multiple testing for all tests performed across perturbed elements.

In addition, we also implemented a simulation-based power analysis to estimate the statistical power of every tested enhancer-gene pair to detect perturbation effects of specific effect sizes based on the number of perturbed cells and gene expression levels. UMI counts were simulated from negative binomial distributions with mean and dispersion parameters estimated for every gene using DESeq2 (v. 1.34.0)[53]. Mean expression levels across all cells were used as unperturbed expression levels and perturbation effects for each enhancer-gene pair were simulated by injecting a specified effect size (decrease in mean expression) for perturbed cells. To simulate variability in effect sizes (knockdown efficiency) for guides targeting the same enhancer, in each iteration a guide-guide variability value was randomly drawn from a normal distribution with mean 1 and standard deviation of 0.13 for each guide and added to the specified effect size. The standard deviation for this normal distribution was learned by analyzing guide-guide variability from FlowFISH experiments[1] across the range of observed effect sizes of significant enhancer-gene interactions. Simulated UMI counts were normalized, and differential expression tests were performed using the same approach as for the real perturbation data. To account for multiple testing of simulated perturbations in the

context of the *P*-value distribution of the given CRISPR screen, a nominal *P*-value cutoff corresponding to the FDR threshold used in the differential expression analysis of the observed perturbation data was applied. Simulations were repeated 20 times and statistical power for each pair was defined by the proportion of simulations in which the injected perturbation effect was detected as statistically significant.

18.2. *Processing of CRISPRi-Perturb-seq data from Gasperini et al., 2019.* Processed data containing UMI counts per cell and gene, and detected guides per cell were downloaded from GEO (accession: GSE120861). We recomputed guide-to-element assignments for our analysis. To do so, genomic coordinates of guideRNA binding sites were inferred using BLAT to align guide sequences to the hg19 genome sequence. Candidate elements were defined by extending the summits of the top 150,000 K562 DNase-seq peaks (see **Supplementary Methods 5,** ENCODE experiment ENCSR000EOT) by 250bp upstream and downstream and merging overlapping regions. Guides were assigned to targeted elements by overlapping guide binding sites with the generated candidate elements. For each targeted element, genes within 2Mb were tested for differential expression between perturbed cells and 5,000 randomly sampled control cells as described above. Power simulations were performed for all candidate element-gene pairs included in the differential expression analysis. Benjamini-Hochberg False Discovery Rate (FDR) was calculated for all tested element-gene pairs and a cutoff of < 5% was applied to identify positive hits for both differential expression and power simulations. The final dataset was compiled by filtering all tested E-G pairs for a distance to TSS between 1kb and 1Mb and any pairs where the enhancer overlapped the gene body of their respective target gene were removed, due to the ability of CRISPRi to repress a gene when targeted to its gene body[1,2]. Negative E-G pairs were further filtered for minimum statistical power of 80% to detect a 15% decrease in target gene expression upon perturbation.

18.3. *Processing of CRISPRi-TAP-seq data from Schraivogel et al., 2020.* Raw data was processed as in Schraivogel et al., 2020[4]. For each targeted element, all genes within the same genomic region (chr11 and chr8) were tested for differential expression between perturbed cells and 3,500 randomly sampled control cells with the same distribution across 10x lanes as perturbed cells as previously described[4]. Power simulations were performed for all element-gene pairs included in the differential expression analysis. Benjamini-Hochberg False Discovery Rate (FDR) was calculated for all tested element-gene pairs and a cutoff of < 5% was applied to identify positive hits for both differential expression and power simulations. The final dataset was compiled by filtering all tested E-G pairs for a distance to TSS between 1kb and 1Mb, and any pairs where the enhancer overlapped a GENCODE v29 annotated promoter or the gene body of their respective target gene were removed. Negative E-G pairs were further filtered for minimum statistical power of 80% to detect a 15% decrease in target gene expression upon perturbation.

18.4. *Processing of CRISPR enhancer perturbation data from Nasser et al.,* 2021[9]*.* This dataset contains mostly data from CRISPRi-FlowFISH DHS tiling experiments[1], with additional E-G pairs added from other published studies as previously described[1,9]. The processed dataset was downloaded from: https://raw.githubusercontent.com/EngreitzLab/ABC-GWAS-Paper/main/comparePredictorsToCRISPRData/comparisonRuns/K562-only/experimentalData/experimentalData.K562-only.txt and reformatted for our benchmarking framework.

18.5. *Combined CRISPR dataset.* We combined the three individual power-filtered datasets into one large, combined dataset. In case a given E-G pair was targeted in more than one dataset, one pair was chosen using the following heuristic: 1) Only positive E-G pairs were retained if there were any. 2) If there were no or more than one positive, one pair was chosen based on the highest statistical power to detect a 25% expression change.

19. Analyzing held-out CRISPR datasets

To benchmark ENCODE-rE2G and other models on a separate, held-out CRISPR dataset, we collected and uniformly reprocessed data from 8 CRISPR perturbation screens covering 5 cell types to create a held-out dataset on which to benchmark our models. These datasets included: three Perturb-seq experiments in K562 (Xie *et al.*, 2019[5], Klann *et al.*, 2021[6], Morris *et al.*, 2023[7]); two enhancer screens performed in K562 and WTC11 using direct-capture targeted Perturb-seq (Ray *et al.*, 2025[10]); and element-gene pairs tested with CRISPRi-FlowFISH in K562 (Reilly *et al.*, 2021[11]), HCT116 (Guckelberger *et al.*, 2024[8]), GM12878 (Nasser *et al.*, 2021[9]), and Jurkat cells (Nasser *et al.*, 2021[9]). Below, we describe the processing steps applied to each dataset.

19.1. *Processing of Perturb-seq datasets.* We reprocessed data for all Perturb-seq datasets (Xie *et al.*, 2019[5], Klann *et al.*, 2021[6], Morris *et al.*, 2023[7]) using the methodologies described in the original publications. This included standard preprocessing steps: cell quality filtering based on UMI counts, mitochondrial transcript fraction, and gene detection thresholds specific to each dataset. For each dataset, we generated three core data matrices: (1) an mRNA gene expression counts matrix (genes × cells), (2) a guide RNA counts matrix (guides × cells), and (3) an annotation file describing each guide and its target element. To ensure consistent evaluation across all datasets, we defined a unified set of candidate elements using the ABC pipeline[1] from K562 DNase-seq data (see **Supplementary Methods 5,** ENCODE experiment ENCSR000EOT). Only guides that overlapped with these candidate elements were retained for downstream analysis. The preprocessing details of the raw data for each Perturb-seq dataset is described below.

19.2. *Processing of CRISPRi-Perturb-seq data from STING-seq v1 screen from Morris et al., 2023[7].* We retrieved the data for the STING-seq v1 screen (the smaller pilot screen from the study) from GSE171452, including count matrices for cDNA, cell-hashing (HTO), and guide-barcode (GDO). A Seurat (v5.0.1) object was initialized with the cDNA matrix; HTO and GDO matrices were added as separate assays. Cells were retained if their unique-gene count, total cDNA UMI count and mitochondrial transcript fraction lay between the 15th–99th, 20th–99th and 5th–90th percentiles, respectively. HTO counts were centre-log-ratio normalized and demultiplexed with HTODemux (positive.quantile = 0.99); only singlets were retained. The guide sequences were validated using UCSC's BLAT API, as the modest scale of the guide library permitted API-based validation without server limitations, with requirements for 100% sequence similarity to unique genomic locations. The guides were then overlapped with the K562 candidate elements described above.

19.3. *Processing of CRISPRi-Perturb-seq data from STING-seq v2 screen from Morris et al., 2023[7].* We retrieved the raw data for the STING-seq v2 screen (the larger, full-scale screen from the study) from GSE171452. We performed quality control separately for each lane: cells were retained if their unique-gene count lay between the 1st-99th percentiles, total cDNA UMI count between the 10th–99th percentiles, and mitochondrial

transcript fraction between the 1st–90th percentiles. HTO counts were centre-log-ratio transformed and demultiplexed with HTODemux (positive.quantile = 0.99), retaining singlets. Because this experiment included 188 antibody-tagged oligonucleotides (ADT), the ADT profiles were further filtered to keep cells whose total ADT UMIs were within the 1st–99th percentile of the lane distribution. We found that batch B in the dataset had a dramatically lower distribution of UMIs than any of the other batches, so we removed those cells from further processing, then merged the three lane-specific Seurat (v5.0.1) objects. To validate guide sequences, we established a local BLAT server using the hg38 reference genome (hg38.2bit) with parameters -stepSize=5 for the server and -minScore=20 -minIdentity=0 for client queries, requiring 100% sequence similarity to unique genomic locations. The guides were then overlapped with the K562 candidate elements.

19.4. *Processing of CRISPRi-Perturb-seq data from Xie et al., 2019*[5]*.* We obtained counts files from GEO accession GSE129837. For each batch we constructed a Seurat (v5.0.1) object and removed low-quality cells (unique-gene and total-UMI counts below the 10th percentile or above the 99th, and mitochondrial-read fraction >10%). Guides were filtered by alignment to hg38 using BLAT and overlap with the DNase-seq elements in K562 defined above. We validated guide sequences using the approach described above for STING-seq v2 from Morris *et al.*, 2023[5], then overlapped with the K562 candidate elements. The batch-level objects were merged to yield an mRNA expression matrix, a gRNA count matrix, and a metadata file.

19.5. *Processing of CRISPRi-Perturb-seq data from Klann et al., 2021*[6]*.* We obtained the mRNA count matrix and the corresponding gRNA UMI matrix from ENCODE experiment ENCFF904ZDX. In line with the original analysis, the gene matrix was loaded into Seurat (v5.0.1). Cells with >20 % mitochondrial UMIs or <10,000 total transcript UMIs were discarded, after which a single Seurat (v5.0.1) object was created containing the mRNA counts assay and the gRNA counts assay. Genomic coordinates in hg19 for the guides provided by the authors were lifted to GRCh38 with easylift (v1.0.0), retaining only guides with unambiguous positions. The lifted-over guides were then overlapped with the K562 candidate elements.

19.6. *Analyzing differential expression:* We performed differential expression analysis using SCEPTRE v0.10.0, a tool designed for single-cell CRISPR screen analysis. For each dataset, we tested every gene against all targeted elements within 1 Mb of the gene's transcription start site (TSS), with distances calculated using GENCODE v29 gene annotations. SCEPTRE was configured using the high MOI setting to account for cells containing multiple guides, and a union integration strategy to aggregate effects across multiple guides targeting the same element. We employed two-sided testing to detect both activating and repressing effects, and included dataset-specific batch information as covariates when available. The analysis workflow consisted of guide assignment using SCEPTRE's mixture model approach, followed by quality control filtering using SCEPTRE's default quality control parameters and SCEPTRE's discovery analysis statistical framework using a Benjamini-Hochberg False Discovery Rate (FDR) significance cutoff of 10%.

19.7. *Calculating statistical power for each element-gene pair in Perturb-seq datasets:* To ensure robust negative controls and address potential false negatives due to insufficient statistical power, we adapted the power simulation approach described in **Supplementary Methods 18.1** for SCEPTRE to simulate perturbation effects at multiple

effect sizes to estimate the statistical power of detecting true regulatory interactions. We tested effect sizes of 2%, 3%, 5%, 10%, 15%, 20%, 25%, and 50% decreases in gene expression across 100 simulation replicates per effect size to ensure robust power estimates. The simulations incorporated realistic guide-to-guide variability in knockdown efficiency, a parameter derived from previous experimental observations.

To simulate count data for perturbed and control cells, we used mean expression and dispersion parameters (1/θ where θ is SCEPTRE's overdispersion parameter) for each gene, extracted from the original SCEPTRE analysis. For each cell-gene combination, the simulation used the guide assignments from the original SCEPTRE analysis, then assigned guide-specific effect sizes drawn from normal distributions ($\sigma = 0.13$ based on analysis of previous experiments). For guides targeting the perturbation of interest, effect sizes were sampled from distributions centered on the desired effect size, while control guides received effect sizes centered on no effect. The effect size matrix was then centered so that the average effect across all guides targeting a given perturbation equaled the specified effect size. Using these cell- and guide-specific effect sizes along with gene-specific parameters, synthetic UMI count matrices were generated via negative binomial sampling, followed by application of SCEPTRE differential expression analysis to the simulated data within each replication. For significance testing in the simulations, we used the largest nominal *P*-value that achieved significance after FDR correction in the original differential expression analysis as the threshold for calling effects significant in the simulated data, in order to align with the significance thresholding on the real experiment. Power was computed as the proportion of simulations where both the *P*-value fell below this threshold and the effect size was negative, as all simulated effects were negative and any significant positive effects would be incorrect.

19.8. *Filtering pairs based on genomic location*. To identify element-gene pairs specifically testing distal enhancer-gene relationships rather than direct gene targeting effects, we annotated pairs based on the genomic features overlapping targeted elements. We annotated pairs with genomics features using GENCODE v29 annotation as follows:

Each perturbation element was overlapped with three genomic feature categories. First, we defined promoter regions as 1 kb upstream of the TSS for all annotated transcripts using the GenomicRanges promoters function. Second, we extracted all annotated exons to define gene body regions. Third, we calculated gene loci coordinates by taking the range of all exons per gene to capture complete gene bodies including intronic regions. We then performed overlap analysis between perturbation coordinates and these genomic features using findOverlapPairs, checking specifically whether each element overlapped the promoter or gene body of its target gene.

Element-gene pairs were removed if the targeted element overlapped a promoter region, regardless of the gene for that promoter. Similarly, pairs were removed if the targeted element overlapped any portion of the target gene's gene body, including both exonic and intronic regions, as this might have confounding effects due to RNA polymerase II interference. Only element-gene pairs passing all genomic feature filters were retained in the held-out dataset.

19.9. *Processing of direct-capture targeted Perturb-seq (DC-TAP-seq) datasets from Ray et al., 2025*[10]. We obtained the DC-TAP-seq datasets conducted in K562 and WTC11 from Supplementary Table 3 in Ray et al., 2025[10]. The element-gene pairs had already

undergone SCEPTRE-based differential expression analysis and power simulation in the original study. We used the 'significant' column, which included positive control perturbations in the analysis, to define positive and negative pairs, in combination with the "power_at_effect_size_15" to filter negatives for a minimum of 80% power to detect a 15% effect size. Consistent with the filtering based on genomic location for the other Perturb-seq datasets, we retained pairs classified as "DistalElement_Gene", which the authors defined by removing cases where the tested element overlapped the promoter or gene body of the target gene. We also filtered the results to retain only pairs with distances under 1 Mb to the TSS to align with the distance criteria used for other Perturb-seq datasets.

19.10. *Processing other datasets.* We obtained additional CRISPR enhancer perturbation results from the following published datasets. For these datasets, we used enhancer-gene links and applied statistical power filters, where available, as provided by the analyses of the respective studies.

19.10.1. *Nasser et al., 2021*[9]. We extracted analyzed and statistical power filtered results on enhancer perturbations in GM12878 and Jurkat cells from: https://raw.githubusercontent.com/EngreitzLab/ABC-GWAS-Paper/main/comparePredictorsToCRISPRData/comparisonRuns/AllCellTypes-ABC_comparison/experimentalData/experimentalData.AllCellTypes.txt and reformatted them for our benchmarking framework.

19.10.2. *Guckelberger et al., 2024*[8]. We obtained analyzed results including statistical power analysis on enhancer perturbations in control HCT116 from Supplemental Table 2 and reformatted them for our benchmarking framework.

19.10.3. *Reilly et al., 2021*[11]. We downloaded element-level quantifications for positives from CRISPRi tiling experiments in K562 from the ENCODE portal. We filtered the data based on genomic location to be consistent with the other datasets by removing element-gene pairs in which the element overlaps an annotated promoter or the target gene body (**Supplementary Methods 19.8**), then reformatted them for our benchmarking framework. Because this experiment did not allow for computing % effects on gene expression and because no power analyses were performed by the authors, we only retained significant element-gene pairs from this dataset and excluded pairs that did not have a significant effect.

20. Annotating CRISPR elements with chromatin categories

We annotated the tested elements in training and held-out CRISPR datasets using H3K27ac, H3K27me3, and CTCF epigenetic datasets to characterize the chromatin states of the tested and positive element-gene pairs.

20.1. *Processing input data.* For K562, GM12878, HCT116, and WTC11, we downloaded BAM files and peak calls from the ENCODE portal. We also obtained DNase-seq data for Jurkat from the ENCODE portal. We filtered the BAM files according to ENCODE data processing pipelines depending on the assay and run type (https://www.encodeproject.org/pipelines). For Jurkat, we downloaded FASTQ files from CTCF, H3K27ac, and H3K27me3 ChIP-seq experiments from SRA and prepared filtered BAM files and peak calls according to ENCODE data processing pipelines. All epigenetic datasets used are listed here:

https://github.com/mayasheth/chrom-annotate/blob/main/resources/metadata/epigenetic_datasets.tsv

20.2. *Calculating chromatin/epigenetic features of tested elements.* To analyze the chromatin/epigenetic features of the tested elements, we first determined whether elements overlapped H3K27ac, H3K27me3, or CTCF ChIP-seq peaks in their respective cell types. H3K27ac and H3K27me3 peaks were extended by 175 bp on each side due to the broad distribution of histone ChIP-seq signals. We also computed the H3K27ac RPM in each element expanded by 150 bp on each side. A pipeline implementing these annotations is available here: https://github.com/mayasheth/chrom-annotate

20.3. *Categorizing elements into chromatin categories.* We categorized elements according to the following ordered rules:

```
If element overlaps H3K27ac peak:
     H3K27ac RPM >90% for cell type → High H3K27ac element
     Else →  H3K27ac element
Else if element does not overlap H3K27ac peak:
     H3K27ac RPM >90% for cell type →  H3K27ac high element
     H3K27ac RPM >50% for cell type → H3K27ac element
     Element overlaps CTCF peak → CTCF element
     Element overlaps H3K27me3 peak → H3K27me3 element
     Else → No H3K27ac element
```

21. Calculating direct/indirect effect rates

21.1. *Calculating indirect and direct effects rates in CRISPR perturbation screens.* The observed E-G pairs in CRISPR perturbation experiments contain both direct, cis-acting effects of element perturbations and indirect, trans-acting effects. To calculate the direct effects rate in the used CRISPR datasets, we first empirically estimated the indirect effects rates. We estimated the distance independent indirect effects rates for the K562, WTC11 and the uniformly reprocessed datasets (Xie et al. 2019[5]; Klann et al. 2021[6]; Morris et al. 2023[7]; Gasperini et al. 2019[3]) by systematically testing distal element perturbations for inter-chromosomal trans-acting effects. For each dataset we applied the same differential expression testing approach used to identify E-G pairs, but tested each distal element perturbation against 100 randomly selected genes on other chromosomes. Only genes part of the cis discovery pairs (**Supplementary Methods 18.2, 19.6**) were considered to ensure similar expression distributions of tested genes between cis- and trans-acting analyses. The dataset specific indirect effects rate was calculated as the proportion of positive trans-acting differential expression tests using the uncorrected *P*-value cutoff corresponding to the chosen FDR cutoff in each dataset (**Supplementary Methods 18.2, 19.6, 19.9**). This approach was used to calculate indirect effect rates for both positive and negative effects on expression.

To calculate the direct effects rate for each dataset, first we calculated the observed positive hit rate in the cis discovery analysis as a function of distance to TSS. The *cis* discovery pairs within 1Mb of the TSS were grouped into 50kb bins, and for each bin the positive hit rate was calculated as the proportion of positive tests for both positive and negative effects on expression. To calculate the rate of direct effects, for each distance bin the distance independent indirect effects rate was subtracted from the observed positive hit rate.

We calculated the average direct rate per distance bin across all used datasets and fitted a powerlaw function to model the direct effects rate as function of distance to TSS using the `lm` function in R (4.2.0). Only distance bins with a direct rate greater than zero were included in this analysis. This model was then applied to predict the expected direct effects rate for all cis discovery pairs in all CRISPR datasets based on their distance to TSS.

21.2. *Imputing indirect effect rates.* For CRISPR datasets in both the training and held-out data where we could not empirically estimate an indirect effects rate, we used the average empirical indirect effects rate calculated as described above.

21.3. *Calculating the probability of direct versus indirect CRISPR effects.* The probability of each cis discovery pair being the result of a direct versus an indirect effect was calculated from the expected direct effects rate based on its distance to TSS and the datasets indirect effects rate:

$$p_{direct} = Probability\ direct\ vs.\ indirect\ effect$$
$$r_{direct} = Direct\ effects\ hit\ rate$$
$$r_{indirect} = Indirect\ effects\ hit\ rate$$
$$d = Distance\ to\ TSS$$

$$p_{direct}(d) = \frac{r_{direct}(d)}{r_{direct}(d) + r_{indirect}}$$

This approach was used to calculate probabilities of direct effects for both positive and negative effects on gene expression. All code to compute probabilities of direct effects is available on GitHub: https://github.com/EngreitzLab/CRISPR_indirect_effects.

22. CRISPR benchmark

To benchmark ENCODE-rE2G and other predictive models against CRISPR enhancer perturbation data, we developed a systematic benchmarking pipeline available at: https://github.com/EngreitzLab/CRISPR_comparison. We benchmarked predictive models against both the combined K562 training dataset described above (**Supplementary Methods 18**) and a combined held-out dataset that we generated as follows:

22.1. *Curating combined held-out dataset for benchmarking.* To create the final held-out dataset on which to benchmark models, we combined all of the processed element-gene pairs, resolved any duplicated element-gene pairs tested in the same cell type, and removed pairs that were present in the training dataset. In order to obtain a clean set of negatives, we retained non-significant element-gene pairs that were tested with sufficient power to detect a change in gene expression. To obtain a clean set of positives likely to represent real enhancer interactions, we applied filters based on effect size and chromatin state at the tested element.

22.1.1. *Combining all held-out datasets.* We combined the individual processed and filtered sets of element-gene pairs into a single dataset. We first resized the tested elements from Ray *et al.*, 2025 (generally 300 bp) to 500 bp (+/- 250 bp from center) to be consistent with the rest of the dataset. When duplicate

element-gene pairs existed within the same cell type, pairs were resolved using the following prioritization: (1) if only one pair contained a significant result for the pair, the significant result was retained; (2) if both datasets had the same significance status (both significant or both non-significant), the pair with higher statistical power at 15% effect size was retained.

22.1.2. *Removing pairs present in the training dataset.* To fairly evaluate the performance of the model on an unseen dataset, we removed any element-gene pairs that were already present in the training dataset. To do so, we removed any element-gene pairs from the held-out dataset tested in K562 that overlapped with element-gene pairs in the training dataset.

22.1.3. *Filtering element-gene pairs based on power analysis.* For the Perturb-seq and DC-TAP-seq datasets that had undergone power analysis with SCEPTRE, we retained negative element-gene pairs that 1) were non-significant in the original SCEPTRE analysis and 2) had at least 80% power detect a 15% effect size. We retained positives with an effect size greater than 5%. For the FlowFISH experiments from Nasser *et al.*, 2021 and Guckelberger *et al.*, 2024, we used power analysis results provided by the authors to retain negatives that had at least 80% power to detect a 25% effect size. We retained significant positives with any effect sizes.

22.1.4. *Filtering positives to likely enhancer interactions.* To ensure the positives in the CRISPR datasets represented enhancer-like regulatory mechanisms that the models are intended to predict, we removed positives with elements in the CTCF element, H3K27me3 element, or no H3K27ac categories. H3K27ac is a canonical marker of active enhancers, deposited as a result of transcription factor and cofactor binding that drives enhancer function. Positives lacking H3K27ac signal likely regulate gene expression through alternative mechanisms (such as CTCF-mediated chromatin looping or repressive histone modifications) that fall outside the scope of enhancer-gene linking models. By filtering out these cases, we focused the benchmark on regulatory elements that operate through the mechanisms that enhancer-gene linking models are designed to predict.

22.2. *Summarizing held-out CRISPR dataset.* The final held-out dataset used in the CRISPR benchmark is available https://github.com/EngreitzLab/CRISPR_comparison/blob/dev/resources/crispr_data/EPCrisprBenchmark_combined_data.heldout_5_cell_types.GRCh38.tsv.gz and is summarized as follows:

**Reference**	**Cell type**	**Negatives**	**Positives**	**Weighted positives**
Xie *et al.*, 2019	K562	399	42	37.1
Klann *et al.*, 2021	K562	12	21	18.9
Morris *et al.*, 2023	K562	149	35	29.1
Reilly et al., 2021	K562	0	8	7.4
Ray *et al.*, 2025	K562	1240	12	8.2
Ray *et al.,* 2025	WTC11	1906	15	13.4
Guckelberger *et al.*, 2024	HCT116	362	34	22.6
Nasser *et al.*, 2021	GM12878	52	16	14.3
Nasser *et al.*, 2021	Jurkat	68	7	6.3

22.3. *Benchmarking predictive models against CRISPR data.* To benchmark the performance of predictive models, all E-G pairs from predictions were overlapped with the CRISPR E-G pairs using the following algorithm (for each predictive model separately): For a given gene found in both the CRISPR data and predictions (matched by gene symbol) CRISPR elements were overlapped with elements in predictions based on genomic coordinates. If a CRISPR element overlapped a prediction element, the prediction score was assigned to that CRISPR E-G pair. If more than one prediction element overlapped the CRISPR element, the prediction scores were aggregated using a predictor-specific function (*e.g.*, sum or max, see **Supplementary Table 1**). If no prediction element overlapped a given CRISPR element, this element was considered as "not predicted" by the predictive model and the minimum possible prediction score was assigned to that CRISPR pair. The merged CRISPR and predictions data was then used to compute precision-recall curve metrics (ROCR v. 1.0-11) using the binary experimental outcome whether a CRISPR E-G pair was detected as positive or negative as ground truth.

22.4. *Quantifying model performance and comparing models using bootstrapping.* To quantify and compare model performance, Area Under the Precision-Recall Curve (AUPRC) and precision or recall at 70% recall, or at the chosen threshold, were computed (**Supplementary Table 3**). A bootstrapping approach was applied to estimate 95% confidence intervals by re-sampling with replacement (10,000 iterations) and computing the range containing 95% of bootstrapped values. When bootstrapping precision at 70% recall, the metric was defined as precision at the predetermined threshold yielding 70% recall, rather than the precision closest to 70% recall in each sample. The R package boot (v. 1.3-28.1) was used to perform bootstrap sampling. To assess the significance of pairwise comparisons in performance between models, the delta AUPRC and delta precision or recall at 70% recall, or at the chosen threshold, were computed. For significance between benchmarking datasets, delta performance metrics were computed between bootstrap samples from each dataset per iteration. The same bootstrap scheme as described above with 10,000 iterations was used to obtain 95% confidence intervals and the approach to compute two-sided *P*-values through inversion of confidence intervals implemented in the boot.pval (v. 0.7.0) R package was applied. All bootstrapping functions are available as part of the CRISPR benchmarking pipeline: https://github.com/EngreitzLab/CRISPR_comparison/blob/main/bootstrap_analysis_examples.Rmd

22.5. *Computing weighted performance metrics.* To take into account the uncertainty of indirect CRISPR perturbation effects when benchmarking enhancer-gene prediction models, we computed precision-recall curves where each CRISPR element-gene pair was weighted by its probability to be a direct effect. Since enhancer-gene linking models are designed to predict direct regulatory relationships, weighting by the probability of direct effects ensures that our benchmark prioritizes true enhancer-gene connections while down-weighting confounding indirect effects that may artificially inflate or deflate model performance. Weighted precision-recall curves were computed using the pr_curve function from the yardstick (v=1.2.0) R package with case weights for each CRISPR E-G pair set to the probability of a direct effect.

23. GWAS benchmark

We developed a GWAS benchmarking pipeline to assess whether predictive models of enhancer-gene regulatory interactions could (i) identify enhancers enriched for fine-mapped GWAS variants, and (ii) link those enhancers to genes already known to be involved in the GWAS disease/trait, similar to our previous approach[9] (**Supplementary Table 7**). For this benchmarking pipeline, we analyzed data from the UK Biobank for 94 traits (**Supplementary Table 6**).

23.1. *Fine-mapped GWAS variants.* We obtained fine-mapping results and summary statistics on 94 traits based on an unpublished analysis (J.C.U., M. Kanai and H.K.F., unpublished data) that analyzed data from the UK Biobank (application 31063; fine-mapping data are available at https://www.finucanelab.org/data). In this analysis, up to 361,194 individuals of white British ancestry with available phenotypes and variants with INFO > 0.8, minor allele frequency > 0.01%, and Hardy–Weinberg equilibrium P > 1 × 10−10 were included in the GWAS. Covariates for the top 20 principal components, sex, age, age^2, sex × age, sex × age^2 and dilution factor, where applicable, were controlled for in the association studies. Quantitative traits were inverse rank transformed and associations were estimated using BOLT-LMM for quantitative traits and SAIGE for binary traits. In-sample dosage linkage disequilibrium was computed using LDStore, and phenotypic variance was computed empirically. Fine-mapping was performed using the sum of single effects (SuSiE) method, allowing for up to ten causal variants in each region. Prior variance and residual variance were estimated using the default options, and single effects (potential 95% credible sets) were pruned using the standard purity filter such that no pair of variants in a credible set could have r^2 > 0.25. Regions were defined for each trait as ±1.5 Mb around the most significantly associated variant (with this window chosen based on the linkage disequilibrium structure in the human population), and overlapping regions were merged. Variants in the MHC region (chr. 6: 25–36 Mb) were excluded as were 95% credible sets containing variants with fewer than 100 minor allele counts. Coding (missense and predicted loss of function) variants were annotated using the variant effect predictor v.85.

For all traits, except where specified, we considered only the 'noncoding credible sets'—that is, those that did not contain any variant in a coding sequence or within 10 bp of a splice site annotated in the RefGene database (downloaded from UCSC Genome Browser on 24 June 2017)

23.2. *Calculating enrichment of GWAS variants in merged enhancer elements.* We filtered for enhancers where Score > threshold (**Supplementary Table 1**) for downstream enrichment analysis. For a given trait, we intersected variants with PIP > 10% in

noncoding credible sets with enhancers (or other genomic annotations). We merged enhancer regions across cell types to generate a unique set of enhancer regions for each predictor. We then calculated enrichment values by looking at the number of variants with PIP > 10% that overlapped the merged enhancer set over the total number of background variants, defined by common and low frequency variants (Minor allele count >=5) in 1000 Genomes Project, that overlapped enhancers. For calculating enrichment values excluding promoter regions, we performed the same calculations but only considered enhancers that did not overlap promoter regions. We report these metrics in **Fig. 3b**.

23.3. *Disease Heritability enrichment using Stratified LD score regression.* Stratified LD score regression (S-LDSC) is a method that assesses the contribution of a genomic annotation to disease and complex trait heritability[54,55]. S-LDSC assumes that the per-SNP heritability or variance of effect size (of standardized genotype on trait) of each SNP is equal to a linear contribution of each annotation. $var(\beta_j) = \sum_j a_{cj} \tau(c)$, where a_{cj} is the value of annotation c for SNP j, and $\tau(c)$ is the contribution of annotation c to per-SNP heritability conditioned on other annotations. S-LDSC estimates the $\tau(c)$ for each annotation using the following equation:

$$E(\chi^2_j) = N \sum_c l(j, c)\, \tau(c) + 1$$

where $l(j, c) = \sum_k a_{ck} r^2_{jk}$ is the stratified LD score of SNP j with respect to annotation c and r_{jk} is the genotypic correlation between SNPs j and k computed using data from 1000 Genomes Project (see URLs); N is the GWAS sample size. We assess the informativeness of an annotation c using two metrics. The first metric is enrichment (E), defined as follows (for binary and probabilistic annotations only):

$$E = h^2_g(c) / h^2_g \times M / \sum_j a_{cj}$$

where $h^2_g(c)$ is the heritability explained by the SNPs in annotation c, weighted by the annotation values. The second metric is standardized effect size (τ^*) defined as follows:

$$\tau^*(c) = \tau(c)\, sd_c / (h^2_g / M)$$

where sd_c is the standard error of annotation c, h^2_g is the total SNP heritability and M is the total number of SNPs on which this heritability is computed (equal to 5,961,159 in our analyses). $\tau^*(c)$ represents the proportionate change in per-SNP heritability associated to a 1 standard deviation increase in the value of the annotation.

23.4. *Precision-recall analysis of silver-standard causal genes in GWAS loci.* We evaluated the accuracy of linking noncoding GWAS variants to target genes using a strategy we previously described[56], in which we attempt to link noncoding GWAS signals to nearby genes that carry coding variants independently associated with the same trait. The genes carrying coding variants are very likely relevant to the trait, and are more likely than other genes in the same locus to be the target of the independent noncoding

variant[56]. Using fine-mapping for UK Biobank traits as described above, we defined a set of 560 noncoding credible sets (comprised entirely of noncoding variants) corresponding to 31 UKBiobank traits that are located near exactly 1 gene carrying a coding variant, similar to the approach we previously described[56]. First, we used the Variant Effect Predictor[57] to identify protein-truncating variants and damaging missense variants with posterior inclusion probability from fine-mapping (PIP) >= 50%. Second, we examined noncoding credible sets in which exactly 1 gene within 2 Mb carried such a coding variant. Third, we removed duplicated credible sets in which the same noncoding variant was associated with multiple traits (*e.g.*, due to genetic correlation between the traits). We deduplicated such cases by (i) finding 'duplicate' variant-gene pairs where the same noncoding variant had PIP >= 0.05 for more than two traits, and was in the same locus attempting to link to the same gene with a coding variant; and (ii) removing such duplicates, keeping the credible set where the variant had a higher PIP. For the primary benchmarking analysis in **Fig. 2g**, we restricted our analysis to 197 out of the 560 noncoding credible sets corresponding to 11 blood-related traits (Eosinophil count, Lymphocyte count, Monocyte count, Neutrophil count, Platelet count, RBC count, Hemoglobin count, Mean Corpuscular Hemoglobin, Mean Corpuscular Hemoglobin concentration, Mean Corpuscular Volume, All Autoimmune Disease - UKBB). This choice was motivated by the fact that immune cell types had a much more consistent and elaborate representation across different enhancer-gene strategies compared to other cell types. We use the same benchmarking data for the analysis reported in **Fig. 3c**. Results for all 560 noncoding credible sets are reported in **Extended Data Fig. 5**.

23.5. Enrichment of variants with ENCODE-rE2G ∩ PoPS predictions. We illustrate the strategy adopted for calculating the enrichment of biosamples against GWAS traits based on the combined ENCODE-rE2G ∩ PoPS score. First, we map each of n = 9.2 million common and low-frequency (minor allele count >=5) variants in 1000 Genomes Project to the top two PoPS genes for a focal trait within a 1Mb genomic window around that variant. Second, for variants that overlap a predicted enhancer by ENCODE-rE2G, we identify the top two genes based on the continuous predictive score in that biosample. Next, we identified N_b variants for which there is overlap among the top two genes from PoPS score for a specific trait and ENCODE-rE2G score for a specific biosample, and compute the baseline fraction of ENCODE-rE2G+PoPS variants genome-wide as $f_{base} = N_b / N$. Next, we compute the similar fraction $f_{finemap}$ by restricting our variant set to fine-mapped variants (PIP> 0.1) for the focal trait, and finally we calculate the enrichment as $f_{finemap} / f_{base}$.

24. eQTL benchmark

We developed an "eQTL benchmarking pipeline" to assess whether predictive models of enhancer-gene regulatory interactions could (i) identify enhancers enriched for fine-mapped eQTL variants, and (ii) link those enhancers to their corresponding eQTL target genes. We analyzed variants from the GTEx V8 resource[26] fine-mapped using the Sum of Single Effects (SuSIE) model[58]. The pipeline is implemented here: https://github.com/EngreitzLab/eQTLEnrichment/blob/integrated.

24.1. Fine-mapped eQTL variants. We obtained the set of GTEx V8 variants fine-mapped using SuSiE[58] by Hilary Finucane and Jacob Ulirsch at the Broad Institute. We considered variants with a posterior inclusion probability (PIP) greater than 50% and that were included in a credible set. We converted the target genes for each variant from

their Ensembl ID to HGNC symbol, removing variants without a corresponding HGNC symbol. We mapped the median transcripts per million (TPM), obtained from GTEx (https://storage.googleapis.com/gtex_analysis_v8/rna_seq_data/GTEx_Analysis_2017-06-05_v8_RNASeQCv1.1.9_gene_median_tpm.gct.gz), to each variant depending on the target gene and tissue it was identified in. We included variants linked to genes expressed at levels above 1 TPM from their respective tissues. For each predictive model, GTEx variants and enhancer-gene links were filtered to those linked to genes in their shared gene universe. The GTEx variants and TPM file are available to download on Synapse at the following link: https://www.synapse.org/Synapse:syn70198274.

24.2. *Filtering to distal noncoding variants.* For eQTL benchmarking analyses, we focused on "distal noncoding variants by filtering out any variants overlapping (i) coding sequences, 5′ and 3′ untranslated regions of protein-coding genes, (ii) splice sites (within 10 bp of an intron–exon junction of a protein-coding gene) of protein-coding genes, and (iii) promoters (±250 bp from the gene TSS) of protein-coding genes.

24.3. *Defining tissue - biosample matches for eQTL benchmarking.* For each predictive model that was applied to multiple biosamples, we assigned each biosample to any matching GTEx tissues (*e.g.*, GTEx tissue "Prostate" was paired with DNase experiments "prostate_gland_ENCSR564FZH" and "epithelial_cell_of_prostate_ENCSR000EPU" to benchmark ENCODE-rE2G and ABC). The set of 13 tissues represented by all predictive models was used for the benchmarking analyses in **Extended Data Fig. 4c,d**. The biosamples matching each of the 13 GTEx tissues for each predictive model are listed in **Supplementary Table 5**. To generate enrichment-recall curves (**Fig. 2d**, **Extended Data Fig. 4b**), the GTEx tissue "EBV-transformed lymphocytes" was paired with enhancer-gene predictions in a particular lymphoblastoid cell line, GM12878.

24.4. *Calculating enrichment of eQTLs in predicted enhancers.* We defined enrichment as (fraction of distal noncoding eQTL variants with PIP >= 50% that overlap predicted enhancers) / (fraction of distal noncoding 1000G SNPs that overlap predicted enhancers) (see **Fig. 2e** and **Extended Data Fig. 4b,c,d**). To calculate this enrichment, we obtained a list of approximately 10 million SNPs from the 1000 Genomes Project from the Price Group (https://alkesgroup.broadinstitute.org/LDSCORE/baseline_v1.1_hg38_annots/), then filtered them to the distal noncoding regions of the genome as described above. For each predictive method, we further filtered the GTEx variants to those with an eGene in the gene universe considered by that predictive method; in absence of a provided gene universe file, we used our curated gene annotations (see **Supplementary Methods 2**). We then calculated the number of variants from each GTEx tissue. Next, we thresholded the predictions for each method based on the threshold that yielded 70% recall from the CRISPR benchmark, and intersected the predictions from each biosample with the GTEx variants from each tissue, recording the number of variants at each intersection. We also calculated the number of distal noncoding SNPs intersecting thresholded predictions in each biosample. The enrichment for each prediction biosample, B and GTEx tissue, T intersection was then calculated as [number of variants in T overlapping predicted enhancers in B / total number of variants in T]/[number of distal noncoding 1000G SNPs overlapping B / total number of distal noncoding 1000G SNPs]. We used a modified enrichment calculation for Enformer: because the predicted enhancers for Enformer were generated based on a control set of DNase peaks overlapping a random set of 10,000 1000G SNPs, the enrichment calculation was modified for by defining the background set of distal, noncoding variants as this list of 10,000 variants.

24.5. *Calculating recall of predictive models at linking eQTL variants to target eGenes.* To calculate the recall of prediction methods in linking an eQTL variant to its eGene, GTEx tissues were matched with biosamples from each prediction method to define corresponding pairs (**Supplementary Table 5**). For each pair, we calculated the 1) total recall, defined as the fraction of eQTL variants overlapping predicted enhancers (**Extended Data Fig. 4c,d**) 2) recall accounting for gene linking, defined as the fraction of eQTL variants overlapping predicted enhancers that are linked to the variant's eGene (**Fig. 2e**, **Extended Data Fig. 4b**); and 3) the fraction of variants overlapping a predicted enhancer linked to the variant's eGene, given that the variant overlaps a predicted enhancer.

24.6. *Choosing threshold values for enrichment-recall curves.* We calculated a range of predictor score values to construct enrichment-recall curves (**Fig. 2e**, **Extended Data Fig. 4b**) by defining 50 to 100 quantiles across the combined recall of matching tissues and biosamples for each predictive model. We took the unique set of variants overlapping predicted enhancers where the variant's eGene was linked to the enhancer's target gene, then calculated the score that yielded each interval of the total possible recall. Enrichment and recall were evaluated at each of the threshold values to generate enrichment-recall curves (**Supplementary Table 5**).

25. Benchmarking limitations

We note that each type of benchmarking dataset has its advantages and limitations: (i) CRISPR perturbation experiments characterize cell type specific effects of inhibiting elements on gene expression, providing direct links between enhancers and genes. However comprehensive CRISPR enhancer screens are limited to few cell types, and might contain regulatory interactions likely from indirect effects and, depending on experimental design, from mechanisms other than enhancers (**Supplementary Note 1**)[1] (ii) Fine-mapped eQTL data are available in many primary cell types, but contain uncertainty in identifying causal variants, and can act through other mechanisms beyond perturbing enhancers[26], leading to low absolute recall in our eQTL benchmarks. (iii) Interpreting GWAS signals is a key application of these models, but for the purposes of benchmarking models of enhancer-gene regulatory interactions, there is uncertainty in causal variants, causal cell types, and the "known" genes annotated by independent coding variants[56]. However, assessing predictive models on the three complementary benchmarks across multiple cell types allows comparing the performance on three different orthogonal predictive tasks.

26. Additional analyses: Assaying enhancer activity

To build models for different enhancer activity measurements, element-gene pairs based on candidate elements within 5Mb of candidate promoters were used as the universe of enhancers and genes. Data for 523 1D chromatin experiments (**Supplementary Table 17**) in K562 were downloaded from ENCODE portal as "read-depth normalized signal" (DNase-seq) or "fold change over control" (ATAC-seq, ChIP-seq) bigWig files. Enhancer activities for all candidate elements were calculated for each assay separately using the ABC `run.neighborhoods.py` script, where the different assays were used in place of DNase-seq. The activities for each element and assay were measured by taking the normalized read counts for a given assay. ABC scores for all element-gene pairs and activity measurements were then computed by multiplying activity with the K562 ENCODE Hi-C contact frequencies between candidate elements and promoters as implemented in ABC[1]. Code to compute ABC models using different chromatin assays is available on GitHub: https://github.com/argschwind/ENCODE_eg_enhancer_activity_ABC.

27. Additional analyses: Linking variants to genes analyses

To generate the ENCODE-rE2G ∩ PoPS prediction, we intersected the top two genes with the strongest ENCODE-rE2G prediction score for a peak overlapping a common or low-frequency variant from the 1000 Genomes Project with top two genes in a 1Mb locus around the variant that have the highest Polygenic Priority Score (PoPS)[56]. The PoPS score prioritizes genes for a disease based on their functional similarity to genes implicated by disease GWAS. PoPS trains a model to predict MAGMA (v1.08)[59] gene scores using 57,543 gene-level functional features based on gene expression data (bulk and single-cell RNA-seq), biological pathways, and PPI networks. PoPS uses a leave-one-chromosome-out framework, such that the PoPS score of a gene on chromosome x is not informed by GWAS data or the ENCODE-rE2G linking data on chromosome x. The ENCODE-rE2G ∩ PoPS score, unlike ENCODE-rE2G, is therefore a disease-specific score. For **Fig. 3b**, we assessed the enrichment of the number of disease-related fine-mapped variants (PIP > 0.1) in ENCODE-rE2G ∩ PoPS implicated variants, in comparison to all annotated variants, and also compared precision and recall against silver standard causal genes in the GWAS loci (see GWAS benchmark section above). For **Fig. 3c**, ENCODE-rE2G ∩ PoPS was computed for each of 352 cell-types and 76 diseases and traits using top two ENCODE-rE2G predicted genes at the locus *for each cell-type/biosample*, and intersecting that with top two PoPS prioritized genes. For OpenTargets, we curated the recommended L2G > 0.5 maps corresponding to credible sets linked 1,512 study IDs matching the traits (**Supplementary Table 10**). For OpenTargets (L2G) + PoPs, we intersected L2G > 0.5 linked gene for GWAS credible set locus with the top two PoPs prioritized genes in a 1Mb window around the credible set.

28. Additional analyses: Combinatorial enhancer perturbations at *MYC* locus

28.1. MYC enhancer paired-sgRNA (pgMYC) library design. We conducted a CRISPRi-FlowFISH screen to perturb all pairs of 7 enhancers that we previously found to regulate *MYC* in K562 cells[2] (**Fig. 5d-g**). A lentiviral paired sgRNA vector was used to clone the *MYC* enhancer paired-sgRNA library, such that each vector expresses two sgRNAs driven by either the hU6 or mU6 promoter. Seven previously characterized *MYC* enhancers (e1-7), the *MYC* TSS, two constituent cCREs adjacent to e6 and e7, and a nearby negative control DHS peak (NS1) were selected as target cCREs in this experiment[2].

We designed a lentiviral guideRNA library containing 10,080 pairs of sgRNAs (ENCFF118BUP)**.** For significant CREs characterized in the previous *MYC* enhancer screen[2], we first selected two sgRNAs that had been used in low-throughput enhancer CRISPRi and H3K27ac ChIP-seq assays, then selected six other sgRNAs with the greatest average effect across the previous *MYC* enhancer screen's replicates. For NS1, we selected the two sgRNAs that had been used in low-throughput enhancer CRISPRi and H3K27ac ChIP-seq assays, then selected other sgRNAs by first sorting all GuideScan sgRNAs in the region with CFD specificity scores greater than or equal to 0.2 and efficiency scores greater than or equal to 50, then selecting six sgRNAs such that their rank-ordered genomic positions are evenly spaced apart (six sgRNAs are selected in an every n^{th} fashion after ordering them according to their PAM site's genomic position). For the two constituent cCREs adjacent to e6 and e7, we selected eight sgRNAs using only the GuideScan filters and every n^{th} sgRNA approach, as above. Finally, we included 12 sgRNAs targeting genomic "Safe" harbor regions that lack epigenetic annotations associated with active regulatory elements[60].

These 100 targeting sgRNAs were paired in an all-by-all fashion, such that each sgRNA is paired with every sgRNA (including itself) in both orientations (sgRNA1-sgRNA2 and sgRNA2-sgRNA1). Since recombination between sgRNAs can occur at some frequency due to either lentiviral recombination or PCR-based recombination, we included 80 additional pgRNA vectors in which 5 new "Safe"-targeting sgRNAs that were not among the other 12 "Safe"-targeting sgRNAs were paired with all eight NS1 negative control sgRNAs in both orientations. This allows us to empirically calibrate the recombination rate, by detecting the frequency with which the 5 new "Safe"-targeting sgRNAs are paired with any of the 92 non-NS1-targeting sgRNAs. The entire library consists of 10,080 paired sgRNA vectors.

28.2. *MYC enhancer paired-sgRNA screen.* The pgMYC lentiviral library was synthesized first as an oligo pool from Twist Bioscience, then cloned into a paired sgRNA vector that constitutively expresses the selectable marker Puromycin. Each oligo encodes a pair of sgRNAs, which is first cloned into a vector with a single hU6 promoter, and Puromycin, which is constitutively expressed under the Ef1a promoter. In a second cloning step, a cut site between the pair of sgRNAs linearizes the vector, into which we insert the sgRNA stem loop sequence for the upstream sgRNA, and the mU6 promoter for the downstream sgRNA. The lentiviral plasmid library was transfected into HEK293T cells (purchased from ATCC) to generate lentivirus, which was then transduced into two separate biological replicates of 55 million K562 doxycycline-inducible dCas9-KRAB cells each at an MOI of 0.1 (>500X coverage per replicate). K562 cells were purchased from ATCC and previously engineered to express KRAB-dCas9[1]. 48 hours after transduction, cells were selected for 96 hours with 1.0 ug/mL Puromycin, until a non-transduced pool of the same cells consisted of over 99% dead cells. Cells were recovered in Puromycin-free media and cultured in the absence of doxycycline for an additional week to grow to scale. Throughout their culture, the K562 cells are split back each day to a maximum of 500,000 cells/mL. 100 million cells per replicate were induced with doxycycline at 1 ug/mL for 48 hours prior to harvesting them for FlowFISH. Samples from each biological replicate were split into seven technical staining replicates of 5 million cells, and processed for FlowFISH using probesets for *MYC* and *RPL13A* as previously described[1] (**Extended Data Fig. 8**).

28.3. *Sorting and cytometric analysis.* We diluted the stained cells in PBS with 0.5% BSA to a concentration of 2 × 107 ml–1 and filtered using 35-μm filter tubes (Falcon, no. 352235). We sorted 5 million cells for each replicate into six bins based on the fluorescence intensity of target genes, using the Stanford FACS Core's Influx Sorter (BD Influx, Special Order). Prior to sorting, measurements of the fold-change (FC) for each probeset between a control unprobed sample and each replicate were made to ensure that each of the samples were adequately probed and amplified to a FC >2. To control for differences in staining efficiency for each cell, we normalized the fluorescence associated with the gene of interest to that of the control gene (*RPL13A*) as previously described[1]. We set the gates for each bin on the compensated signal to capture 10% of the cells according to the percentiles (1) 0–10%, (2) 10–20%, (3) 35–45%, (4) 55–65%, (5) 80–90% and (6) 90–100% (**Extended Data Fig. 8**).

28.4. *DNA isolation.* We collected the sorted cells by centrifugation at 800*g* for 5 min, resuspended them in 100 μl of lysis buffer (50 mM Tris-HCl, pH 8.1, 10 mM EDTA, 1% SDS) and incubated them at 65 °C for 10 min for reverse cross-linking. We then added 10 μl Proteinase K (NEB, no. P8107S), mixed well and incubated the mixture at 37 °C for 2 h followed by incubation at 95 °C for 20 min. We extracted genomic DNA using

AMPure XP (SPRI) beads (Beckman Coulter, no. A63882) at 0.7 beads:cell lysate concentration.

28.5. *MYC dual guide screen library preparation and sequencing.* The integrated sgRNAs were amplified using custom primers compatible with Illumina sequencing: AATGATACGGCGACCACCGAGATCTACAC-NNNNNNNNNN-CGTCCGCGGCTTACCGTAACTTGAAAGTATTTCGATTTCTTGGC (where NNNNNNNNNN denotes i5 10bp 96-well barcode) and CAAGCAGAAGACGGCATACGAGATAGACAGCAGTCCCGTGTTCCGGTTCATTCTATCA-NNNNNN-GGATCCCCTAGGAAAAAAAGCACCG (where NNNNNN denotes i7 6bp replicate barcode)**.** PCR conditions: initial denaturation 98 °C for 30 s, 25 cycles of 98 °C for 30 s, 60 °C for 30 s and 72 °C for 2.5 min; and final amplification 72 °C for 5 min. The samples were pooled and purified with AMPure XP beads at a 1:1 beads:PCR product ratio. Sequencing was performed using an Illumina NextSeq with custom primers: Read 1, 25 bases - to read the sequence of Guide 1 (custom primer: CGATTTCTTGGCTTTATATATCTTGTGGAAAGGACGAAACACCG); Read 2, 20 bases - to read 6bp i7 index (custom primer: AGACAGCAGTCCCGTGTTCCGGTTCATTCTATCA); Index 1, 20 bases - to read the sequence of Guide 2 (custom primer: GTAATTGTGTGTTTTGAGACTATAAGTATCCCTTGGAGAACCACCTTGTTGG); Index 2, 10 bases - to read i5 10bp sample barcode (custom primer: ATCGAAATACTTTCAAGTTACGGTAAGCCCGCGGACG).

28.6. *CRISPRi-FlowFISH data processing.* For each sorted bin per sample, reads were aligned to an index of our guide spacers using Bowtie 2[61]. The abundance of each sgRNA pair was quantified using Samtools[62]. These count data were subjected to our previously described pipeline, in which we use the frequency of sgRNA counts in each of the sorted bins to estimate the mean fluorescence value for each sgRNA[1]. Briefly, the mean log10 fluorescent intensity of each sgRNA pair was computed using maximum likelihood estimation fitted to the normalized counts of each sgRNA in the fluorescently sorted bins. Effect sizes for each sgRNA pair were computed by dividing the means of each sgRNA pair by the means of the negative control sgRNAs. We then aggregated information across multiple gRNA pairs to estimate the effect size for perturbing each element or combination of elements. Followed our previous procedure, we adjust the FlowFISH effect size estimates to account for constant background signal that appears to result from nonspecific binding of the probeset[1]; here, we scaled the data using the observed qPCR estimated effects at E2 from our prior study[1] and comparing them to the E2 effects we saw in this CRISPRi experiment (1.57-fold correction). To calculate effect sizes for each element individually, we analyzed guide pairs in which enhancer targeting sgRNAs are paired to negative control sgRNAs. Leveraging this, we computed the effect sizes when targeting a single element and then subsetted our data by the 2 sgRNAs that had the greatest effects when configured with a negative control for every element to refine our dataset to guides of the highest quality. Further quality control was implemented by only including samples where at least 200 cells were observed for a given guide pair across a given sorted sample. From this subsetted data, we re-computed the effect sizes for every guide pair now including the guide pairs that target multiple elements. Each of the sgRNAs' mean effect sizes were computed for every technical replicate and aggregated by target. We performed a Student's two sided, one sample *t*-test for significance testing where each of the technical replicates' estimation of the effect of perturbing a given element pair is considered one data point.

28.7. *Modeling enhancer-gene regulation MYC data.* We compared the observed data to two null models: one where enhancers combine independently and additively in linear expression space (“additive model”), and one where they combine independently and multiplicatively in linear expression space (“multiplicative model”, or additive in log space). The individual effects of each element were estimated as the average of all pairs that include one sgRNA targeting a given element and one negative control sgRNA. To compute the expected effect of perturbing a pair of elements under an additive model, we combinatorially summed the mean effects (% decrease in expression) of each of the two individual elements, and computed 95% confidence intervals by first computing the standard error of the mean of the effect of each element across replicate FlowFISH experiments, and then combining these estimates across the two individual elements using the variance sum law.

We repeat this same analysis for a multiplicative model where the null is the product of the independent effects of each element (*Effect of Enhancer 1 (E1) * Effect of Enhancer 2 (E2) = Expected dual perturbation effect*).

28.8. *MYC dual guide screen statistical analysis.* To build an estimate of the effect that each of our perturbations have on gene expression, we took the average effect across all relevant guide pairs for each element pair perturbation within each FlowFISH technical replicate.The mean of these FlowFISH replicate estimates represents our estimated effect for a given element pair perturbation. We then combinatorially added individual element perturbation effects to build a matrix of expected effect sizes under an additive model. To determine whether there are any interaction effects between enhancer pairs, we performed a *t*-test using a linear model of enhancer interactions:

*Effect on expression = Effect of Enhancer 1 (E1) + Effect of Enhancer 2 (E2) + E1*E2*

where the expected paired effect is the sum of the independent single-enhancer effects. The interaction term captures the deviation of the observed dual-perturbation effect from this additive expectation. Perturbation of a single enhancer disrupts any cooperative interaction with its pair; the interaction term is therefore derived as the observed dual-perturbation effect minus the sum of the two single-perturbation effects. A two-sided *t*-test on the interaction term determined whether the dual-perturbation effect size differed significantly from the additive prediction. *P*-values were adjusted using the Benjamini-Hochberg procedure.

29. Additional analyses: Enhancer synergy analyses

29.1. *Analysis of CRISPRi tiling experiments.* We analyzed comprehensive tiling CRISPRi experiments to determine whether the sum of the effects of enhancers for a given gene would add up to more than 100% (**Fig. 5a**). To do so, we considered a total of 20 CRISPRi experiments where all candidate elements (DNase peaks) around a given gene were perturbed and the effect on gene expression was measured: PPIF in 6 cell types or conditions[9], HBE1 in K562[63], and 13 other genes in K562[1,2], all of which were previously aggregated[9] and available from https://github.com/engreitzlab/abc-gwas-paper. Enhancers were filtered to those with a negative and significant (P_{adj} < 0.05) effect on gene expressions and to those not overlapping promoters, except for two regions overlapping the lncRNA *PVT1* promoter that were previously shown to act as enhancers to regulate *MYC*[2].

29.2. Chromatin immunoprecipitation for H3K27ac following CRISPRi perturbations to MYC enhancers. We conducted experiments to measure the effects of enhancer perturbations on H3K27ac signals at nearby enhancers (**Fig. 5g, Extended Data Fig. 9 a,b**). We selected all 7 enhancers that we previously identified to regulate *MYC* in K562 cells [e1-e7 from Fulco *et al.*, 2016[2]], as well as one additional element in the same locus that did not regulate *MYC* (NS1 from Fulco *et al.*, 2016[2]). We perturbed these enhancers with CRISPRi and conducted ChIP-seq for H3K27ac as previously described[64]. Briefly, we stably delivered individual gRNAs by lentivirus into CRISPRi K562 cells (1 gRNAs per element, as well as 4 negative control gRNAs, see Fulco *et al.* 2016[2]), activated CRISPRi with doxycycline for 48 hours, and then harvested cells for formaldehyde-crosslinked H3K27ac ChIP-seq. We performed 2 replicate ChIP-seq experiments on each of 2 biological replicates per guideRNA (3-4 ChIP-seq experiments per guideRNA, yielding 3-4 experiments per element and 16 experiments including negative control guideRNAs). We aligned and processed these data in genome build hg19 as previously described[9]. We computed a fold-change in H3K27ac reads per million at each ABC candidate enhancer in K562 as defined in Nasser *et al.*, 2021[9] by comparing the 3-4 experiments per element to the 16 negative control experiments.

29.3. Analysis of CRISPRi-H3K27ac ChIP-seq from Fuentes et al. We analyzed data from a previous study in which CRISPRi was directed simultaneously to hundreds of long terminal repeats (LTRs) in the NCCIT cell line using the CARGO system, followed by H3K27ac ChIP-seq to detect changes in enhancer activity[65] (**Fig. 5g**). We used this data to assess how perturbations to enhancers affect H3K27ac ChIP-seq signal at other nearby enhancers. Data from Fuentes et al. was processed similarly to our previous analysis[1]. We first identified enhancer elements by calling peaks from ATAC-Seq data generated in the NCCIT cell line. Each peak was resized to be approximately 500bp centered on the peak summit. For each of these elements we computed the log fold change of H3K27ac ChIP-Seq signal in the wild-type and CRISPRi conditions. We next identified regions potentially targeted by CRISPRi as previously described[1]. Briefly, we first identified LTR regions as the union of DNA elements annotated as either LTR5HS, LTR5A or LTR5B repeats in version 4.0.5 of the RepeatMasker database or as peaks called from dCas9 ChIP-Seq performed by Fuentes et al. This resulted in 1427 candidate LTR regions. Given that these LTR regions may have high sequence similarity, we only considered LTR regions that were sufficiently uniquely mappable. As described in Fulco *et al.* 2019[1], we implemented a simulation approach to assess the mappability of these regions and only considered LTR regions which had >95% uniquely mapping simulated reads. We also only considered LTR regions which were at least 250kb away from any other LTR region. This resulted in 625 LTR regions. We analyzed the fold change in H3K27ac signal for each ATAC-Seq peak within 2mb of an LTR for a total of 136,713 peak-LTR pairs.

29.4. Analysis of H3K27ac chromatin-QTLs from Delaneau et al. *(2019)*. We analyzed H3K27ac chromatin-QTLs[66] to determine how a variant that affects H3K27ac signal in an overlapping peak can affect H3K27ac signal at other nearby peaks (**Extended Data Fig. 9c**). We downloaded data from https://zenodo.org/record/2572871#.Y_P5G-zMK3I. We considered chromatin-QTLs affecting both the H3K27ac peak the variant is located in, and at least one other H3K27ac peak, where the self-peak is less than 5kb in length and where the distance between the self-peak and other peak is greater than 1kb. The relative effect size was defined as negative beta for the nearby peak divided by beta of the self-peak.

29.5. *Analysis of H3K27ac ChIP-seq from Huang* et al. *(2018).* We analyzed data from a previous study in which individual enhancers were genetically deleted with CRISPR followed by ChIP-seq data of H3K27ac[67] (**Fig. 5h**). Data in bigwig format was downloaded from NCBI GEO (GSE107726) and H3K27ac RPMs for control and perturbed regions were computed directly from the bigwig files. RPMs were then averaged across two replicates. We analyzed perturbation-element pairs separated by greater than 1kb, and calculated the fold-change in H3K27ac RPM at the element after perturbation as log2 (H3K27ac RPM after knockout) / (H3K27ac RPM control).

30. Additional analyses: Enhancer-promoter correlation

We conducted an analysis to determine whether eQTL variant-gene pairs were enriched for having high GLS Coefficients (DNase-DNase correlations, see **Supplementary Methods 7**) compared to control variant-gene pairs (**Supplementary Fig. N3.1d**). To compare GLS coefficients with eQTL data, we downloaded the GTEx lymphoblastoid cell line eQTL data from the EMBL/EBI eQTL Catalogue[68]. We set a threshold on the GM12878 DNase-seq data of 1.5 to consider a particular ENCODE cCRE active in GM12878, and kept the eQTL variants that lie within these cCREs but do not overlap the promoters and exons of protein-coding genes. We considered eQTL variants with PIP > 0.05 (n = 1,083 eQTLs). To compare eQTLs to a control set of variants, we selected non-significant eQTLs (P > 0.5) that connect active enhancers and nearby genes, and sampled 8,000 instances that follow the same distance to target gene distribution as significant eQTLs.

We also analyzed whether genes or elements with particular patterns of activity across biosamples behaved differently with respect to whether their GLS coefficients predict the results of CRISPR perturbations in K562 cells (**Supplementary Fig. N3.1f-k**). We defined a gene as "broadly expressed" if its DNase-seq signal value was above 2 in all 96 biosamples, and "variably expressed" otherwise. We defined a candidate element as "broadly active" if it showed DNase-seq signal above 1.5 in at least 3 of 96 biosamples, and "specifically active" otherwise. We selected these thresholds to obtain the similar amounts of CRISPR positive enhancer-promoter pairs across different combinations of promoter expression and enhancer activities.

31. Data visualization

Plots were generated in R (v4.2.0) using the following packages: tidyverse (v1.3.2), ggplot2 (v3.5.1), ggextra (v0.10.1), ggpubr (v0.6.0), ggcorrplot (v0.1.4.1), ggrastr (v1.0.2), cowplot (v1.1.3), Gviz (v1.42.0), GenomicInteractions (v1.32.0).

Locus plots in Figs. 1, 3, 4, Extended Data Fig. 6 and Supplementary Fig. 1 were created using the GViz (1.42.0) and GenomicInteractions (1.32.0) packages for R (4.2.0). Locus plots for Fig. 5, and Supplementary Figs. N1.3 and N5.2 were created using the IGV genome browser[69] (https://igv.org/app). Arcs in all locus plots show predicted E-G interactions above the chosen model thresholds.

Box plots are defined as follows: the middle line corresponds to the median; lower and upper hinges of the box correspond to 25th and 75th percentiles; the upper whisker extends from the hinge to the largest value no further than 1.5 × IQR from the hinge (where IQR is the interquartile range, or distance between the 25th and 75th percentile); and the lower whisker extends from the hinge to the smallest value, at most 1.5 × IQR of the hinge. Points beyond the end of the whiskers are outlying points and are plotted individually[70], unless box plots are shown on top of all data points or data distribution.

Supplementary References

1. Fulco, C. P. *et al.* Activity-by-contact model of enhancer-promoter regulation from thousands of CRISPR perturbations. *Nat. Genet.* **51**, 1664–1669 (2019).
2. Fulco, C. P. *et al.* Systematic mapping of functional enhancer-promoter connections with CRISPR interference. *Science* **354**, 769–773 (2016).
3. Gasperini, M. *et al.* A Genome-wide Framework for Mapping Gene Regulation via Cellular Genetic Screens. *Cell* **176**, 377–390.e19 (2019).
4. Schraivogel, D. *et al.* Targeted Perturb-seq enables genome-scale genetic screens in single cells. *Nat. Methods* **17**, 629–635 (2020).
5. Xie, S., Armendariz, D., Zhou, P., Duan, J. & Hon, G. C. Global Analysis of Enhancer Targets Reveals Convergent Enhancer-Driven Regulatory Modules. *Cell Rep.* **29**, 2570–2578.e5 (2019).
6. Klann, T. S. *et al.* Genome-wide annotation of gene regulatory elements linked to cell fitness. *bioRxiv* 2021.03.08.434470 (2021) doi:10.1101/2021.03.08.434470.
7. Morris, J. A. *et al.* Discovery of target genes and pathways at GWAS loci by pooled single-cell CRISPR screens. *Science* **380**, eadh7699 (2023).
8. Guckelberger, P. *et al.* Cohesin-mediated 3D contacts tune enhancer-promoter regulation. *bioRxivorg* 2024.07.12.603288 (2024).
9. Nasser, J. *et al.* Genome-wide enhancer maps link risk variants to disease genes. *Nature* **593**, 238–243 (2021).
10. Ray, J. *et al.* An unbiased survey of distal element-gene regulatory interactions with direct-capture targeted Perturb-seq. *bioRxiv* 2025.09.16.676677 (2025) doi:10.1101/2025.09.16.676677.
11. Reilly, S. K. *et al.* Direct characterization of cis-regulatory elements and functional dissection of complex genetic associations using HCR–FlowFISH. *Nat. Genet.* 1166–1176 (2021).
12. Lin, X. *et al.* Nested epistasis enhancer networks for robust genome regulation. *Science* **377**, 1077–1085 (2022).
13. Thakore, P. I. *et al.* Highly specific epigenome editing by CRISPR-Cas9 repressors for silencing of distal regulatory elements. *Nat. Methods* **12**, 1143–1149 (2015).
14. Ulirsch, J. C. *et al.* Systematic functional dissection of common genetic variation affecting red blood cell traits. *Cell* **165**, 1530–1545 (2016).
15. Wakabayashi, A. *et al.* Insight into GATA1 transcriptional activity through interrogation of cis elements disrupted in human erythroid disorders. *Proc. Natl. Acad. Sci. U. S. A.* **113**, 4434–4439 (2016).
16. Xu, J. *et al.* Developmental control of polycomb subunit composition by GATA factors mediates a switch to non-canonical functions. *Mol. Cell* **57**, 304–316 (2015).
17. Carleton, J. B., Berrett, K. C. & Gertz, J. Multiplex enhancer interference reveals collaborative control of gene regulation by estrogen receptor α-bound enhancers. *Cell Syst.* **5**, 333–344.e5 (2017).
18. Creyghton, M. P. *et al.* Histone H3K27ac separates active from poised enhancers and predicts developmental state. *Proc. Natl. Acad. Sci. U. S. A.* **107**, 21931–21936 (2010).
19. Kagda, M. S. *et al.* Data navigation on the ENCODE portal. *Nat. Commun.* **16**, 9592 (2025).
20. Popay, T. M. & Dixon, J. R. Coming full circle: On the origin and evolution of the looping model for enhancer-promoter communication. *J. Biol. Chem.* **298**, 102117 (2022).
21. Thurman, R. E. *et al.* The accessible chromatin landscape of the human genome. *Nature* **489**, 75–82 (2012).
22. Liu, Y., Sarkar, A., Kheradpour, P., Ernst, J. & Kellis, M. Evidence of reduced recombination rate in human regulatory domains. *Genome Biol.* **18**, 193 (2017).
23. Pliner, H. A. *et al.* Cicero Predicts cis-Regulatory DNA Interactions from Single-Cell Chromatin Accessibility Data. *Mol. Cell* **71**, 858–871.e8 (2018).
24. Bergman, D. T. *et al.* Compatibility rules of human enhancer and promoter sequences. *Nature* **607**, 176–184 (2022).

25. Martinez-Ara, M., Comoglio, F., van Arensbergen, J. & van Steensel, B. Systematic analysis of intrinsic enhancer-promoter compatibility in the mouse genome. *Mol. Cell* **82**, 2519–2531.e6 (2022).
26. GTEx Consortium. The GTEx Consortium atlas of genetic regulatory effects across human tissues. *Science* **369**, 1318–1330 (2020).
27. O'Connor, L. J. *et al.* Extreme Polygenicity of Complex Traits Is Explained by Negative Selection. *Am. J. Hum. Genet.* **105**, 456–476 (2019).
28. Vierstra, J. *et al.* Global reference mapping of human transcription factor footprints. *Nature* **583**, 729–736 (2020).
29. Abramov, S. *et al.* Landscape of allele-specific transcription factor binding in the human genome. *Nat. Commun.* **12**, 2751 (2021).
30. Chen, M. *et al.* Grab regulates transferrin receptor recycling and iron uptake in developing erythroblasts. *Blood* **140**, 1145–1155 (2022).
31. Levy, J. E., Jin, O., Fujiwara, Y., Kuo, F. & Andrews, N. C. Transferrin receptor is necessary for development of erythrocytes and the nervous system. *Nat. Genet.* **21**, 396–399 (1999).
32. Ohneda, O., Yanai, N. & Obinata, M. Combined action of c-kit and erythropoietin on erythroid progenitor cells. *Development* **114**, 245–252 (1992).
33. Paquette, R. L., Hsu, N. C. & Koeffler, H. P. Analysis of c-kit gene integrity in aplastic anemia. *Blood Cells Mol. Dis.* **22**, 159–168 (1996).
34. Bauer, D. E. *et al.* An erythroid enhancer of BCL11A subject to genetic variation determines fetal hemoglobin level. *Science* **342**, 253–257 (2013).
35. Canver, M. C. *et al.* BCL11A enhancer dissection by Cas9-mediated in situ saturating mutagenesis. *Nature* **527**, 192–197 (2015).
36. Niculescu-Mizil, A. & Caruana, R. Predicting good probabilities with supervised learning. in *Proceedings of the 22nd international conference on Machine learning - ICML '05* (ACM Press, New York, New York, USA, 2005). doi:10.1145/1102351.1102430.
37. Ustun, B. & Rudin, C. Learning optimized risk scores. *J. Mach. Learn. Res.* **20**, 1–75 (2019).
38. Seal, R. L. *et al.* Genenames.org: the HGNC resources in 2023. *Nucleic Acids Res* **51**, D1003–D1009 (2023).
39. Boix, C. A., James, B. T., Park, Y. P., Meuleman, W. & Kellis, M. Regulatory genomic circuitry of human disease loci by integrative epigenomics. *Nature* **590**, 300–307 (2021).
40. Nurtdinov, R. N. & Guigó, R. EPIraction - an atlas of candidate enhancer-gene interactions in human tissues and cell lines. *Bioinformatics* (2025).
41. Karbalayghareh, A., Sahin, M. & Leslie, C. S. Chromatin interaction–aware gene regulatory modeling with graph attention networks. *Genome Res.* **32**, 930–944 (2022).
42. Sahin, M. *et al.* HiC-DC+ enables systematic 3D interaction calls and differential analysis for Hi-C and HiChIP. *Nat. Commun.* **12**, 3366 (2021).
43. Avsec, Ž. *et al.* Effective gene expression prediction from sequence by integrating long-range interactions. *Nat. Methods* **18**, 1196–1203 (2021).
44. Luo, R. *et al.* Dynamic network-guided CRISPRi screen identifies CTCF-loop-constrained nonlinear enhancer gene regulatory activity during cell state transitions. *Nat. Genet.* **55**, 1336–1346 (2023).
45. Andersson, R. *et al.* An atlas of active enhancers across human cell types and tissues. *Nature* **507**, 455–461 (2014).
46. Granja, J. M. *et al.* Single-cell multiomic analysis identifies regulatory programs in mixed-phenotype acute leukemia. *Nat. Biotechnol.* **37**, 1458–1465 (2019).
47. Gao, T. & Qian, J. EnhancerAtlas 2.0: an updated resource with enhancer annotation in 586 tissue/cell types across nine species. *Nucleic Acids Res.* **48**, D58–D64 (2020).
48. Sheffield, N. C. *et al.* Patterns of regulatory activity across diverse human cell types predict tissue identity, transcription factor binding, and long-range interactions. *Genome Res.* **23**, 777–788 (2013).

49. Cao, Q. *et al.* Reconstruction of enhancer–target networks in 935 samples of human primary cells, tissues and cell lines. *Nat. Genet.* **49**, 1428–1436 (2017).
50. Whalen, S., Truty, R. M. & Pollard, K. S. Enhancer–promoter interactions are encoded by complex genomic signatures on looping chromatin. *Nat. Genet.* **48**, 488–496 (2016).
51. Zacher, B. *et al.* Accurate Promoter and Enhancer Identification in 127 ENCODE and Roadmap Epigenomics Cell Types and Tissues by GenoSTAN. *PLoS One* **12**, e0169249 (2017).
52. Finak, G. *et al.* MAST: a flexible statistical framework for assessing transcriptional changes and characterizing heterogeneity in single-cell RNA sequencing data. *Genome Biol.* **16**, 278 (2015).
53. Love, M. I., Huber, W. & Anders, S. Moderated estimation of fold change and dispersion for RNA-seq data with DESeq2. *Genome Biol.* **15**, 550 (2014).
54. Finucane, H. K. *et al.* Partitioning heritability by functional annotation using genome-wide association summary statistics. *Nat. Genet.* **47**, 1228–1235 (2015).
55. Gazal, S. *et al.* Linkage disequilibrium–dependent architecture of human complex traits shows action of negative selection. *Nat. Genet.* **49**, 1421–1427 (2017).
56. Weeks, E. M. *et al.* Leveraging polygenic enrichments of gene features to predict genes underlying complex traits and diseases. *Nat. Genet.* **55**, 1267–1276 (2023).
57. McLaren, W. *et al.* The Ensembl Variant Effect Predictor. *Genome Biol.* **17**, 122 (2016).
58. Wang, G., Sarkar, A., Carbonetto, P. & Stephens, M. A Simple New Approach to Variable Selection in Regression, with Application to Genetic Fine Mapping. *J. R. Stat. Soc. Series B Stat. Methodol.* **82**, 1273–1300 (2020).
59. de Leeuw, C. A., Mooij, J. M., Heskes, T. & Posthuma, D. MAGMA: generalized gene-set analysis of GWAS data. *PLoS Comput. Biol.* **11**, e1004219 (2015).
60. Morgens, D. W. *et al.* Genome-scale measurement of off-target activity using Cas9 toxicity in high-throughput screens. *Nature Communications* vol. 8 Preprint at https://doi.org/10.1038/ncomms15178 (2017).
61. Langmead, B. & Salzberg, S. L. Fast gapped-read alignment with Bowtie 2. *Nat. Methods* **9**, 357–359 (2012).
62. Danecek, P. *et al.* Twelve years of SAMtools and BCFtools. *Gigascience* **10**, (2021).
63. Klann, T. S. *et al.* CRISPR–Cas9 epigenome editing enables high-throughput screening for functional regulatory elements in the human genome. *Nat. Biotechnol.* **35**, 561–568 (2017).
64. Engreitz, J. M. *et al.* Local regulation of gene expression by lncRNA promoters, transcription and splicing. *Nature* **539**, 452–455 (2016).
65. Fuentes, D. R., Swigut, T. & Wysocka, J. Systematic perturbation of retroviral LTRs reveals widespread long-range effects on human gene regulation. *Elife* **7**, (2018).
66. Delaneau, O. *et al.* Chromatin three-dimensional interactions mediate genetic effects on gene expression. *Science* **364**, (2019).
67. Huang, J. *et al.* Dissecting super-enhancer hierarchy based on chromatin interactions. *Nat. Commun.* **9**, 943 (2018).
68. Kerimov, N. *et al.* A compendium of uniformly processed human gene expression and splicing quantitative trait loci. *Nat. Genet.* **53**, 1290–1299 (2021).
69. Robinson, J. T. *et al.* Integrative genomics viewer. *Nat. Biotechnol.* **29**, 24–26 (2011).
70. Wickham, H. *ggplot2: Elegant Graphics for Data Analysis*. (Springer New York, 2009).
