# Supplementary material for: An encyclopedia of human enhancer–gene regulatory interactions
Source: Nature. 2026 Jul 15;657(8130):179–90. doi: 10.1038/s41586-026-10781-4 (PMC13471189; doi:10.1038/s41586-026-10781-4)
Supplement: Supplementary file 2 [file 41586_2026_10781_MOESM2_ESM.pdf]

nature portfolio

Corresponding author(s): Jesse Engreitz

Last updated by author(s): May 29, 2026

Reporting Summary

Nature Portfolio wishes to improve the reproducibility of the work that we publish. This form provides structure for consistency and transparency in reporting. For further information on Nature Portfolio policies, see our Editorial Policies and the Editorial Policy Checklist.

Statistics

For all statistical analyses, confirm that the following items are present in the figure legend, table legend, main text, or Methods section.

n/a	Confirmed	
☐	☒	The exact sample size (*n*) for each experimental group/condition, given as a discrete number and unit of measurement
☐	☒	A statement on whether measurements were taken from distinct samples or whether the same sample was measured repeatedly
☐	☒	The statistical test(s) used AND whether they are one- or two-sided<br>*Only common tests should be described solely by name; describe more complex techniques in the Methods section.*
☐	☒	A description of all covariates tested
☐	☒	A description of any assumptions or corrections, such as tests of normality and adjustment for multiple comparisons
☐	☒	A full description of the statistical parameters including central tendency (e.g. means) or other basic estimates (e.g. regression coefficient) AND variation (e.g. standard deviation) or associated estimates of uncertainty (e.g. confidence intervals)
☐	☒	For null hypothesis testing, the test statistic (e.g. *F*, *t*, *r*) with confidence intervals, effect sizes, degrees of freedom and *P* value noted<br>*Give P values as exact values whenever suitable.*
☒	☐	For Bayesian analysis, information on the choice of priors and Markov chain Monte Carlo settings
☒	☐	For hierarchical and complex designs, identification of the appropriate level for tests and full reporting of outcomes
☐	☒	Estimates of effect sizes (e.g. Cohen's *d*, Pearson's *r*), indicating how they were calculated

Our web collection on statistics for biologists contains articles on many of the points above.

Software and code

Policy information about availability of computer code

Data collection	All published, open-source code. CRISPRi-FlowFISH data from the MYC enhancer synergy screen was collected using CytExpert. Other data was downloaded from ENCODE portal and public sources using wget (v1.21.4).
Data analysis	Programming and workflow languages: Python (v3.8, v3.12.3, v3.9.1), R (v3.6.1, v4.1.1, v4.2.0), Snakemake (v7 or greater)<br><br>NGS data processing: samtools (v1.6, v1.7), Picard MarkDuplicates (v1.126), BLAT (UCSC), bedtools (v2.26.0), MACS2 (v2.2.9.1), UCSC bigWigAverageOverBed (v366), chromHMM (v1.22), HiC-DC+ (v0.99.14)<br><br>Single-cell and differential expression analysis (R packages): Seurat (v5.0.1), SCEPTRE (v0.10.0), MAST (v1.20.0), DESeq2 (v1.34.0)<br><br>Genome annotations and reference resources: easylift R package (v1.0.0), UCSC liftOver (v377), RepeatMasker database (v4.0.5), Variant Effect Predictor (v85)<br><br>Statistical analysis and benchmarking (R packages): nlme (v3.1-162), boot (v1.3-28.1), boot.pval (v0.7.0), ROCR (v1.0-11), caTools (v1.18.2)<br><br>Statistical genetics and GWAS analysis: MAGMA (v1.08), S-LDSC (v1.0), PoPs (v0.2)<br><br>CRIPSRi-FlowFISH screen design and data processing: GuideScan (v1.2) FlowJo (v10), Bowtie 2 (v2.4.2), FCSalyzer (v0.9.22-alpha), stats4 R package (R v3.6.1)<br><br>Data analysis and visualization (R packages): tidyverse (v1.3.2), data.table (v1.15.2), ggplot2 (v3.5.1), ggextra (v0.10.1), ggpubr (v0.6.0),

ggcorrplot (v0.1.4.1), ggrastr (v1.0.2), cowplot (v1.1.3), Gviz (v1.42.0), GenomicInteractions (v1.32.0)

Data analysis workflows and E-G prediction pipelines can be found on github.com:
ENCODE-rE2G (v1.0.0): https://github.com/EngreitzLab/ENCODE_rE2G
ABC (v1.1.2): https://github.com/broadinstitute/ABC-Enhancer-Gene-Prediction
GraphReg (v1): https://github.com/karbalayghareh/GraphReg
EPIraction (v1.10.1): https://github.com/guigolab/EPIraction
CIA and CCD features: https://github.com/wangxi001/CIA
EpiMap: https://github.com/KellisLab/EpiMap_GRCh38_linking
CRISPR benchmarking pipeline (v1.0.0): https://github.com/EngreitzLab/CRISPR_comparison
eQTL benchmarking pipeline: https://github.com/EngreitzLab/eQTLEnrichment
GWAS benchmarking pipeline: https://github.com/Deylab999MSKCC/e2g-benchmarking
Combined K562 CRISPR data (training data) processing (v1.0.1): https://github.com/argschwind/ENCODE_CRISPR_data
Held-out CRISPR data processing: https://github.com/EngreitzLab/ENCODE_Test_Dataset_Analysis
Manuscript analysis code: https://github.com/EngreitzLab/ENCODE-Distal-Regulation-Paper

For manuscripts utilizing custom algorithms or software that are central to the research but not yet described in published literature, software must be made available to editors and reviewers. We strongly encourage code deposition in a community repository (e.g. GitHub). See the Nature Portfolio guidelines for submitting code & software for further information.

Data

Policy information about availability of data

All manuscripts must include a data availability statement. This statement should provide the following information, where applicable:

- Accession codes, unique identifiers, or web links for publicly available datasets
- A description of any restrictions on data availability
- For clinical datasets or third party data, please ensure that the statement adheres to our policy

ENCODE-rE2G predictions and input epigenomics dataset are available on the ENCODE Portal (www.encodeproject.org). ENCODE file accession ids for predictions and inputs are listed in Supplementary Table 12. The aggregated Hi-C Megamap is available on the ENCODE Portal under ENCSR492BKE.

CTCF ChIA-PET data used to compute CTCF features is available on the ENCODE Portal under ENCSR184YZV and ENCSR597AKG, and Hi-C data used to compute Hi-C loop features is available under ENCFF134HIZ and ENCFF173VDJ.

ENCODE portal accession ids for GraphReg and EPIraction predictions are available in Supplementary Tables 18 and 19, respectively.

The combined K562 CRISPR dataset used to train ENCODE-rE2G is available at https://github.com/EngreitzLab/CRISPR_comparison/blob/v1.0.0/resources/crispr_data/EPCrisprBenchmark_combined_data.training_K562.GRCh38.tsv.gz and at https://www.synapse.org/Synapse:syn70814727 for a version including all ENCODE-rE2GExtended features. The dataset is also available on the ENCODE portal under ENCSR998YDI.

The held-out CRISPR dataset is available at https://github.com/EngreitzLab/CRISPR_comparison/blob/v1.0.0/resources/crispr_data/EPCrisprBenchmark_combined_data.heldout_5_cell_types.GRCh38.tsv.gz.

CRISPR enhancer perturbation data for the individual datasets is available under: GSE120861 (Gasperini et al., 2019), GSE135497 (Schraivogel et al., 2020), https://github.com/EngreitzLab/ABC-GWAS-Paper (Nasser et al., 2021), GSE171452 (Morris et al., 2023), GSE129837 (Xie et al., 2019), ENCFF904ZDX (Klann et al., 2021).

The ENCODE K562 DNase-seq experiment used to define candidate elements for CRISPR Perturb-seq data re-analyses is available on the ENCODE portal under ENCSR000EOT

For the eQTL benchmarking pipeline, GTEx eQTL variants and RNA expression data for each tissue used in the eQTL benchmarking pipeline are available on Synapse: https://www.synapse.org/Synapse:syn70198274.

For the GWAS benchmarking pipeline, fine-mapped GWAS data for UK Biobank traits is available from https://www.finucanelab.org/data.

Files containing baseline predictors used for benchmarking analyses can be found on Synapse: https://www.synapse.org/Synapse:syn58896208.

Data from the pairwise CRISPRi enhancer perturbation experiment at the MYC locus in K562 is available on the ENCODE portal under ENCSR443VTK. The used gRNA library is available under ENCFF118BUP.

H3K27ac ChIP-seq data from Huang et al., 2018 used in enhancer synergy analyses is available from GEO: GSE107726.

CRISPRi-H3K27ac ChIP-seq experiments at the MYC locus are available from GEO: GSE225157.

Research involving human participants, their data, or biological material

Policy information about studies with human participants or human data. See also policy information about sex, gender (identity/presentation), and sexual orientation and race, ethnicity and racism.

Reporting on sex and gender	In the design and execution of this study, sex (as a biological attribute) and gender (shaped by social and cultural circumstances) were not explicitly considered variables. Consequently, data related to sex and gender were not collected, and therefore, no sex- and gender-based analyses were performed.
Reporting on race, ethnicity, or	In the design and execution of this study, we did not utilize any socially constructed or socially relevant categorization variables, such as race, ethnicity, gender, or other socially relevant groupings.

other socially relevant groupings	
Population characteristics	In the design and execution of this study, we did not collect or analyze data on covariate-relevant population characteristics such as age, genotypic information, past and current diagnoses, and treatment categories.
Recruitment	Our study did not involve the recruitment of participants.
Ethics oversight	Our research focuses on developing enhancer-gene linking models and utilizes computational methods and publicly available datasets. Given the nature of our work, our study does not involve direct interaction with human or animal subjects nor the use of personalized genetic information that could potentially identify individuals.

Note that full information on the approval of the study protocol must also be provided in the manuscript.

Field-specific reporting

Please select the one below that is the best fit for your research. If you are not sure, read the appropriate sections before making your selection.

☒ Life sciences ☐ Behavioural & social sciences ☐ Ecological, evolutionary & environmental sciences

For a reference copy of the document with all sections, see nature.com/documents/nr-reporting-summary-flat.pdf

Life sciences study design

All studies must disclose on these points even when the disclosure is negative.

Sample size	For the MYC CRISPRi-FlowFISH experiments, sample size (5 million sorted cells per replicate) was chosen based on power calculations from previous CRISPRi-FlowFISH experiments (Fulco et al., 2019).<br><br>For the H3K27ac ChIP-seq experiments following CRISPRi perturbations, 3-4 ChIP-seq experiments were performed per perturbed element (MYC enhancers e1-e7, NS1) and 16 experiments using one of 4 negative control guides. The number of experiments per perturbed element was defined based on established protocols from previous studies<br><br>No other experiments were performed and all other data was taken from published and/or publicly available sources.
Data exclusions	No data was excluded from the analysis of the MYC CRISPRi-FlowFISH and H3K27ac ChIP-seq experiments.
Replication	MYC CRISPRi-FlowFISH experiments were performed in 2 successful biological replicate.<br><br>H3K27ac ChIP-seq experiments following CRISPRi perturbations were performed for 2 technical replicates on each of 2 biological replicates. All replicate experiments were successful.
Randomization	For CRISPRi-FlowFISH experiments, perturbations were applied to all cells in a pooled screen format, making allocation into experimental groups inapplicable.<br><br>Cells for H3K27ac experiments were selected randomly for perturbations by the nature of the protocol and assayed in parallel without experimental groups requiring randomized allocation.
Blinding	The readout for the CRISPRi-FlowFISH experiments is guide RNA abundance per bin quantified by next-generation sequencing, which is an objective, automated measurement that does not involve subjective evaluation or operator judgment.<br><br>H3K27ac ChIP-seq data was generated by next-generation sequencing and analyzed using a predefined analysis pipeline, eliminating the possibility of investigator bias during data acquisition or analysis.

Reporting for specific materials, systems and methods

We require information from authors about some types of materials, experimental systems and methods used in many studies. Here, indicate whether each material, system or method listed is relevant to your study. If you are not sure if a list item applies to your research, read the appropriate section before selecting a response.

Materials & experimental systems

n/a	Involved in the study	
☒	☐	Antibodies
☐	☒	Eukaryotic cell lines
☒	☐	Palaeontology and archaeology
☒	☐	Animals and other organisms
☒	☐	Clinical data
☒	☐	Dual use research of concern
☒	☐	Plants

Methods

n/a	Involved in the study	
☒	☐	ChIP-seq
☐	☒	Flow cytometry
☒	☐	MRI-based neuroimaging

Eukaryotic cell lines

Policy information about cell lines and Sex and Gender in Research

Cell line source(s)	K562 cells were purchased from ATCC and previously engineered to express KRAB-dCas9. HEK293T cells were purchased from ATCC.
Authentication	Cell lines were authenticated through analysis of epigenomic data
Mycoplasma contamination	All cell lines tested negative for mycoplasma
Commonly misidentified lines (See ICLAC register)	No commonly misidentified lines were used

Plants

Seed stocks	N/A
Novel plant genotypes	N/A
Authentication	N/A

Flow Cytometry

Plots

Confirm that:

☒ The axis labels state the marker and fluorochrome used (e.g. CD4-FITC).

☒ The axis scales are clearly visible. Include numbers along axes only for bottom left plot of group (a 'group' is an analysis of identical markers).

☒ All plots are contour plots with outliers or pseudocolor plots.

☒ A numerical value for number of cells or percentage (with statistics) is provided.

Methodology

Sample preparation	For scoring and sorting BFP+ CRISPRi cells after doxycycline treatement, cells were harvested and resuspended in EC medium at a density of ~1-3e6 cells/ml. FlowFISH was performed according to our established methods (Fulco, C. P., Nasser, J., Jones, T. R. & Munson, G. Activity-by-contact model of enhancer–promoter regulation from thousands of CRISPR perturbations. Nature Genetics 51, 1664–1669 (2019). Briefly, we used the PrimeFlow RNA Assay Kit (Thermo Fisher; Catalog number: 88-18005) according to the manufacturer's instructions, using ThermoFisher probesets VA1-3010837-PF for TLNRD1 and VA4-13187-PF for RPL13A. The TLNRD1 stain was Alexa Fluor 647 (AF647, "Type 1"), while the RPL13A stain was Alexa Fluor 488 (AF488, "Type 4"). We diluted the stained and washed cells in PBS with 0.5% BSA to a concentration of 2x10^7 cells/ml and filtered using a 30µm filter (CellTrics, Catalog number 04-004-2326).
Instrument	Diagnostic FACS for BFP positive cells was performed on a Beckman CytoFLEX S Flow Cytometer. The sort for BFP+ cells was performed on a MoFlo Astrios EQ Cell Sorter. For FlowFISH, we used a SONY MA900 sorter.
Software	FACS runs were performed using the manufacturer software package (e.g. CytExpert for diagonstic FACS). Basic analysis was performed using FlowJo or FCSalyzer 0.9.22-alpha. FlowFISH analysis was performed using a Maximum Likelihood Estimator

approach, according to our established methods (Fulco et al. Nature Genetics 51, 1664–1669 (2019). In brief, we counted gRNAs in each bin using Bowtie to map reads to a custom index, normalized gRNA counts in each bin by library size, then used a maximum-likelihood estimation approach to compute the effect size for each gRNA. We used the limited-memory Broyden–Fletcher–Goldfarb–Shanno algorithm (implemented in the R stats4 package) to estimate the most likely log-normal distribution that would have produced the observed guide counts, and the effect size for each gRNA is the mean of its log-normal fit divided by the average of the means from all negative-control gRNAs. As previously described, we scaled the effect size of each gRNA in a screen linearly, so that the strongest 20-guide window at the TSS of the target gene has an 85% effect, in order to account for non-specific probe binding in the RNA FISH assay (this is based on our observation that promoter CRISPRi typically shows 80–90% knockdown by qPCR). We averaged the effect sizes of each gRNA across replicates and computed the effect size of an element as the average of all gRNAs targeting that element. We assessed significance using a two-sided t-test comparing the mean effect size of all gRNAs in a candidate element to all negative-control guides. We computed the false-discovery rate (FDR) for elements using the Benjamini–Hochberg procedure and used an FDR threshold of 0.05 to call significant regulatory effects.

Cell population abundance

Cells were not a mix of cell types, and were all TeloHAEC. For BFP+ cell sorting, almost all cells had a stronger PB450-A signal than non-dox treated controls, and we sorted for cells with the top 15% PB450-A signal. For FlowFISH cells in the gRNA library-transduced pool with different CRISPRi perturbation guides were sorted into bins by percentage of normalized TLNRD1/RPL13A signal, as described below.

Gating strategy

For sorting BFP+ cells, live cells were gated by SSC-A and FSC-A. BFP-positive cells were identified by comparison to no Doxycycline control cells, and the top 15% of the BFP-positive peak (PB450-A) selected. FlowFISH parameters were essentially as per Fulco et al. Nature Genetics 51, 1664–1669 (2019). Briefly, to control for differences in staining efficiency for each cell, we normalized the fluorescence associated with the gene of interest to that of the control gene. Specifically, we used the color compensation tool to subtract a portion of each cell's AF647 signal based on the intensity of its AF488 signal such that the mean AF488 signal in the top and bottom 25% of cells based on AF647 was within 10%. If necessary, we then reduced the level of compensation until the fraction of cells with AF647 signal
equal to 0 was no more than 5%. We set the gates for each bin on the compensated signal to capture quartiles of cells, with bins numbered according to the percentiles (i) 0-25% (ii) 25-50%, (iii) 50-75%, (iv) 75-100%.

☒ Tick this box to confirm that a figure exemplifying the gating strategy is provided in the Supplementary Information.
