# Supplementary material for: An encyclopedia of human enhancer–gene regulatory interactions
Source: Nature. 2026 Jul 15;657(8130):179–90. doi: 10.1038/s41586-026-10781-4 (PMC13471189; doi:10.1038/s41586-026-10781-4)
Supplement: Supplementary file 3 [file 41586_2026_10781_MOESM3_ESM.zip › 2023-11-20318B-s3/Supplementary_table_legends.docx]

**Supplementary Information Guide**

## Supplementary Table 1.xlsx

A list of enhancer-gene linking methods collected as part of this study. This table also contains parameters used for benchmarking analyses.

## Supplementary Table 2.xlsx

A list of all molecular features of enhancers, promoters and enhancer-promoter pairs collected as part of this study to develop ENCODE-rE2G and ENCODE-rE2G*^Extended^*.

## Supplementary Table 3.xlsx

Performance of predictive models at predicting CRISPR E-G pairs in the CRISPR benchmarking analyses. Columns show area under the precision-recall curve (AUPRC), threshold at 70% recall and precision at the chosen threshold, including 95% value intervals for AUPRC and precision from bootstrapping.

## Supplementary Table 4.xlsx

Results of pairwise comparisons of performance metrics in the CRISPR benchmarking analyses. Columns show compared models, performance differences, and 95% confidence intervals, minimum, maximum values and *P-*values from bootstrapping differences (delta) in performance metric.

## Supplementary Table 5.xlsx

Tissue-biosample pairs and numerical results from benchmarking predictive models of enhancer-gene regulatory interactions against finemapped eQTL variants.

## Supplementary Table 6.xlsx

List of traits and studies used for benchmarking models enhancer-gene regulatory interactions against fine-mapped GWAS variants.

## Supplementary Table 7.xlsx

Numerical results from benchmarking predictive models of enhancer-gene regulatory interactions against fine-mapped GWAS variants.

## Supplementary Table 8.xlsx

Summary statistics for binary ENCODE-rE2G predictions of regulatory enhancer-gene interactions in 1,458 DNase-seq experiments.

## Supplementary Table 9.xlsx

Summary statistics per gene from binary ENCODE-rE2G predictions of regulatory enhancer-gene interactions in 1,458 DNase-seq experiments.

## Supplementary Table 10.xlsx

List of OpenTarget study IDs used to benchmark the Locus-to-Gene (L2G) approach and matching GWAS traits used in this study.

## Supplementary Table 11.xlsx

Effects of individual enhancer perturbations (percent change of *MYC* expression), effect of pairwise enhancer interactions (pairwise effects - individual effects; positive = super-additive effect), *P-*values and Benjamini-Hochberg adjusted *P-*values for enhancer interactions (**Supplementary Methods 29**).

## Supplementary Table 12.xlsx

ENCODE metadata for ENCODE-rE2G and ENCODE-rE2G*^Extended^* models including Accession IDs for final predictions on the ENCODE portal.

## Supplementary Table 13.xlsx

ENCODE Accession IDs or URLs for input data used to compute features to train ENCODE-rE2G and ENCODE-rE2G*^Extended^* models in K562, and apply ENCODE-rE2G*^Extended^* in K562 and GM12878.

## Supplementary Table 14.xlsx

ENCODE Accession IDs for all DNase-seq files used as input data to compute correlation metrics between DNase-seq signal at cCREs and promoters. See Supplementary Methods 7 ‘Correlation of enhancer-promoter activity across cell types’.

## Supplementary Table 15.xlsx

ENCODE Accession IDs for all DNase-seq and RNA-seq files used as input data to compute correlation metrics between DNase-seq signal at cCREs and RNA-seq expression levels from genes. See Supplementary Methods 7 ‘Correlation of enhancer-promoter activity across cell types’.

## Supplementary Table 16.xlsx

ENCODE Accession IDs for files used as input data to compute baseline predictors in 89 biosamples. Also contains urls for resulting baseline predictor files on www.synapse.org. See Supplementary Methods 16 ‘Generating baseline predictors’.

## Supplementary Table 17.xlsx

ENCODE Accession IDs for files used as input data to compute Activity-By-Contact modes using different chromatin assays to measure enhancer activity. See Supplementary Methods 26 ‘Additional analyses: Assaying enhancer activity’.

## Supplementary Table 18.xlsx

ENCODE Accession IDs for features and input data that were used as input for the GraphReg model and resulting predictions. See Supplementary Methods 11 ‘GraphReg’.

## Supplementary Table 19.xlsx

Metadata and ENCODE Accession IDs for EPIraction predictions in 79 different tissues and cell types. See Supplementary Methods 10 ‘EPIraction’.
